# NGF-Dependent Changes in Ubiquitin Homeostasis Trigger Early Cholinergic Degeneration in Cellular and Animal AD-Model

**DOI:** 10.3389/fncel.2018.00487

**Published:** 2018-12-13

**Authors:** Valentina Latina, Silvia Caioli, Cristina Zona, Maria Teresa Ciotti, Antonella Borreca, Pietro Calissano, Giuseppina Amadoro

**Affiliations:** ^1^European Brain Research Institute, Rome, Italy; ^2^IRCCS Santa Lucia Foundation, Rome, Italy; ^3^Department of Systems Medicine, University of Rome Tor Vergata, Rome, Italy; ^4^Institute of Cellular Biology and Neurobiology – National Research Council, Rome, Italy; ^5^Institute of Translational Pharmacology – National Research Council, Rome, Italy

**Keywords:** Alzheimer’s disease, nerve growth factor, cholinergic synapse(s), ubiquitin-proteasome system, ubiquitin-C-terminal hydrolase 1, neurodegeneration

## Abstract

Basal forebrain cholinergic neurons (BFCNs) depend on nerve growth factor (NGF) for their survival/differentiation and innervate cortical and hippocampal regions involved in memory/learning processes. Cholinergic hypofunction and/or degeneration early occurs at prodromal stages of Alzheimer’s disease (AD) neuropathology in correlation with synaptic damages, cognitive decline and behavioral disability. Alteration(s) in ubiquitin-proteasome system (UPS) is also a pivotal AD hallmark but whether it plays a causative, or only a secondary role, in early synaptic failure associated with disease onset remains unclear. We previously reported that impairment of NGF/TrkA signaling pathway in cholinergic-enriched septo-hippocampal primary neurons triggers “dying-back” degenerative processes which occur prior to cell death in concomitance with loss of specific vesicle trafficking proteins, including synapsin I, SNAP-25 and α-synuclein, and with deficit in presynaptic excitatory neurotransmission. Here, we show that in this *in vitro* neuronal model: (i) UPS stimulation early occurs following neurotrophin starvation (-1 h up to -6 h); (ii) NGF controls the steady-state levels of these three presynaptic proteins by acting on coordinate mechanism(s) of dynamic ubiquitin-C-terminal hydrolase 1 (UCHL-1)-dependent (mono)ubiquitin turnover and UPS-mediated protein degradation. Importantly, changes in miniature excitatory post-synaptic currents (mEPSCs) frequency detected in -6 h NGF-deprived primary neurons are strongly reverted by acute inhibition of UPS and UCHL-1, indicating that NGF tightly controls *in vitro* the presynaptic efficacy via ubiquitination-mediated pathway(s). Finally, changes in synaptic ubiquitin and selective reduction of presynaptic markers are also found *in vivo* in cholinergic nerve terminals from hippocampi of transgenic Tg2576 AD mice, even from presymptomatic stages of neuropathology (1-month-old). By demonstrating a crucial role of UPS in the dysregulation of NGF/TrkA signaling on properties of cholinergic synapses, these findings from two well-established cellular and animal AD models provide novel therapeutic targets to contrast early cognitive and synaptic dysfunction associated to selective degeneration of BFCNs occurring in incipient early/middle-stage of disease.

## Introduction

Basal forebrain cholinergic neurons -whose axons project to cortical mantle and hippocampus where they form the largest part of cholinergic synapses (McKinney et al., 1983; Sofroniew et al., 1990) involved in the regulation of synaptic activity and modulation of memory and attention (Conner et al., 2003, 2005; Hasselmo and Giocomo, 2006)- critically depend on retrograde transport of target-derived NGF for their survival, neurite outgrowth, phenotypic expression and maintenance (Hefti and Weiner, 1986; Hartikka and Hefti, 1988b; Debeir et al., 1999; Cuello et al., 2007). Experimental and clinical studies have clearly demonstrated that the cholinergic hypofunction and/or denervation due to imbalance in NGF/TrkA signaling pathway are causally related to MCI at prodromal stages of AD neuropathology (Mufson et al., 2000, 2002, 2008; Salehi et al., 2003; Niewiadomska et al., 2011; Schliebs and Arendt, 2011; Hampel et al., 2018). NGF replacement therapy turned out to be an effective disease-modifying treatment to improve the cholinergic deficits in humans affected from mild AD and, then, to prevent and/or delay the cognitive deterioration of symptomology (Tuszynski et al., 2005; Mufson et al., 2008). In this context, constant adaptations in the delivery mode to CNS and in the drug-design pharmacological approach with the development of TrkA mimetics have recently allowed to achieve significant results in increasing the NGF bioavailability to target neurons and/or in reducing its potential indirect and unwanted side-effects (Mufson et al., 2008) with consequent long-term improvement of the cholinotrophic basal forebrain function affected in AD patients (Mufson et al., 2008). Beyond the decline of cholinergic activity, the deficits in distribution, production and function of NGF have also been proved to be causative for the abnormal processing of the APP and tau dysmetabolism associated in the CNS with the two prominent clinico-pathological AD hallmarks, i.e., the deposits of Aβ and the tau-positive NFT. Therefore, advancements in NGF basic research are highly relevant for potential therapeutic implications in the field of AD neurodegeneration mainly for the most common, sporadic, late-onset forms (Cattaneo and Calissano, 2012; Triaca and Calissano, 2016; Canu et al., 2017).

The vulnerable, AD-affected neuronal populations early undergo a classic “dying-back” pattern of atrophy leading to retrograde degeneration of neural networks (Griffin and Watson, 1988; Mandelkow et al., 2003; Roy et al., 2005; Overk and Masliah, 2014) through which the loss in synaptic integrity and function(s) -the major biological correlate of cognitive impairment in AD (Terry et al., 1991; Selkoe, 2002; Coleman and Yao, 2003; Arendt, 2009)- long precedes somatic cell death (Campenot, 1982; Brady and Morfini, 2010). Compelling studies using *in vitro* compartmentalized chamber of sensory sympathetic neurons (Campenot, 1982) and NGF-deprived septo-hippocampal cholinergic-enriched primary neurons (Latina et al., 2017) support the pivotal role of deprivation from trophic support in triggering the “dying-back” axonal/synaptic pruning, in the absence of overt neuronal death. To this regard, the coordinated regulation between protein ubiquitylation and UPS-dependent degradation (Watts et al., 2003; Zhai et al., 2003; Hoopfer et al., 2006; Yaron and Schuldiner, 2016) has been indicated to play a physiopathological role in synapse(s) remodeling. Evidence has demonstrated that the proteasomal activity controls the formation/maintenance of synaptic connections and, then, the synaptic plasticity by locally regulating the abundance and/or distribution of different classes of pre- and postsynaptic proteins (Hegde, 2004; Patrick, 2006; Tai and Schuman, 2008; Segref and Hoppe, 2009). Furthermore, aberrations in the UPS have been implicated, directly or indirectly, in selective AD neuropathology featured by decreased synaptic density and subtle alterations in synaptic efficacy occurring prior to neuronal degeneration (Gadhave et al., 2016). Pharmacological and genetic inhibition of proteasomal function(s) has been shown to protect sympathetic neurons -such as superior ganglia, dorsal root ganglion neurons and retinal ganglion cells- following *in vitro* NGF withdrawal (Sadoul et al., 1996; Zhai et al., 2003; MacInnis and Campenot, 2005) and to delay degeneration of crushed optic nerves *in vivo* (Zhai et al., 2003). Conversely, the stimulation of neuritogenesis and synaptic differentiation in NGF-exposed pheochromocytoma PC12 cell-line is accompanied by an upregulation of the endogenous rate of cellular ubiquitylation (Obin et al., 1999) with accumulation of ubiquitin-conjugated proteins and coincident reductions in levels of free monomer (Takada et al., 1994; Ohtani-Kaneko et al., 1996). Additional evidence pointing to a potential role of ubiquitin dyshomeostasis in triggering an AD-like “dying-back”-type neurodegenerative phenotype comes from UCHL-1 (PGP9.5), a neuron-specific ubiquitin C-terminal hydrolase involved in disease pathogenesis (Pasinetti, 2001; Choi et al., 2004; Gong et al., 2006) which is enriched at nerve terminals (Liu et al., 2002) where it controls their physiopathological structural reshaping via ubiquitin recycling (Cartier et al., 2009). Spontaneous *gad* (gracile axonal dystrophy) mice carrying UCHL-1 deletion suffer an early (6 weeks old) retrograde synaptic/axonal degeneration with accumulation of APP/Aβ peptide(s) and ubiquitin into spheroids bodies (Ichihara et al., 1995; Saigoh et al., 1999) accompanied by impaired memory performance (Sakurai et al., 2008).

Altogether, these findings indicate a potential causal link between the dysregulation of ubiquitin homeostasis and/or of UPS enzymes activities and the early synaptic failure associated to alterations in NGF/TrkA signaling pathway in incipient AD neuropathology. However, whether alterations in UPS-dependent protein turnover actually mediate the NGF-induced changes in synaptic stability and function(s) in cholinergic-based cellular and animal AD models have not yet been investigated. Likewise, the time-window and the specific targets which are regulated by the UPS proteolysis at nerve cholinergic endings in response to NGF availability still remain to be investigated.

In this study, we explore whether changes in UPS activation underlie the neurotrophin-regulated functional elimination of synaptic contacts by turning to two well-established cellular and animal AD models such as cholinergic-enriched primary septo-hippocampal neurons- which undergo a “dying-back” degeneration following NGF withdrawal (Latina et al., 2017)- and aging (huAPP695.K670N/M671L)2576 (Tg2576) transgenic mice- which display reduced expression of NGF and its cognate receptor(s) alongside spatial memory deficits associated with degeneration of hippocampal cholinergic synapses (Zhu et al., 2017).

## Materials and Methods

### Reagents and Antibodies

MG132 (specific and cell-permeable proteasomal inhibitor, Myung et al., 2001) and LDN-57444 (specific and cell-permeable UCHL-1 inhibitor, Cartier et al., 2009) were purchased from Sigma-Aldrich (St. Louis, MO, United States) and were dissolved in DMSO. Bortezomib (specific and cell-permeable proteasomal inhibitor, Buac et al., 2013) was purchased from Calbiochem and was dissolved in DMSO. Chloroquine (an autophagy inhibitor; Klionsky et al., 2012) was purchased from Sigma-Aldrich and was dissolved in water. Z-VAD-FMK (carbobenzoxy-valyl-alanyl-aspartyl-[*O*-methyl]-fluoromethylketone) (cell-permeant pan caspase inhibitor) was purchased from Calbiochem and was dissolved in DMSO. NGF was purified from submaxillary glands (Bocchini and Angeletti, 1969). NGF from Xiamen Bioway (Biotech Co., Ltd., Xiamen, Fujian, China) was also used in the study.

Picrotoxin and strychnine were purchased from Sigma-Aldrich (St. Louis, MO, United States) and were dissolved in EtOH and water, respectively. Tetrodotoxin (TTX) was from Alomone Labs (Jerusalem, Israel) and was dissolved in water. The drugs were diluted to their final concentration with the extracellular solution. MG132 (10 μM) and LDN (2.5 μM) were added to the culture medium for 6 h prior the electrophysiological recordings.

The following antibodies were used: anti-ubiquitin antibody rabbit Z0458 Dako-Cytomation; anti-synapsin I antibody rabbit AB1543P Millipore; anti-SNAP25 antibody (clone SMI 81) mouse 836301 BioLegend; anti-α-synuclein antibody (clone 42) mouse 610786 BD Transduction Laboratories; anti-synaptophysin antibody (D-4) mouse sc-17750 Santa Cruz; anti-syntaxin 1 mouse S1172 Sigma-Aldrich; anti-TrkA antibody (763) rabbit sc-118 Santa Cruz; anti-choactase antibody (H-95) rabbit sc-20672 Santa Cruz; anti-mAChR M1 antibody (H120) rabbit sc-9106 Santa Cruz; anti-vGLUT1 antibody rabbit 135 302 Synaptic System; anti-vGAT antibody rabbit 131 003 Synaptic System; NMDAζ1 antibody (C-20) goat sc-1467 Santa Cruz; anti-β-Amyloid 1-16 antibody (clone 6E10) mouse Signet 932002; anti-APP 66-81 antibody (clone 22C11) mouse MAB348 Millipore; anti-LC-3 pAb antibody rabbit PD014 MBL; anti-β-actin antibody mouse S3062 Sigma-Aldrich; anti-UCHL-1 (C-4) antibody mouse sc-271639 Santa Cruz; anti-mouse IgG (whole molecule)-Peroxidase antibody A4416 Sigma-Aldrich; anti-rabbit IgG (whole molecule)-Peroxidase antibody A9169 Sigma-Aldrich; donkey anti-goat IgG-HRP antibody sc2056 Santa Cruz.

### Animals

All protocols (527/2017-PR) involving animals were performed in accordance with the guidelines established by the European Communities Council (Directive 2010/63/EU of 22 September 2010) regarding the care and use of animals for experimental procedures and with the Italian legislation on animal experimentation (Decreto L.vo 116/92).

Male mice overexpressing the APP695 fragment with the Swedish mutation (TgHuAPP695swe: Tg2576) in a hybrid genetic background (87% C57BL/6 × 12.5% SJL) were subsequently backcrossed to C57BL/6 × SJL F1 females. The offspring was genotyped to confirm the presence of human mutant APP DNA sequence by PCR. Each experiment was carried out in transgenic mice and WT mice of 1 and 9 months of age. Mice were maintained on a 12-h light/dark cycle with *ad libitum* access to food and water. All efforts were made to minimize the number of animals used and suffering.

### Primary Cultures of Cholinergic-Enriched Septo-hippocampal Neurons

Septal neurons were prepared from embryonic day 17/18 (E17/18) Wistar rats (Charles River Laboratories), as previously described (Hartikka and Hefti, 1988a,b) with some modifications. Briefly, embryos were surgically removed and septo-hippocampal areas were dissected from the cerebral tissue in ice-cold Hanks’ balanced salt solution (HBSS, Gibco, Life Technologies), freed of meninges, digested with 0.25% trypsin for 15 min at 37°C, dissociated by trituration and seeded as follows: 2 × 10^6^ cells on poly-l-lysine (Sigma-Aldrich)-coated plates (BD Falcon, Durham, NC, United States; 353001) for biochemistry analyses and 10 × 10^4^ cells on glass coverslips in 24-well plates (BD Falcon; 351147) for immunofluorescence analyses. The dissociated cells were plated in serum-free Neurobasal medium (Gibco, Life Technologies) supplemented with 0.2% B27 (Invitrogen Inc., Carlsbad, CA, United States) in the presence of NGF (100 ng/ml) for 10–12 days. One day after plating, cytosine arabinoside (5 mM) was added to inhibit glial proliferation. Cultures were kept at 37°C in a humidified incubator in a 5% CO_2_ atmosphere without further medium changes until used for experiments.

### Drug Treatment

Treatment of primary neurons with UPS or UCHL-1 inhibitors was performed in conditioned medium without preincubation. When cultures were cotreated with proteasomal inhibitors and UCHL-1 inhibitors (i.e., MG132+LDN), both inhibitors were added simultaneously. All inhibitors were diluted in conditioned medium from a 1,000× stock in DMSO.

### Assessment of Neuronal Viability

Cell viability (living/dead neurons) was quantified by DNA condensation-based assays with DAPI staining and by the MTT tetrazolium salt assay, as reported in Ramirez et al. (2010) and Corsetti et al. (2015).

### Immunofluorescence

Following treatment, septo-hippocampal cultures were washed twice with PBS and fixed in 4% (w/v) paraformaldehyde for 15 min at room temperature. Cells were permeabilized with 0.1% (v/v) Triton X-100/PBS pH 7.4 for 7 min at room temperature. Coverslips were saturated with 2% BSA and 10% normal goat serum (NGS) for 3 h followed by incubation overnight at 4°C in a humidified chamber with primary antibodies. Unbound antibody was removed by three washes and bound antibody was detected by incubation with donkey-anti-rabbit-488 IgG and donkey anti-mouse-555 IgG from Jackson ImmunoResearch (1:300), at room temperature for 30 min. Nuclei were stained with nuclear marker 4,6-diamidino-2-phenylindole dihydrochloride (DAPI; Sigma, St. Louis, MO, United States) 1:1000 in PBS for 5 min and samples were mounted on glass slides and coverslipped with antifade medium. Negative controls were performed by omitting either the primary or the secondary antibody. Images (60×) are representative of at least three independent experiments and were acquired with spinning disk system for fast fluorescence confocal microscopy, with led or laser light source – Crest Optics, (Crisel Instruments, Rome, Italy). Olympus Confocal Microscope Quantitative image analysis were performed by using Metamorph Research Imaging and ImageJ 1.4 software^1^.

Quantification of fluorescence images (from 10 different fields for a total of at least 50 neurons) was performed using ImageJ software. Fixed cells within an experiment were simultaneously stained and imaged with identical settings (exposure time and fluorescence light intensity were kept constant throughout acquisition). Selection of ROIs to acquire fluorescent images was performed either on the axonal or somal marker to avoid bias acquisition. A fixed threshold over the background was applied.

### Protein Cellular Lysates Preparation

Total proteins from primary neuronal cultures were extracted by scraping the septo-hippocampal cells in ice-cold RIPA buffer (50 mM Tris-HCl pH 8, 150 mM NaCl, 1% NP40, 0.1% SDS, 5% sodium deoxycholate plus proteases inhibitor cocktail (Sigma-Aldrich, P8340) and phosphatase inhibitor cocktail (Sigma-Aldrich, P5726/P2850) for 30 min and centrifuged at 4°C for 20 min at 13,000 rpm. The amount of total protein was determined by Bradford assay (Protein Assay Dye Reagent Concentrate, Bio-Rad, Hercules, CA, United States).

### Cell Lysis for Ubiquitin Blots

Ubiquitin analysis requires the use of deubiquitinase (DUB) inhibitor(s) (Gupta et al., 2017). Lysis without these inhibitors runs the risk of degradation of the substrates to be targeted. As such, *N*-ethylmaleimide (Sigma-Aldrich), a potent DUB inhibitor, was used in the lysis buffer. The lysis buffer was 1% (v/v) protease inhibitor cocktail, 1% (v/v) PMSF, 1% (v/v) leucine, and 50 μM NEM in 1% RIPA buffer solution. The amount of total protein was determined by Bradford assay (Protein Assay Dye Reagent Concentrate, Bio-Rad, Hercules, CA, United States).

### Crude Synaptosomal Fractionation

Synaptosome-enriched subcellular fractions -which are largely enriched in both pre- and postsynaptic proteins such as PSD95 and GluR2/3 (Cho et al., 1992; Wemmie et al., 2002)- were prepared from brain hippocampi of WT and Tg2576 mice according to Amadoro et al. (2010). In brief, after a rapid remotion of the brains and dissection of hippocampi, tissues were homogenized in 2 ml of homogenization buffer (320 mM sucrose/4 mM Hepes, pH 7.4/1 mM EGTA/0.4 mM PMSF/plus proteases inhibitor cocktail (Sigma-Aldrich P8340) and phosphatase inhibitor cocktail (Sigma-Aldrich, Oakville, ON, Canada P5726/P2850) with 15 strokes of a glass Dounce tissue grinder (Wheaton). The homogenate was centrifuged at 1,000 × *g* for 10 min. The supernatant was collected and centrifuged at 12,000 × *g* for 15 min, and the second pellet was resuspended in 2 ml of homogenization buffer and centrifuged at 13,000 × *g* for 15 min. The resulting pellet was resuspended in 0.3 ml of homogenization buffer. The amount of total protein was determined by Bradford assay (Protein Assay Dye Reagent Concentrate, Bio-Rad, Hercules, CA, United States).

### SDS-PAGE, Western Blot Analysis, and Densitometry

Equal amounts of protein were separated by SDS-PAGE in 4–12% Bis-Tris gels (Invitrogen), transferred to nitrocellulose membranes (0.45 μm, GE healthcare, Little Chalfont, United Kingdom) and blocked in PBS-T containing 5% non-fat dried milk for 1 h at room temperature. The nitrocellulose filters with 0.1 μM pores followed by cross-linking treatment with PFA 0.4% were used to increase the retention of low-molecular-weight monoubiquitin after transfer and before its detection (Emmerich and Cohen, 2015). Proteins were visualized using appropriate primary antibodies. All primary antibodies were diluted in PBS-T and incubated with the nitrocellulose membrane overnight at 4°C. Incubation with secondary peroxidase coupled anti-mouse or anti-rabbit antibodies was performed by using the ECL system (Thermo Scientific SuperSignal West Pico, 34080; Amersham ECL Prime, RPN2232). Protein loading was monitored by normalization to β-actin. The films were digitized using a professional scanner and quantitative densitometric analysis (expressed in arbitrary units normalized on the expression of the housekeeping protein β-actin) was performed by using ImageJ (see footnote 1).

### Detection of Polyubiquitinated Synapsin I, SNAP25, and α-Synuclein in Primary Septo-Hippocampal Neurons

For polyubiquitinated protein pulldown, proteins (synapsin I, SNAP25, and α-synuclein) were enriched using a kit (Ubiquitin Enrichment Kit, Thermo Scientific), according to the manufacturer’s instructions followed by Western blot. The polyubiquitin Affinity Resin binds polymers of ubiquitin containing four or more ubiquitin subunits while monoubiquitinated proteins and short chain polymers (<4 ubiquitin monomers) are recovered in the flow-through. Proteins were extracted from primary septal neuronal cultures by homogenization in RIPA buffer (supplemented with 0.1 mM PMSF, 1 mM β-glycerophosphate, 1 mM sodium orthovanadate, plus proteases and phosphatase inhibitor cocktail). Briefly, 300 μg of extracted proteins were incubated overnight with 20 μl of resin at 4°C on an end-over-end rotator. The next day the column was centrifuged to eliminate the flow-through and washed three times (10-min intervals each) in the wash buffer (20 mM Tris-HCl, pH 8.0, 150 mM NaCl, 0.1% Tween-20), and bound proteins were eluted with 75 μl SDS sample buffer and then subjected to SDS-PAGE. After boiling and a brief centrifugation, the eluate containing the ubiquitin-enriched fraction was separated by SDS-PAGE in 4–12% Bis-Tris gels (Invitrogen). To aid the transfer of higher molecular weight proteins, the gels were incubated with gel soaking buffer (63 mM Tris-HCl, pH 6.8, 2.3% SDS, 5.0% β-mercaptoethanol) for 30 min. After transfer, the membranes were analyzed by Western blotting by probing with anti-synapsin I, SNAP25 and α-synuclein antibodies to visualize high molecular weight protein(s) poly-ubiquitin conjugates and with an anti-pan ubiquitin antibody as control. The films were digitized using a professional scanner and quantitative densitometric analysis was performed by using ImageJ (see footnote 1).

### Assessment of Chymotrypsin-Like 20S Proteasome Activity

Most cells express the 26S proteasome, which is composed of a constitutive 20S (20S) catalytic core protease, capped by the 19S regulatory complex at each end. Constitutively expressed mammalian 20S proteasomes have three active subunits, beta1, beta 2, and beta 5, possessing post-glutamyl peptide hydrolase-like (PGPH) (i.e., caspase-like, trypsin-like, and chymotrypsin-like activities, respectively). These subunits are responsible for cleaving proteins into short, 3–22 amino acid long, polypeptides (Navon and Ciechanover, 2009).

To measure chymotrypsin-like 20S proteasome activity in cultured septo-hippocampal neurons, cells were plated at a density of 2 × 10^6^ cells onto poly-l-lysine coated 35 mm dishes and maintained in B27-supplemented Neurobasal medium for 10 days. After a time course of NGF deprivation, the cells were collected in cold PBS by scraping from dishes and centrifuged at 4°C for 10 min at 1,200 rpm. The pellets were washed twice in cold PBS, lysed in 0.5% NP40 for 30 min on ice and centrifuged at 4°C for 15 min at 13,000 rpm. The supernatant was collected and used to measure the proteasome activity with a Proteasome Activity Assay Kit (Abcam, Cambridge, United Kingdom) according to the manufactures’ protocol. The assay was based on detection of the fluorophore 7-amino-4-methylcoumarin (AMC) after cleavage from the labeled substrate. 20 μg total protein was incubated in the provided buffer with fluorophore-labeled peptide substrate (LLVY-7-amino-4-methylcoumarin [AMC]) for 120 min at 37°C. The free AMC fluorescence was measured using Ex/Em = 350/340 nm filter set in a microplate reader fluorometer (Victor) in the presence/absence of MG132 inhibitor (10 μM) after 15 min at 37°C for 30–60 min. Assay buffer without lysate served as blank. The activity was linear with respect to the amount of protein (in the range of 20–200 μg of protein). Lysates of cultures treated with MG132 showed <5% of the activity. Samples were assayed in quadruplicate. Data was calculated by plotting AMC standard curve serial dilutions and normalized by the protein concentrations as mean relative fluorescence units (RFU) (350/340 nm)/μg of total proteins and pmol AMC/μg of total proteins ± SEM (three replicates per experiment) and was representative of three independent experiments.

### Electrophysiological Recordings

Whole cell patch-clamp recordings were performed from 10- to 12-day-old *in vitro* (D.I.V.) primary septum neurons to study the excitatory neurotransmission under different culture conditions (Caioli et al., 2011). The recording pipettes were pulled from borosilicate glass with an outer diameter of 1.2 mm and had open tip resistances of 3–5 MΩ. The internal solution for filling pipettes consisted of (in mM): 140 CsCl, 1 EGTA, 10 HEPES, 6 D-glucose (pH 7.4 with CsOH). The standard extracellular solution consisted of (in mM): 130 NaCl, 3 KCl, 2 MgCl_2_, 1.5 CaCl_2_, 10 HEPES, 6 D-glucose, and 10 tetraethyl-ammonium (TEA) Cl (pH 7.4 with NaOH).

To isolate the miniature excitatory post synaptic currents (mEPSCs), 0.5 μM tetrodotoxin (TTX), 5 μM strychnine, 100 μM picrotoxin were added in the bath solution, in order to block voltage-dependent Na^+^ currents, glycine and GABA_A_ receptors, respectively. Recordings were carried out for 5 min from each neuron and the last 2 min of each recording were analyzed.

In order to evaluate the effect of treatment with MG132 (10 μM) and LDN (2.5 μM), drugs were added for 6 h to the culture medium of primary cholinergic neurons immediately after NGF withdrawal.

Experiments were performed at room temperature (22–24°C). Recordings were made using a MultiClamp 700B amplifier (Axon CNS, Molecular Device). pCLAMP 9.2 software was utilized for the data acquisition system (Axon Instruments). The whole cell capacitance was assessed online using the Membrane test function of pClamp9.2. Current signals were sampled at 100 kHz and filtered at 3 kHz.

### Data Analysis

Tissues from at least *n* = 3 animals per experimental group were analyzed. All experiments using primary neurons were performed at least three times independently, each in triplicate. Data was expressed as mean ± SEM. Statistically significant differences were calculated by unpaired independent (two-tailed) *t*-Student’s test for two-groups comparison and by one-way analysis of variance (ANOVA) followed by Bonferroni *post hoc* correction for multiple comparison among more than two groups, as indicated in the figure legends. *p* < 0.05 was accepted as statistically significant (^∗^*p* < 0.05; ^∗∗^*p* < 0.01; ^∗∗∗^*p* < 0.0001). Analysis of biochemical results were performed by GraphPad Prism 6 software. For electrophysiological recordings, the 6.0.7 version of Mini Analysis Program (Synaptosoft Inc., Decatur, GA, United States) was used to analyze mEPSCs. mEPSCs were manually detected using an 8 pA threshold crossing algorithm. Frequency, event amplitude, kinetic characteristics (rise and decay time), and event area were compared between the different experimental conditions. Fitting and statistical analysis were performed using SPSS 17.0.0 for Windows (SPSS Inc., Chicago, IL, United States) and Origin 7.0 (Microcal Software, Northampton, MA, United States).

## Results

### The Ubiquitin-Proteasome System (UPS) Is Early Activated by NGF Withdrawal in Cholinergic Septo-Hippocampal Neurons

It has been reported that local catabolism of only a few presynaptic proteins is mediated by 26S UPS (Speese et al., 2003; Yi and Ehlers, 2005; Segref and Hoppe, 2009; Bingol and Sheng, 2011; Franco et al., 2011; Na et al., 2012) and that the retrograde NGF/TrkA signaling is able to control the synapse(s) assembly independently of its ability to support *de novo* gene expression (Sharma et al., 2010). We have previously shown that NGF withdrawal induces *in vitro* an early, selective and reversible structural and functional deterioration of cholinergic presynaptic terminals, just resembling the “dying-back”-like mechanism(s) of cell degeneration occurring *in vivo* into basal forebrain circuit at the onset of AD neuropathology. In NGF-responsive septal cholinergic-enriched primary neurons, the short-term (-1 h up to -6 h) removal of neurotrophin causes a rapid failure in excitatory neurotransmission which is causally correlated with a concomitant and progressive loss in three specific presynaptic vesicle-trafficking proteins, such as synapsin I, SNAP25 and α-synuclein (Latina et al., 2017).

**FIGURE 1:**
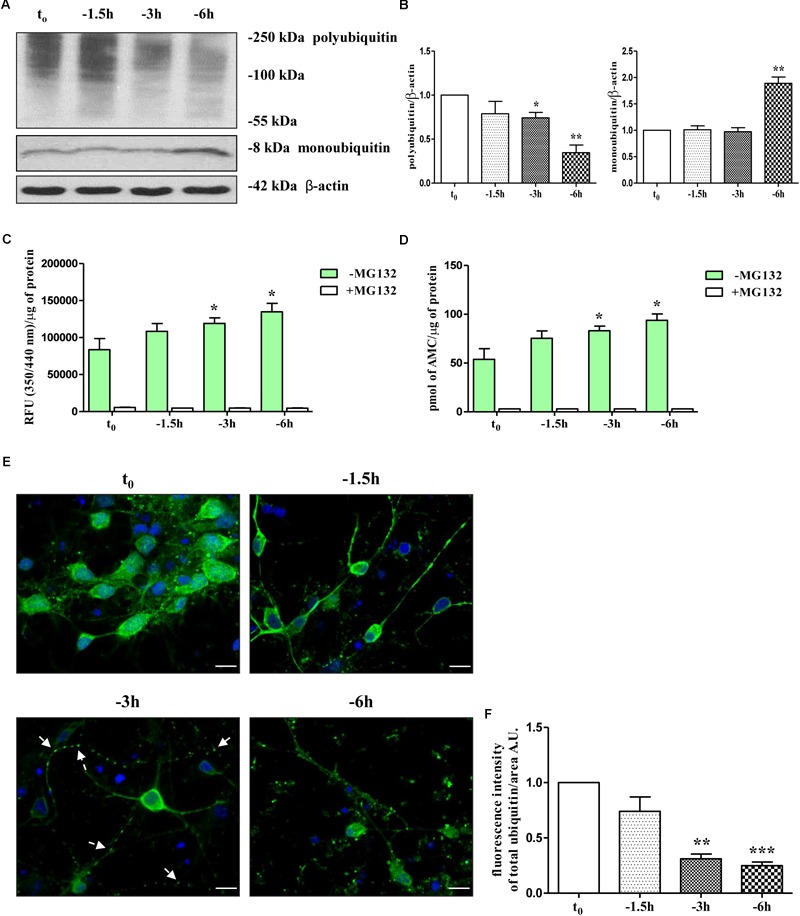
UPS activity is early stimulated by NGF starvation in septo-hippocampal primary neurons. **(A,B)** Western blotting analysis was carried out on equal amounts of total protein extract (20 μg) from septal primary neurons cultured for 10–12 D.I.V. in defined medium supplemented with 0.2% B27, in the presence (*t*_0_) or absence (–1.5, –3, –6 h) of exogenously added NGF (100 ng/ml). By probing with anti-pan ubiquitin antibody (Dako antibody Z0458), immunoblots showed the reduction of polyubiquitinated protein conjugates and, inversely, a parallel increase in the free monoubiquitin which are indicative of an accelerated UPS degradative function(s). Molecular weights are indicated on the right of the blots and expressed in kDa. Densitometric quantification of immunoreactivity levels was normalized by calculating the ratio of the intensity of HMW-smeared signal and monoubiquitin to that of β-actin which was used as loading control for each sample/lane. Values are mean ± standard error of the mean (SEM) of at least five independent experiments and are expressed with respect to control neurons (*t*_0_) at the respective experimental time-points (–1.5, –3, –6 h). Statistically significant differences were calculated by unpaired-two tailed *t*-Student’s test (^∗^*p* < 0.05, ^∗∗^*p* < 0.01). **(C,D)** Septal cholinergic-enriched cultures grown continuously in 0.2% B27 media in the presence of exogenous NGF (100 ng/ml) from plating were deprived of their trophic support for different periods of time (–1.5, –3, –6 h) and the chymotrypsin-like activity was evaluated by utilizing a Succ-LLVY-AMC-tagged substrate peptide which releases free, highly fluorescent AMC in the presence of proteolytic activity of UPS. The kinetics of fluorescence development at Ex/Em = 350/340 nm were measured in the presence/absence of MG132 proteasomal inhibitor which significantly suppressed proteasome activity at all analyzed time points. Data was calculated by plotting AMC standard curve serial dilutions and expressed as mean relative fluorescence units (RFU) (350/340 nm)/μg of total proteins and pmol AMC/μg of total proteins ± SEM (three replicates per experiment). Higher R.F.U. values indicated higher 20S proteasome activity. Statistically significant differences were calculated by unpaired-two tailed *t*-Student’s test (^∗^*p* < 0.05 vs. control (*t*_0_). **(E,F)** Confocal microscopy analysis of immunofluorescence was carried out on septo-hippocampal primary neurons cultured for 10–12 D.I.V. in defined medium supplemented with 0.2% B27 in the presence or absence of exogenously added NGF (100 ng/ml). Staining with anti-pan ubiquitin antibody (Dako antibody Z0458) (green channel) enabled to visualize the intracellular expression levels and the cellular distribution of ubiquitin in septal primary cultures undergoing NGF deprivation for different periods of time (–1.5, –3, –6 h). Nuclei (blue channel) were counterstained with DAPI. The cell bodies appeared round with healthy nuclei whereas axons started to degenerated distally following NGF starvation (“dying-back” phenomenon). Notice the general loss of ubiquitin immunolabeling and the shift from a more diffuse cytoplasmic/nuclear distribution to a more punctate neuritic pattern under the experimental conditions of prolonged NGF withdrawal. Arrows indicated signs of axonal degeneration (beading, varicosities) visible in NGF-deprived cultures. Graph represents the averaged fluorescence intensity ± SEM (n > 100 cells) of total ubiquitin-positive structures per 60 × image-field arbitrary unit (A.U.). Statistically significant differences were calculated by unpaired-two tailed *t*-Student’s test [^∗∗^*p* < 0.01 and ^∗∗∗^*p* < 0.0001 vs. control (*t*_0_)]. Images were representative of at least three independent experiments. Scale bar: 10 μM.

Therefore, in view of these findings and with the intent of exploring the molecular mechanism(s) whereby NGF starvation negatively impacts on the presynaptic maintenance and function(s) in this *in vitro* cholinergic system, the catalytic activity of the UPS and the distribution of ubiquitin-conjugated protein forms were investigated by means of biochemical and morphological assays. To this aim, cultures grown continuously (10–12 D.I.V.) in 0.2% B27 media in the presence of exogenous NGF (100 ng/ml) from plating (*t*_0_) were deprived of this trophic support for different periods of time (-1.5, -3, -6 h) and Western blotting was carried out on whole-cell lysates by probing with anti-pan ubiquitin antibody (Z0458 Dako) (Emmerich and Cohen, 2015). This polyclonal antibody is reported to detect both conjugated-ubiquitin and free ubiquitin (Myeku and Figueiredo-Pereira, 2011; Noor et al., 2013; Emmerich and Cohen, 2015) with similar affinity for Lys48- and Lys63-ubiquitinated substrates (Myeku and Figueiredo-Pereira, 2011). Following short-term removal of neurotrophin, we detected a progressive decrease in immunoreactivity of polyubiquitin chains-conjugates (Figures 1A,B) whose degradation -especially of those formed with ubiquitin lysine 48 (K48) linkage- generally relies on the 26S proteasome (Thrower et al., 2000; Grice and Nathan, 2016) and is canonically used as an indirect cellular indicator of activation in proteasomal function(s) (Myeku et al., 2011). We also found out that significant loss of ubiquitin conjugated was inversely correlated with a parallel accumulation of free ubiquitin up to -6 h of neurotrophin withdrawal which is reminiscent of increased proteasomal degradative activity (Emmerich and Cohen, 2015) (Figures 1A,B and Supplementary Figure S1A). Importantly, densitometric quantification revealed that the median intensity of Ub-positive HMW-smeared signal significantly declined starting from 3 h (0.74 ± 0.061 arbitrary units A.U.); ^∗^*p* < 0.05 *t*-Student’s test versus untreated control *t*_0_) up to 6 h (0.34 ± 0.087 A.U. ^∗∗^*p* < 0.01 *t*-Student’s test versus *t*_0_) of NGF starvation in concomitant with progressive increase of immunoreactivity signal of monoubiquitin (^∗∗^*p* < 0.01 *t*-Student’s test versus *t*_0_) and within a frame-time which completely matched the concomitant and selective downregulation in the steady-state levels of presynaptic synapsin I, SNAP25 and α-synuclein (Latina et al., 2017).

Having established that the reduction in ubiquitin-conjugated proteins suggestive of a proteasomal stimulation was temporally correlated with the early loss of selected presynaptic markers evoked by NGF withdrawal, in order to examine more directly the effect of NGF removal on intracellular UPS-mediated degradation, we set out to monitor for a duration of 1.5, 3, and 6 h the chymotrypsin-like proteolytic activity of 20S subunit catalytic core, which is the main and most active proteasomal activity (Heinemeyer et al., 1991; Orlowski and Wilk, 2000). An efficient and sensitive fluorimetric assay was carried out on crude protein extracts from control and NGF-deprived cultures, in absence or in the presence of MG132, a potent, reversible and cell-permeant proteasomal inhibitor (Buac et al., 2013) included as positive control. The evaluation of proteasomal activity was performed by measuring the emission of aminomethylcoumarin (AMC) which was released after cleavage from the labeled succinyl-Leu-Leu-Val-Tyr-AMC (suc-LLVY-AMC) fluorogenic substrate. As shown in Figures 1C,D and in line with our Western blotting data (Figures 1A,B), the proteasomal degradative function(s) in extracts from NGF-deprived septal neurons increased in a time-dependent manner, peaking after 6 h of neurotrophin removal at value of 61.5% (93.83 ± 6.53 pmol AMC/μg of total proteins; ^∗^*p* < 0.05 *t*-Student’s test versus *t*_0_) higher than in untreated controls (57.76 ± 10.96 pmol AMC/μg of total proteins). Pre-treatment of primary cultures with MG132 (10 μM), completely abrogated the signal of the fluorophore AMC, clearly, thus indicating that the intracellular enzymatic activity experimentally assessed was specific and sensitive to proteasomal inhibition.

Finally, to examine whether NGF deprivation could also alter the subcellular distribution of ubiquitination pattern, immunofluorescence confocal microscopy studies followed by quantitative analysis were carried out in septo-hippocampal cultures from 1.5 to 6 h of neurotrophin removal. Interestingly, in control neurons (*t*_0_) the ubiquitin-immunoreactivity was strongly detectable with diffuse staining throughout the cytoplasm and nuclei whereas neuritic processes appeared only slightly labeled or even negative (Figures 1E,F and Supplementary Figure S1B). After 3 h of NGF withdrawal, a general reduction of the ubiquitin-immunopositive signal was evident in starving neurons along with a robust redistribution of its intensity from perikarya toward the proximal and distal neurite segments which appeared dystrophic, swollen and decorated with regular varicosities (“beads-on-string”) (arrows). In particular, the ubiquitin-positive labeling was no longer evenly distributed in the cell bodies but instead a more punctate staining -mainly localized to the periphery- was clearly detectable away from somatic compartment. This pathological *in vitro* feature resembles the classical “dying-back” axonopathy of the earliest stages of UPS-dependent Wallerian degeneration occurring in NGF-deprived peripheral sympathetic neurons (Zhai et al., 2003; Korhonen and Lindholm, 2004; MacInnis and Campenot, 2005). Moreover, after 6 h of NGF-removal, the density of ubiquitin-conjugated structures was greatly reduced (approximately -70% ^∗∗∗^*p* < 0.0001 *t*-Student’s test versus *t*_0_) and a more pronounced punctate, dot-like staining was clearly appreciable throughout the entire culture.

Taken together, these findings strongly demonstrate that stimulation of UPS early takes place in cholinergic septo-hippocampal primary neurons following NGF withdrawal over a short period of time window (-1 h up to -6 h) overlapping the loss of selected presynaptic vesicle trafficking markers, such as synapsin I, SNAP25 and α-synuclein.

### Pharmacological Suppression of UPS Activity Attenuates in a Dose-Dependent Manner the NGF-Dependent Reduction of Presynaptic Synapsin I, SNAP25, and α-Synuclein

**FIGURE 2:**
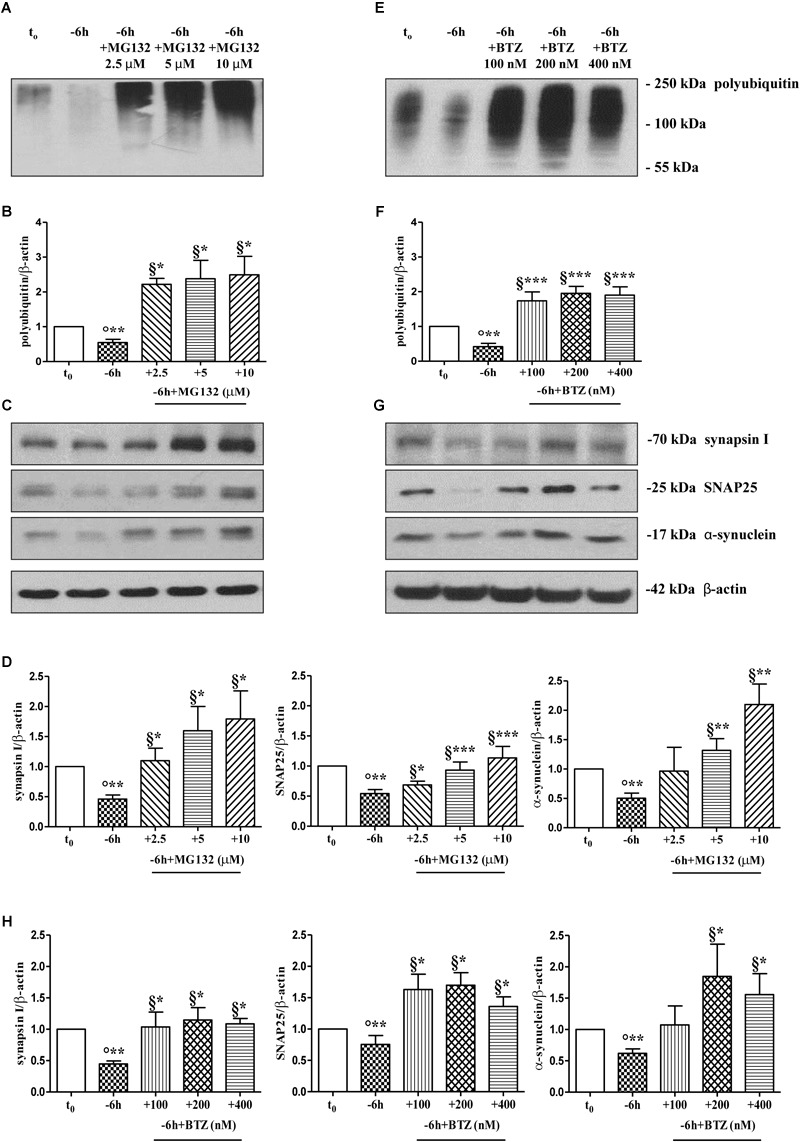
Pharmacological blockage of UPS by MG132 and BTZ, two well-established selective and cell-permeable proteasomal inhibitors, prevents the decline in the steady-state levels of presynaptic markers synapsin I, SNAP25, and α-synuclein in NGF-deprived septo-hippocampal primary neurons. **(A–H)** Representative blots of detergent lysates from septal cholinergic-enriched cultures which were grown continuously (10–12 D.I.V.) in 0.2% B27 media in the presence of exogenous NGF (100 ng/ml) from plating (*t*_0_) and then deprived of their trophic support for 6 h (–6 h) in the absence or presence of increasing concentration of MG132 (2.5, 5, 10 μM) and BTZ (100, 200, 400 nM) inhibitors. At time points indicated, cells were collected and equal amounts of total protein extracts (20 μg) were analyzed by SDS-PAGE by probing with anti-pan ubiquitin antibody (Dako antibody Z0458) (A-E for MG132 and BTZ, respectively) and specific antibodies against the indicated presynaptic markers (C-G for MG132 and BTZ, respectively). Densitometric quantification of immunoreactivity levels of ubiquitin (B-F for MG132 and BTZ, respectively) and synapsin I, SNAP25 and α-synuclein (D-H for MG132 and BTZ, respectively) was calculated by normalizing the signals of each band to corresponding β-actin intensities on the same blots. Values were mean ± SEM of at least of five independent experiments and were expressed with respect to control neurons (*t*_0_). Statistically significant differences were calculated at the respective experimental points by unpaired-two tailed *t*-Student’s test [^∗^*p* < 0.05, ^∗∗^*p* < 0.01 and ^∗∗∗^*p* < 0.0001 vs. *t*_0_ control neurons (°) and vs. –6 h NGF deprivation (§)].

To further explore the causal role of UPS in *in vitro* NGF-induced “dying-back” mechanism(s) of presynaptic terminals pruning, we set out to assess the effect of proteasomal inhibition on the steady-state levels of presynaptic synapsin I, SNAP25 and α-synuclein upon short-term neurotrophin removal. To this aim, septo-hippocampal cultures grown continuously (10–12 D.I.V.) in 0.2% B27 media in the presence of exogenous NGF (100 ng/ml) from plating (*t*_0_) were deprived of this trophic support for 6 h, in the absence or presence of increasing doses of MG132 inhibitor (2.5, 5, 10 μM). Western blotting analysis was performed on total protein RIPA-buffer extracts from treated and control cultures by probing both with anti-pan ubiquitin antibody (Z0458 Dako) and with specific antibodies against the three presynaptic markers. As shown in Figures 2C,D, acute proteasomal inhibition in 6 h NGF-deprived septal neurons was able to significantly attenuate the reduction in the expression levels of synapsin I, SNAP25 and α-synuclein in a dose-dependent way (^∗^*p* < 0.05, ^∗∗^*p* < 0.01, ^∗∗∗^*p* < 0.0001 *t*-Student’s test versus *t* = -6 h) leading to the appearance of several low-molecular-weight ubiquitinated species only following higher time expositions of the blots. The concomitant and global accumulation of HMW polyubiquitinated conjugates (Figures 2A,B) confirmed the efficacy of the treatment with MG132, in line with previous findings (Leitch et al., 2001). Due to the evidence that MG132 is endowed with reactivity not only toward the active proteasomal subunits (β1, β2, and β5) but also against different types of proteases, including serine proteases and calpain (Myung et al., 2001), we further validated these results by incubation of cultures with bortezomib (BTZ) a more potent, specific and not-reversible proteasome-blocking drug (Buac et al., 2013). As shown in Figures 2G,H, treatment of cholinergic neurons undergoing 6 h of NGF withdrawal with increasing doses of BTZ (100, 200, 400 nM) yielded results comparable with those obtained with MG132, in agreement with inhibitor(s) ability in promoting the accumulation of HMW-smeared immunoreactivity signal (Figures 2E,F). Nonetheless, the strongest stabilizing effect of BTZ on the intracellular turnover of synapsin I (1.147 ± 0.198 A.U.), SNAP25 (1.699 ± 0.199 A.U.) and α-synuclein (1.84 ± 0.51 A.U.) in comparison to -6 h untreated cultures (0.6 ± 0.08 A.U.) was obtained at 200 nM concentration (^∗^*p* < 0.05, *t*-Student’s test versus *t* = -6 h) because the highest concentration (400 nM) turned out to be less effective. This effect appeared to be specific for proteasome inhibitors because incubation of cultures with Z-VAD-fmk caspase inhibitor or pepstatin A serine protease inhibitor were ineffective (Supplementary Figures S2A,B). No significant change on the basal levels of synapsin I, SNAP25 and α-synuclein was detected in control neurons upon incubation with proteasome inhibitors alone (data not shown). Likewise, under the same experimental conditions, the protein expression of other presynaptic and/or post-synaptic markers -such as syntaxin I, synaptophysin and NR1 that we previously showed not to be changed by NGF withdrawal in this *in vitro* neuronal model (Latina et al., 2017)- were contextually unaffected by MG132 and BTZ administration (Supplementary Figure S2C). This evidence is in agreement with the findings that metabolic turnover of most synaptic proteins does not rely on the UPS but likely follows alternative intracellular pathways of degradation (Hakim et al., 2016). Furthermore, treatment of neuronal cultures with MG132 and BTZ were not *per se* neurotoxic, as assessed by MTT assay and quantification of DAPI-positive nuclear staining (Supplementary Figures S2D,E), consistent with previous report showing that >16 h of exposure to proteasomal inhibitors is required to induce a noticeable *in vitro* cell death in primary neurons (Snider et al., 2002). Importantly, short term (-6 h) incubation with MG132 and BTZ did not significantly change the levels of free monoubiquitin (Supplementary Figures S3A,B) indicating that their inhibitory effects were directly mediated on proteolysis and not by indirect action on ubiquitination of target proteins. This is in line with the previous findings referring that only under long period of incubation time (>16 h) with proteasome inhibitors, ubiquitin is not recycled and the pool of monoubiquitin available for ubiquitination is reduced (Patnaik et al., 2000; López et al., 2011).

**FIGURE 3:**
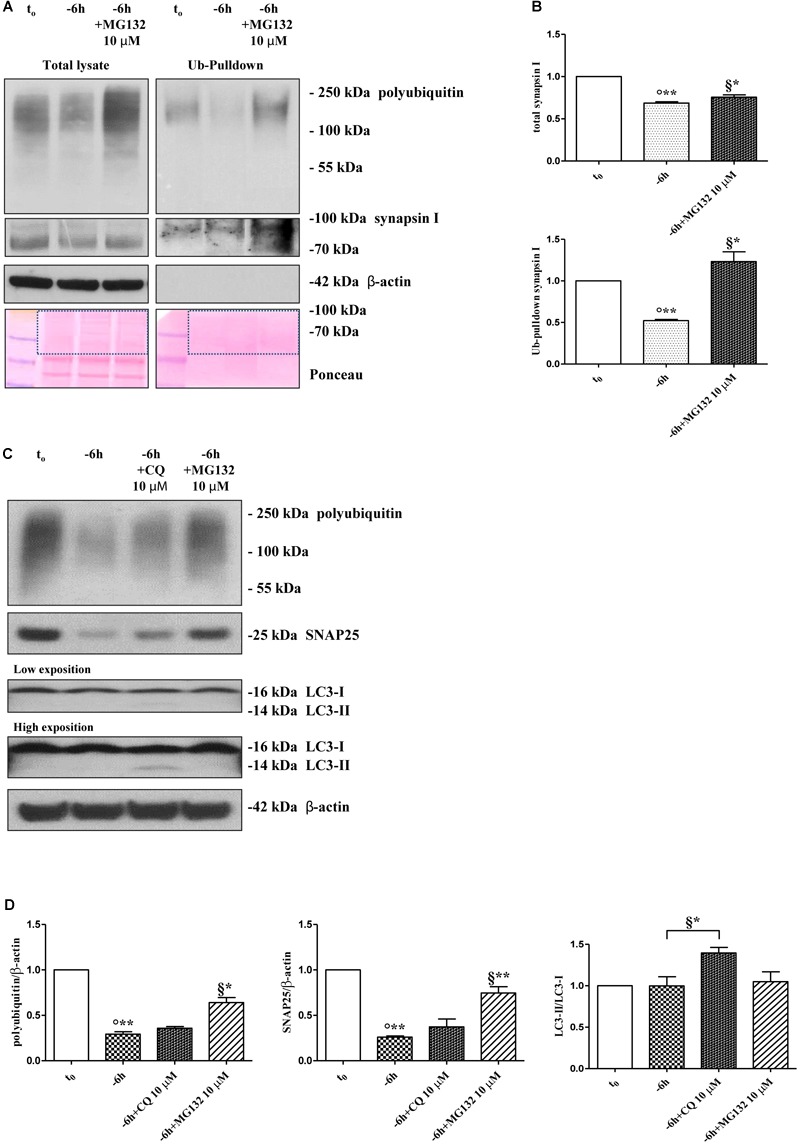
Inhibition of UPS -but not of autophagy- protects the presynaptic terminals of septo-hippocampal neurons from NGF withdrawal. **(A,B)** The intracellular levels of synapsin I-polyubiquitin conjugates were determined in control (*t*_0_) and 6 h NGF-deprived (–6 h) cultures in the absence or presence of MG132 (10 μM), following polyUb-pull down of total protein extracts by ubiquitin affinity beads and immunoblotting with anti-synapsin I and anti- pan ubiquitin (Dako antibody Z0458) antibodies. Ponceau staining was used to ascertain the equal protein loading of gels. Dotted boxes indicate the area used for quantification **(B)** Notice that β-actin signal was not recovered after column elution of polyUb-conjugates. Values were mean ± SEM of three independent experiments and were expressed with respect to control neurons (*t*_0_). Statistically significant differences were calculated at the respective experimental points by unpaired-two tailed *t*-Student’s test [^∗^*p* < 0.05, ^∗∗^*p* < 0.01 vs. *t*_0_ control neurons (°) and vs. –6 h NGF deprivation (§)]. **(C,D)** Septal cholinergic-enriched cultures were grown continuously (10–12 D.I.V.) in 0.2% B27 media in the presence of exogenous NGF (100 ng/ml) from plating (*t*_0_) and then deprived of their trophic support for 6 h (–6 h) in the absence or presence of CQ (10 μM) and MG132 (10 μM). Cell lysates were extracted and analyzed by Western blotting by probing for ubiquitin (Dako antibody Z0458), SNAP25, LC3 and β-actin. Representative blot showing the effect of drugs treatment on the intracellular levels of neuronal proteins **(C)** and relative densitometric quantification **(D)** were shown. Data were reported as mean ± SEM and expressed ratio of the value from control neurons (*t*_0_) Statistically significant differences were calculated at the respective experimental points by unpaired-two tailed *t*-Student’s test [^∗^*p* < 0.05, ^∗∗^*p* < 0.01 vs. *t*_0_ control neurons (°) and vs. –6 h NGF deprivation (§)]. Only the inhibition of proteasomal activity by MG132 led to significant accumulation of polyubiquitin-conjugates and increase in the expression levels of SNAP25 in 6 h NGF-deprived septo-hippocampal neurons. The increase in LC3-II/LC3-I ratio confirmed the efficacy of CQ treatment.

Protein ubiquitination is not necessarily indicative of proteasomal degradation because monoubiquitination regulates the protein trafficking, involving endosomes, as well as other important cellular functions (Hicke, 2001). Thus, to ascertain that the three presynaptic proteins were actually polyubiquitinated in a NGF-dependent manner, we performed a pulldown analysis of polyubiquitinated-conjugates on whole-cell proteins lysates from septo-hippocampal cultures undergoing 6 h of neurotrophin withdrawal, in the absence or presence of MG132 inhibitor (10 μM). This assay protocol relies on a commercially available affinity resin (Thermo Scientific) which allows for the isolation of polyubiquitinated proteins whereas the monoubiquitinated species, or short chain polymers (<4 ubiquitin monomers), are removed being recovered in the flow-through. As shown in Figures 3A,B, after polyUb-enrichment partitioning and Western blotting analysis with anti-pan ubiquitin (Z0458 Dako) and anti-synapsin I antibodies, we found that the immunoreactivity of affinity-purified synapsin I declined in neuronal cultures following 6 h NGF starvation and that its levels dramatically increased upon pharmacological proteasomal blockade in a MG132-reversible manner. Importantly, this trend virtually mirrored the pattern detected in not-fractionated whole-cell lysates, demonstrating that NGF withdrawal actually stimulates the polyubiquitination of synapsin I and its degradation by the UPS under genuine cellular conditions. Moreover, after the fractionation procedure, we contextually detected a clear electrophoretic mobility shift in the signal of the heterogeneous HMW-smeared signal which plainly proved that the polyubiquitinated-enriched proteins were successfully recovered after affinity-based column elution, having an added mass of at least 32 kDa (corresponding to a chain of 4 × 8 kDa monomers of ubiquitin) compared to the mass of the non-modified ones from total lysates. Similar results were found for α-synuclein and SNAP25 (data not shown).

A neuronal cross-talk between the ubiquitin-proteasome and autophagy-lysosome systems takes place intracellularly and the types of ubiquitin linkages are known to influence the final protein fate with polyubiquitylation (K48- and K-63-linked chains, respectively) which targets substrates for proteolysis along both degradative pathways (Korolchuk et al., 2010). In order to distinguish between these two alternative but not mutually exclusive possibilities, we investigated the effect of pharmacological treatment with chloroquine (CQ), an alkalizing reagent used to raise the lysosomal pH leading to inhibition of fusion of autophagosome with lysosome and, then, of lysosomal protein degradation. In contrast to the robust action of MG132 proteasomal inhibitor on the stability of presynaptic marker(s) (^∗∗^*p* < 0.01, *t*-Student’s test versus *t* = -6 h), incubation of NGF-deprived septal neurons with 10 μM CQ failed to attenuate the NGF-induced downregulation in the steady-state levels of SNAP25 (*p* = 0.2762 versus *t* = -6 h) (Figures 3C,D). The efficacy of CQ treatment in blocking the autophagy-mediated degradation was confirmed by the increased conversion ratio LC3II/LC3I which is frequently used to monitor the endogenous autophagic flux in neurons (Kabeya et al., 2000; Klionsky et al., 2008, 2012; Viscomi et al., 2012). In addition, the weak and not-significant (*p* = 0.0934 versus *t* = -6 h) increase in immunoreactivity of polyubiquitin-conjugates detected by Dako anti-pan ubiquitin antibody after treatment with CQ indicates that, in this *in vitro* model, the K63-polyubiquitin linkages -which specifically target proteins to the endosomal–lysosomal system (Grice and Nathan, 2016)- contributed less than K48-polyubiquitin chains, which are the major signal for UPS-mediated degradation (Grice and Nathan, 2016), in controlling the intracellular protein turnover. On the other hand, in line with previous observations (Myeku and Figueiredo-Pereira, 2011), the minimal accumulation of polyubiquitinated proteins detected after CQ treatment was more likely to be due to weak proteasomal inhibition by the lysosomotropic agent (or by impaired flux through the UPS owing to substrate excess) because autophagy inhibition fails to elevate ubiquitin chains unless the UPS is affected. Finally, similar results were found for the other two analyzed presynaptic markers, synapsin I and α-synuclein (Supplementary Figures S4A,B).

Taken together, these *in vitro* results indicate that synapsin I, SNAP25 and α-synuclein are actually polyubiquitinated and degraded by UPS in septo-hippocampal primary neurons in response to short-term (-6 h) NGF withdrawal.

### The Activity of Ubiquitin C-Terminal Hydrolase L-1 (UCHL-1) Is Required for the Downregulation of Presynaptic Markers in NGF-Deprived Cholinergic Septo-Hippocampal Neurons

**FIGURE 4:**
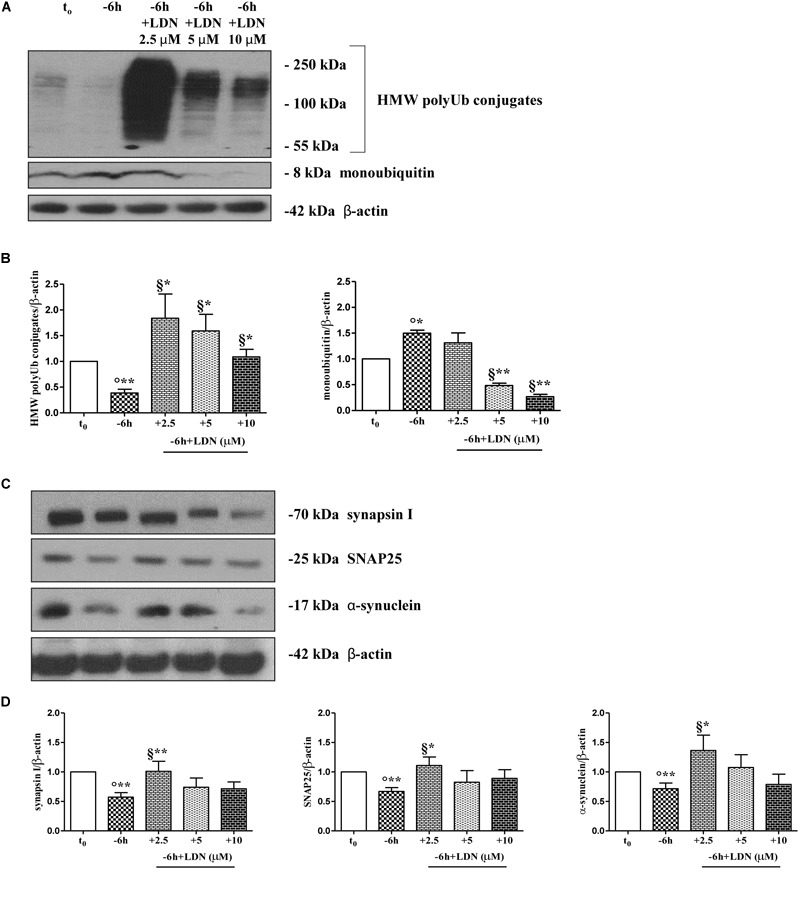
UCHL-1 activity is required to the NGF-dependent control of the synapsin I, SNAP25 and α-synuclein stability in septo-hippocampal primary neurons. **(A–D)** Septal cholinergic-enriched cultures were grown continuously (10–12 D.I.V.) in 0.2% B27 media in the presence of exogenous NGF (100 ng/ml) from plating (*t*_0_) and then deprived of their trophic support for 6 h (–6 h) in the absence or presence of increasing concentration of LDN (2.5, 5, 10 μM), a selective and cell-permeable UCHL-1 inhibitor. Representative Western Blotting of sample extracts analyzed for the expression levels of poly-/monoubiquitin **(A)**, synapsin I, SNAP25 and α-synuclein **(C)** were shown. Cropped image was shown in **(A)**. Bar graphs **(B–D)** showed the densitometric quantification of immunoreactivity levels normalized by calculating the ratio of the intensity of the signal for the protein of interest to that of β-actin which was used as loading control for each sample/lane. Values were mean ± SEM of at least of five independent experiments and were expressed with respect to control neurons (*t*_0_). Statistically significant differences were calculated by unpaired-two tailed *t*-Student’s test [^∗^*p* < 0.05, ^∗∗^*p* < 0.01 vs. *t*_0_ control neurons (°) and vs. –6 h NGF deprivation (§)].

**FIGURE 5:**
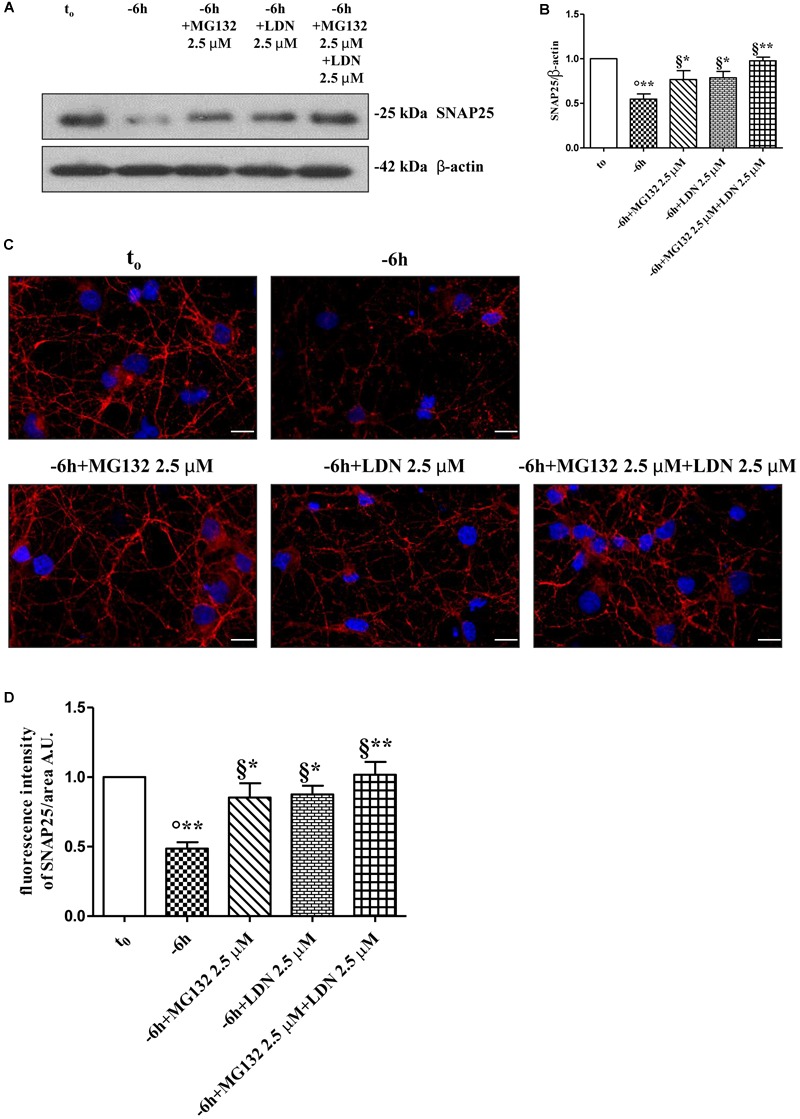
Co-treatment with MG132 and LDN has additive effect in preventing the presynaptic degeneration occurring in –6 h NGF-deprived cholinergic neurons. **(A–D)** Septal cholinergic-enriched cultures were grown continuously (10–12 D.I.V.) in 0.2% B27 media in the presence of exogenous NGF (100 ng/ml) from plating (*t*_0_) and then deprived of their trophic support for 6 h (–6 h) in the absence or presence of MG132 (2.5 μM), LDN (2.5 μM) and MG132 (2.5 μM)+LDN (2.5 μM) combination. Levels of SNAP25 and β-actin were assessed by Western blotting analysis **(A)** and densitometric quantification **(B)** was calculated as ratio of the value from control neurons (*t*_0_) and reported as mean ± SEM. Statistically significant differences were calculated at the respective experimental points by unpaired-two tailed *t*-Student’s test [^∗^*p* < 0.05, ^∗∗^*p* < 0.01 vs. *t*_0_ control neurons (°) and vs. –6 h NGF deprivation (§)]. Representative immunofluorescence images **(C)** and bar graph **(D)** showing the SNAP25 expression levels in cholinergic primary neurons following NGF withdrawal and inhibitors treatments were reported. Cultures were fixed, permeabilized and immunostained for SNAP25 (red channel) and nuclei were counterstained with DAPI (blue channel). Quantification of SNAP25-positive structures per 60 × image-field arbitrary unit (A.U.) was calculated as ratio of the value from control neurons (*t*_0_) and reported as mean ± SEM. Statistically significant differences were calculated at the respective experimental points by unpaired-two tailed *t*-Student’s test [^∗^*p* < 0.05, ^∗∗^*p* < 0.01 vs. *t*_0_ control neurons (°) and vs. –6 h NGF deprivation (§)]. Images were representative of at least three independent experiments. Scale bar: 10 μM.

Dynamic ubiquitylation is governed by the antagonistic intracellular actions of ubiquitin ligases and DUBs. Among the DUBs, UCHL-1 is the most abundant, neuron-specific ubiquitin C-terminal hydrolase which regulates the structure and function(s) of synaptic terminals by controlling the local ubiquitin dynamics (Cartier et al., 2009). Relevantly, UCHL-1 changes are involved in the pathogenesis of AD (Pasinetti, 2001; Choi et al., 2004; Gong et al., 2006). UCHL-1 is endowed with both hydrolase activity, by removing ubiquitin molecules from the degraded proteins in order to be reused for *de novo* cycles of ubiquitination (Finley et al., 1989; Larsen et al., 1998), and with ubiquityl ligase activity, by linking ubiquitin molecules and generating polyubiquitin chains that tag the target proteins for UPS-mediated elimination (Liu et al., 2002). Therefore, to ascertain whether the UCHL-1 activity contributed, directly or indirectly, to the UPS-mediated clearance of selected presynaptic proteins, we evaluated the effects of changes in UCHL-1 dependent dynamics of the total (i.e., bound and monomeric/unconjugated) ubiquitin pool on the turnover of synapsin I, SNAP25 and α-synuclein under *in vitro* conditions of NGF starvation. To this aim, cultures grown continuously (10–12 D.I.V.) in 0.2% B27 media in the presence of exogenous NGF (100 ng/ml) from plating (*t*_0_) were deprived of this trophic support for 6 h (-6 h) in the absence or presence of increasing doses (2.5, 5, 10 μM) of LDN, an inhibitor which selectively halts UCHL-1 without any effect on other UCH family members (Liu et al., 2003; Gong et al., 2006; Cartier et al., 2009). Western blotting on whole-cell lysates were carried out to check the protein expression of synapsin I, SNAP25 and α-synuclein, the pattern of polyubiquitinated-conjugates and the stability in the intracellular pool of free/monomeric ubiquitin. Pharmacological suppression of UCHL-1 (Figures 4C,D) with the lowest used concentration of LDN (2.5 μM) was effective in significantly rescuing the NGF-dependent drop in synapsin I (^∗∗^*p* < 0.01 *t*-Student’s test versus *t* = -6 h), SNAP25 (^∗^*p* < 0.05, *t*-Student’s test versus *t* = -6 h) and α-synuclein immunoreactivity (^∗^*p* < 0.05, *t*-Student’s test versus *t* = -6 h), in line with drug ability (Figures 4A,B) of inducing a strong elevation in the Ub-positive HMW-smeared signal (^∗^*p* < 0.05, *t*-Student’s test versus *t* = -6 h). Conversely, treatment with higher experimental doses of LDN (5, 10 μM) (Figures 5C,D) turned out to be unsuccessful in elevating the intracellular levels of synapsin I (*p* = 0.126, *p* = 0.671 *t*-Student’s test versus *t* = -6 h, respectively), SNAP25 (*p* = 0.857, *p* = 0.363 *t*-Student’s test versus *t* = -6 h, respectively) and α-synuclein (*p* = 0.205, *p* = 0.423 *t*-Student’s test versus *t* = -6 h, respectively) consistent with their more potent effect in lessening the free monomeric ubiquitin (^∗∗^*p* < 0.01 *t*-Student’s test versus *t* = -6 h) than in causing the concomitant accumulation of polyubiquitin conjugates (^∗^*p* < 0.05, *t*-Student’s test versus *t* = -6 h) (Figures 4C,D). These results are in agreement with previous findings showing that the homeostasis of unconjugated ubiquitin is *per se* crucial in maintaining the synapse structure and function(s) (Chen et al., 2011) and that synaptic-enriched UCHL-1 is able to associate with and inhibit the intracellular degradation of its monomeric free pool (Osaka et al., 2003). Furthermore, Western blotting analysis followed by densitometric quantification indicated that there was a trend for an increase of the intracellular abundance of UCHL1 in neuronal cultures during the time-dependent NGF removal (Supplementary Figures S5A,B). Finally, the co-incubation of -6 h NGF-deprived cholinergic neurons with lowest effective subtoxic concentrations of MG132 (2.5 μM) and LDN (2.5 μM) displayed an additive effect in preventing the decline in SNAP25 immunoreactivity (^∗∗^*p* < 0.01 *t*-Student’s test versus *t* = -6 h), as shown in Western blotting analysis on total proteins extracts from co-treated cultures (Figures 5A,B). Consistent with our biochemical results, immunocytochemistry staining (Figures 5C,D) also showed a significant increase in the density of SNAP25- positive puncta in (MG132+LDN)-exposed cultures in comparison with its 6 h NGF-deprived counterpart (^∗∗^*p* < 0.01 *t*-Student’s test versus *t* = -6 h). Similar results were found for the other two presynaptic proteins of interest, synapsin I and α-synuclein (Supplementary Figures S6A,B).

These *in vitro* findings indicate that: (i) UCHL-1 is early involved in the “dying-back”-type degeneration occurring in septo-hippocampal primary neurons following alterations in NGF/TrkA signaling pathway and that its moderate inhibition is effective in blocking the selective loss of α-synuclein, SNAP25 and synapsin I; (ii) NGF withdrawal orchestrates in this *in vitro* model an early (-6 h) UPS-mediated destruction of these presynaptic markers by means of balanced fine-tuning in the dynamic UPS-mediated ubiquitylation/degradation and UCHL-1-dependent (mono)ubiquitin turnover.

### The UPS Activity and UCHL-1-Dependent (Mono)ubiquitin Homeostasis Support the NGF-Dependent Modulation of Excitatory Neurotransmission in Cholinergic Septo-Hippocampal Neurons

Nerve growth factor exerts a potent and specific stimulatory presynaptic action on cholinergic nerve terminals (Wu and Yeh, 2005; Huh et al., 2008) by increasing the frequency and not the amplitude of spontaneous mEPSCs. In this context, we have previously shown that the short-term (-6 h) interruption in NGF/TrkA signaling pathway in cholinergic septo-hippocampal primary neurons decreases the averaged frequency of mEPSCs by downregulating the steady-state levels of synapsin I, SNAP25, and α-synuclein (Latina et al., 2017) which critically control the vesicular trafficking and neurotransmission at presynaptic terminals.

**FIGURE 6:**
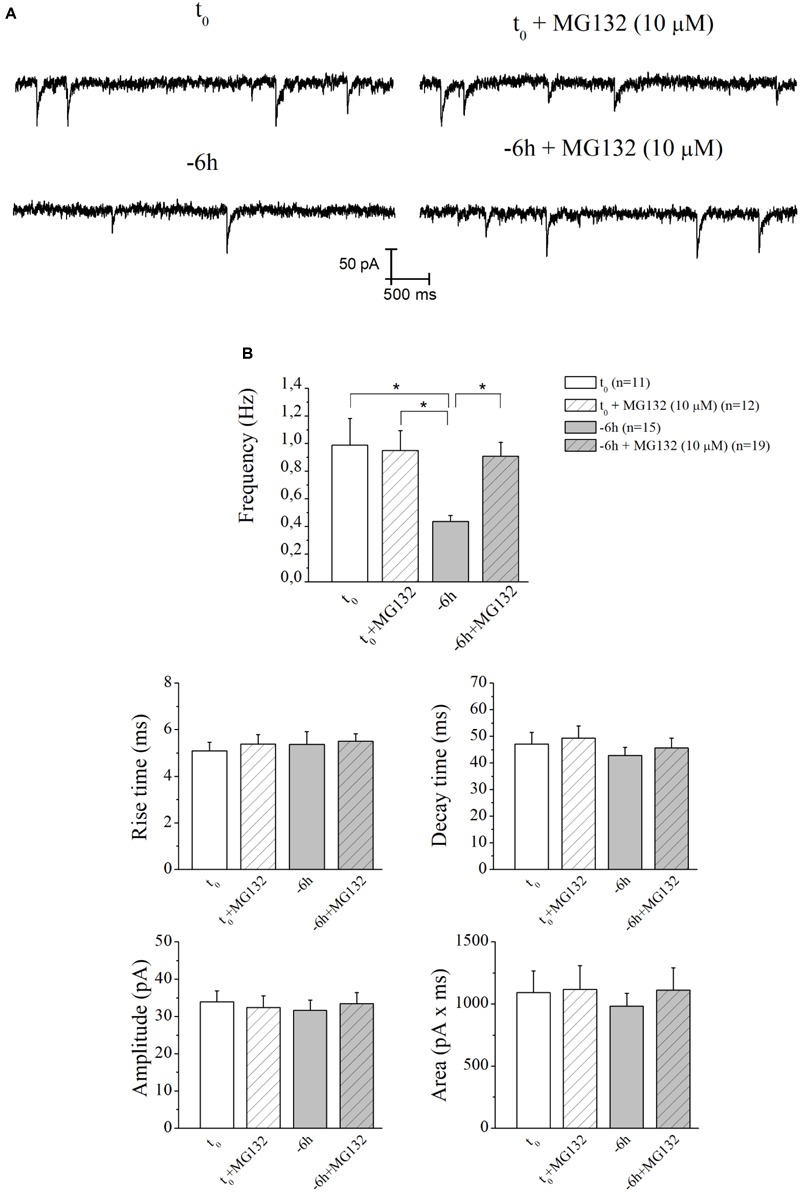
UPS mediates the *in vitro* modulatory action of NGF on presynaptic function of cholinergic septo-hippocampal primary neurons. **(A)** Representative traces of septal neurons grown for 10–12 D.I.V. in B27 0.2%+NGF, deprived for 6 h of NGF, in the absence and presence of MG132 UPS-inhibitor (10 μM) were shown. **(B)** Bar plots reported the mean ± SEM of frequency, amplitude, rise time, decay time and area of the mEPSCs recorded in neuronal populations under different experimental conditions. The reduction in mEPSCs frequency detected in *in vitro* cholinergic-enriched neurons following 6 h NGF withdrawal (–6 h: 0.43 ± 0.04 Hz, *n* = 15) in comparison with B27 0.2%+NGF control ones (*t*_0_: 0.99 ± 0.19 Hz, *n* = 11) was significantly rescued by incubation with MG132 UPS inhibitor (–6 h+MG132: 0.9 ± 0.1 Hz, *n* = 19). No significant change was detected in control neurons upon incubation with MG132 inhibitor (*t*_0_+MG132: 0.95 ± 0.14 Hz, *n* = 12). Values were mean ± SEM of at least four independent cultures and statistically significant differences were calculated by one-way ANOVA followed by Bonferroni’s correction (^∗^*p* ≤ 0.02). In line with data reporting a specific effect of this neurotrophin on *in vitro* cholinergic neurons at the presynaptic level (Wu and Yeh, 2005; Huh et al., 2008; Latina et al., 2017), no significant differences were detected for the mean amplitude, as well as the mean area and the kinetic parameters, of the mEPSCs recorded from cultures in all analyzed experimental conditions (*p* > 0.05), neither by NGF deprivation alone nor by incubation of MG132.

Therefore, having demonstrated an early (-6 h) and causal role of UPS-degradation/ubiquitination rate in controlling the NGF-dependent turnover of these three presynaptic markers, we further explored the physiologic relevance of acute perturbations of UPS activity in modulating the excitatory neurotransmission in this *in vitro* paradigm. To this aim, electrophysiological recordings of mEPSCs -which are known to rise from random release of presynaptic vesicles at nerve endings and to play important roles in maintaining functional connections of synaptic terminals (Caioli et al., 2011; Saba et al., 2016)- were carried out on cholinergic primary neurons undergoing short-term removal of exogenous NGF for 6 h (-6 h), in the presence or absence of MG132 (10 μM) and LDN (2.5 μM). As shown in Figures 6A,B, the mean of the mEPSCs frequency recorded at the holding potential -60 mV was significantly lower in -6 h NGF-deprived neurons (-6 h: 0.44 ± 0.04 Hz; *n* = 15) than in control ones (*t*_0:_ 0.99 ± 0.19 Hz; *n* = 11; *p* ≤ 0.02), in keeping with our previous findings (Latina et al., 2017). Interestingly, acute inhibition of UPS by MG132 treatment (10 μM) was fully able to rescue the excitatory neurotransmission because the mEPSCs frequency of -6 h NGF-deprived neurons+MG132 was significantly higher than of -6 h untreated cultures (-6 h+MG132: 0.91 ± 0.1 Hz; *n* = 19; *p* ≤ 0.02 versus *-*6 h) by approximating the *t*_0_ control values (*p* > 0.05 versus *t*_0_). These electrophysiological data tightly correlated with Western blotting analysis (Figure 2) showing the ability of MG132 in selectively preventing the NGF-induced downregulation of synapsin I, SNAP25, and α-synuclein, the three well-established presynaptic crucially involved in neurotransmitter exocytosis at nerve endings. On the contrary, no significant effect was detected in the mEPSCs frequency when MG132 was added to cultures in basal conditions (*t*_0_+MG132: 0.95 ± 0.14 Hz; *n* = 12; *p* > 0.05 versus *t*_0_). Likewise, in line with previous data reporting a specific effect of this neurotrophin on *in vitro* cholinergic neurons at the presynaptic level (Wu and Yeh, 2005; Huh et al., 2008; Latina et al., 2017), the mean amplitude as well as the mean area and the kinetic parameters of the mEPSCs recorded from cultures were not modified in any experimental condition (*p* > 0.05) neither by NGF deprivation alone nor by incubation of MG132.

**FIGURE 7:**
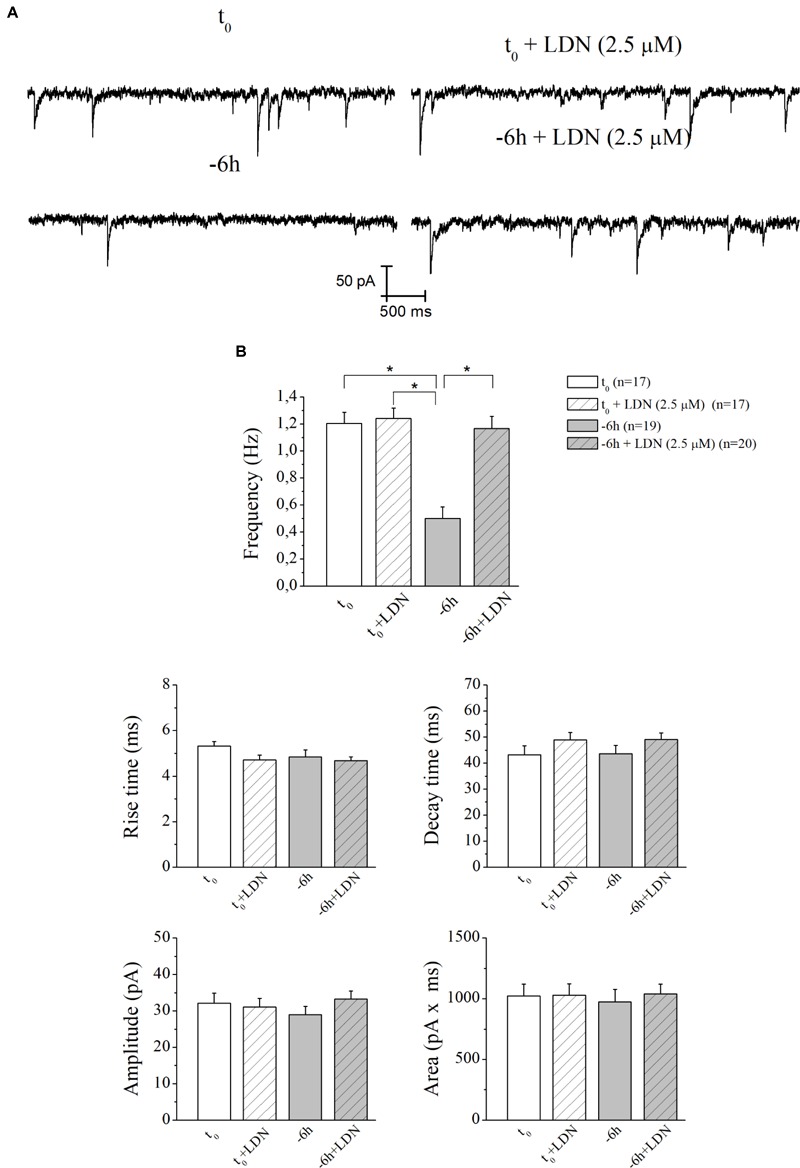
The decay in mEPSCs frequency of NGF-deprived cultures is also recovered by LDN treatment. **(A)** Representative traces of septal neurons grown for 10–12 D.I.V. in B27 0.2%+NGF, deprived for 6 h of NGF, in the absence and presence of LDN UCHL-1 inhibitor (2.5 mM) were shown. **(B)** Bar plots reported the mean ± SEM of frequency, amplitude, rise time, decay time and area of the mEPSCs recorded in neuronal populations under different experimental conditions. The reduction in mEPSCs frequency detected in *in vitro* cholinergic-enriched neurons following 6 h NGF withdrawal (–6 h: 0.5 ± 0.08 Hz, n = 19) in comparison with B27 0.2%+NGF control ones (*t*_0_: 1.2 ± 0.08 Hz, *n* = 17) was significantly rescued by incubation with LDN an UCHL-1 inhibitor (–6 h+LDN: 1.16 ± 0.09 Hz, *n* = 20). No significant change was detected in control neurons upon incubation with LDN inhibitor (*t*_0_+LDN: 1.24 ± 0.07 Hz, *n* = 17). Values were mean ± SEM of at least four independent cultures and statistically significant differences were calculated by one-way ANOVA followed by Bonferroni’s correction (^∗^*p* < 0.001). No significant differences were found for the mean amplitude, as well as the mean area and the kinetic parameters, of the mEPSCs recorded from cultures in all analyzed experimental conditions (*p* > 0.05), neither by NGF deprivation alone nor by incubation of LDN.

Similar results (Figures 7A,B) were found after incubation of -6 h NGF-deprived septal cultures with UCHL-1 inhibitor LDN (2.5 μM) (-6 h+LDN: 1.16 ± 0.09 Hz; *n* = 20; *p* < 0.001 versus *-*6 h, *p* > 0.05 versus *t*_0_; *t*_0_+LDN: 1.24 ± 0.07 Hz; *n* = 17; *p* > 0.05 versus *t*_0_).

These results highlight the pivotal role of UPS and UCHL-1-dependent (mono)ubiquitin dynamics in mediating the NGF-induced modulatory actions on the presynaptic morphology and neurosecretory function(s) in *in vitro* cholinergic septal primary neurons.

### Early Decline in Ubiquitination Pattern and Loss of Specific Presynaptic Proteins Also Occur *in vivo*, in Hippocampal Synaptoneurosomes From Aging AD Transgenic Mouse Model Tg2576 (HuAPP695 SWE), in Association With Selective Down-Regulation of Cholinergic Markers

Reduction of ChAT- labeled nerve endings, deficiency in cholinergic transmission and spatial learning/memory ability along with downregulation in expression of NGF and its cognate receptors have been detected in aging Tg2576 animals (Apelt et al., 2002; Chauhan and Siegel, 2003; Klingner et al., 2003; Lüth et al., 2003; Simmons et al., 2014; Zhu et al., 2017, a transgenic AD mouse line carrying the mutated form of APP named AβPPSWE (K670N/M671L). In this well-established AD paradigm, memory deficits are just evident at 3 months of age and are progressive (Pompl et al., 1999; King and Arendash, 2002; Kobayashi and Chen, 2005) although insoluble Aβ aggregates start to raise from 6 months (Kawarabayashi et al., 2001) and Aβ-laden plaque deposition is frankly detectable only at 11 months (Hsiao et al., 1996; Irizarry et al., 1997).

**FIGURE 8:**
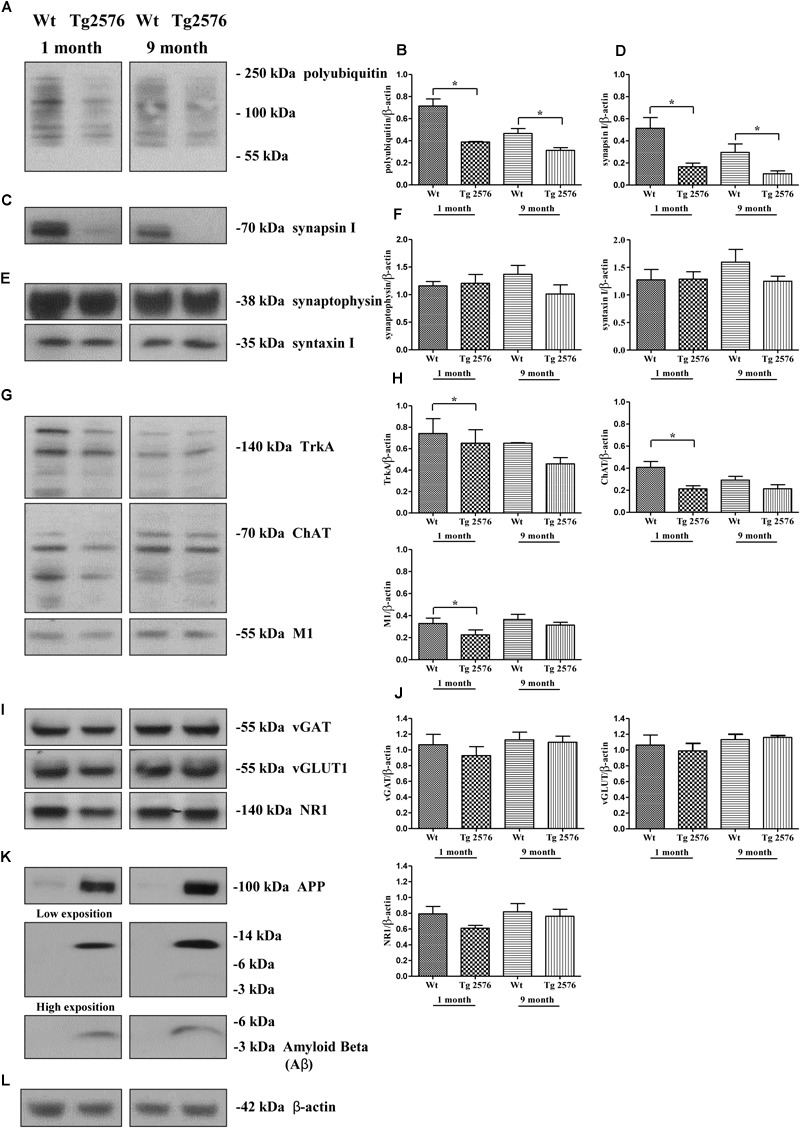
Early and selective degeneration of cholinergic afferent inputs is paralleled by the decline of presynaptic markers and loss in polyubiquitin-conjugates in hippocampi from transgenic Tg2576 AD mice, just mirroring the *in vitro* “dying-back”-like mechanism(s) of NGF-deprived cholinergic neurons. **(A–L)** Crude synaptosomal preparations of hippocampi representing mainly the presynaptic compartment (Gylys et al., 2004) were isolated from 1-month-old and 9-months-old Tg2576 AD mice and age-matched littermate Wt (*n* = 4–6 pooled mice lysates/experimental group) and analyzed by Western blotting for the expression levels of ubiquitin **(A)**, synapsin I **(C)**, synaptophysin and syntaxin I **(E)**, TrkA, ChAT and M1 as cholinergic markers **(G)**, vGAT, vGLUT1 and NR1 as non-cholinergic markers **(I)**, β-amyloid monomer/oligomeric species (anti β-amyloid 1-16 antibody, clone 6E10), APP holoprotein (Anti-APP 66-81 antibody, clone 22C11) **(K)**. Bar graphs **(B,D,F,H,J)** showed the densitometric quantification of immunoreactivity levels normalized by calculating the ratio of the intensity of the signal for the protein of interest to that of β-actin **(L)** which was used as loading control for each sample/lane. Values were mean ± SEM of at least of five independent experiments and were expressed with respect to corresponding age-matched wild-type counterpart. Statistically significant differences were calculated by unpaired-two tailed *t*-Student’s test (^∗^*p* < 0.05). Notice that glutamatergic and GABAergic neurotransmission were unaffected in this AD animal model despite the increasing aging of mice and the progressive accumulation of soluble Aβ monomeric/oligomeric species. On the contrary, the selective denervation of more vulnerable cholinergic neuronal afferents was just evident in young 1-month-old Tg2576 AD mice when compared to their age-matched littermate wild-type controls.

In order to strengthen the pathological relevance of our *in vitro* findings in AD neurodegeneration, regional synaptosomes were isolated from young/presymptomatic (1-month-old) and middle-aged (9-months-old) Tg2576 mice and WT control counterparts. Since basal forebrain neurons are the main source of cholinergic innervation to the cortex and hippocampus *in vivo* (Niewiadomska et al., 2011), hippocampal pinched-off nerve terminals – which are known to represent mainly the presynaptic compartment obtained by fractionated brain homogenates (Gylys et al., 2004) – were evaluated for the abundance of synapsin I, SNAP25 and α-synuclein, the temporal profile of ubiquitination and cholinergic markers expression. As shown in Figures 8C,D, the steady-state levels of synapsin I was significantly reduced in aging AD animals from both experimental groups (0.1662 ± 0.032 A.U. from 1-month-old Tg2576 mice; 0.1017 ± 0.027 A.U. from 9-months-old Tg2576 mice) in comparison with their age-matched non-transgenic littermates (0.5136 ± 0.096 A.U. from 1-month-old WT mice; 0.2952 ± 0.076 A.U. from 9-months-old WT mice, ^∗^*p* < 0.05, *t*-Student’s test). A similar negative trend was found for SNAP25 and α-synuclein (data not shown). Relevantly and in line with the evidence that vesicle trafficking proteins are not equally affected in human AD brains (Sze et al., 2000; Honer, 2003; Reddy et al., 2005), no change was contextually detected up to 9 months in the expression rates of syntaxin-I (*p* = 0.5296 and 0.3838 for 1- and 9-months-old group versus age-matched WT mice, respectively) and synaptophysin (*p* = 0.4839 and *p* = 0.1149 for 1- and 9-months-old group versus age-matched WT mice, respectively) (Figures 8E,F), two other presynaptic markers we reported to be unaffected by NGF withdrawal in *in vitro* cholinergic-enriched septo-hippocampal primary cultures (Latina et al., 2017).

Interestingly and in agreement with our *in vitro* results (Figure 2), the synaptic distribution of the age-related HMW polyubiquitin protein conjugates also declined over time in AD-affected isolated hippocampal nerve terminals, in both 1- and 9-months-old Tg2576 animals (0.3884 ± 0.005 A.U. and 0.3133 ± 0.024 A.U., respectively ^∗^*p* < 0.05, *t*-Student’s test) related to experimental age-matched control groups (0.7142 ± 0.064 A.U. and 0.4667 ± 0.044 A.U: for 1- and 9-months-old WT mice, respectively) (Figures 8A,B). Furthermore, the immunoreactivity signals from three different well-confirmed cholinergic-specific markers (Dawbarn et al., 1988; Holtzman et al., 1992; Sobreviela et al., 1994), such as M1 (1-month-old group: WT 0.3284 ± 0.048 A.U., Tg2576 0.2251 ± 0.045 A.U.; 9-months-old group: WT 0.3665 ± 0.045 A.U., Tg2576 0.3136 ± 0.026 A.U.), ChAT (1-month-old group: WT 0.4076 ± 0.053, Tg2576 0.2128 ± 0.028; 9-months-old group: WT 0.2921 ± 0.034 A.U., Tg2576 0.2137 ± 0.036 A.U.) and TrkA (1-month-old group: WT 0.742 ± 0.138 A.U., Tg2576 0.651 ± 0.125 A.U.; 9-months-old group: WT 0.651 ± 0.005 A.U., Tg2576 0.458 ± 0.058 A.U.) (Figures 8G,H), were also gradually reduced in AD mice -mainly in symptomatic 1-month-old young animals (Figures 8G,H) (^∗^*p* < 0.05 *t*-Student’s test versus age-matched WT controls)- when compared with corresponding non-transgenic littermate counterparts. Finally and more importantly, in contrast to the prominent loss of cholinergic projections and the progressive accumulation of soluble Aβ monomeric/oligomeric neurotoxic species (Figure 8K), the protein abundance of vGLUT1 (*p* = 0.057 and *p* = 0.5153 for 1- and 9-months-old group versus age-matched WT mice, respectively) NR1 (*p* = 0.0978 and *p* = 0.4822 for 1- and 9-months-old group versus age-matched WT mice, respectively), vGAT (*p* = 0.206 and *p* = 0.6155 for 1- and 9-months-old group versus age-matched WT mice, respectively) turned out to be unmodified in hippocampal transgenic synaptosomes up to 9 months (Figures 8I,J), suggesting that glutamatergic- and GABAergic-specific nerve terminals were not the most susceptible neuronal population in this aging AD mice model.

These results suggest that : (i) the *in vivo* degeneration of cholinergic afferent inputs early occurs in hippocampi from transgenic Tg2576 AD mice in correlation with loss of selective presynaptic markers and with age-related increase in ubiquitin turnover, just mirroring the *in vitro* “dying-back”-like mechanism(s) of presynaptic elimination we previously showed to occur in NGF-responsive cholinergic-enriched primary neurons following neurotrophin starvation (Latina et al., 2017); (ii) these specific correlative pathological changes take place in diseased hippocampi at 1 month of age when soluble Aβ monomeric/oligomeric neurotoxic species and cognitive deficits are already evident in this AD animal model and in the absence of any significant age-dependent alterations in glutamatergic and GABAergic neurotransmission.

## Discussion

In the present paper, we report that NGF/TrkA signaling pathway controls the neurotransmission strength in cholinergic septo-hippocampal primary neurons on rapid timescale (-6 h) via balanced interplay between the UCHL-1-dependent regulation of the (mono)ubiquitin homeostasis and the UPS-mediated degradation of selected presynaptic proteins. We demonstrate that the NGF withdrawal induces *in vitro* an early UPS activation which, in turn, causes a diminution in the expression levels of three vesicle trafficking proteins, such as synapsin I, SNAP25 and α-synuclein, with consequent decrement of the neurotransmission function. Our findings identify for the first time the UPS and ubiquitin metabolism as novel molecular players serving in the NGF-dependent control of architecture and short-term plasticity of cholinergic synapses under physiological and pathological conditions. The clinical relevance of these *in vitro* observations is also confirmed on cholinergic nerve terminals from hippocampi of aging Tg2576 transgenic mice, a well-established AD animal model showing deficits of spatial learning/memory ability in correlation with an early and selective impairment in ACh-based neurotransmission. Our biochemical, morphological and electrophysiological evidence delineate a pivotal role of UPS imbalance and NGF/TrkA system dysfunction in the early structural and functional decay of cholinergic synapses occurring at the prodromal stages of AD neuropathology progression.

### UPS, NGF/TrkA Signaling, and Cholinergic Synapses in AD Neurodegeneration

The UPS has recently emerged as a major regulatory mechanism of synaptic plasticity and neural circuit remodeling in the CNS by the fine-tuning of the half-life of several pre- and/or post-synaptic proteins which crucially subserve the activity-dependent synaptic structural and functional modifications (Bingol and Schuman, 2005, 2006; Patrick, 2006; Yi and Ehlers, 2007; Haas and Broadie, 2008). Compelling data from several animal models have demonstrated a causal connection between UPS-dependent control of turnover of key presynaptic protein(s) and neurotransmitter release (Willeumier et al., 2006; Yao et al., 2007; Jiang et al., 2010; Rinetti and Schweizer, 2010; Lazarevic et al., 2011). For instance, various presynaptic proteins- including synapsin I, SNAP25, and α-synuclein participating in synaptic vesicle exocytosis, endocytosis, and recycling- are known to be ubiquitinated and UPS substrates (Bennett et al., 1999; Webb et al., 2003; Segref and Hoppe, 2009; Bingol and Sheng, 2011; Franco et al., 2011; Na et al., 2012; Hakim et al., 2016) and UPS components are present in presynaptic boutons serving in the synaptic proteostasis and dynamic regulation of neurotransmission (Speese et al., 2003; van Roessel et al., 2004; Willeumier et al., 2006; Hegde and Upadhya, 2007; Jiang et al., 2010; Rinetti and Schweizer, 2010). The ataxia mice axJ, with a loss-of-function mutation in the UPS-associated deubiquitinating enzyme Usp14 show severe defects of the neuromuscular junction with impaired presynaptic function (Wilson et al., 2002; Chen et al., 2009) which are rescued by restoration of intracellular ubiquitin levels (Chen et al., 2011). On the other hand, transgenic mice overexpressing ubiquitin also display impaired formation of presynapses (Hallengren et al., 2013). As a consequence of the pivotal role of UPS in normal maintenance and functioning of synapses, abnormalities in the steady-state levels of cellular ubiquitin pool and/or 26S proteasome activities have been proved to crucially contribute to the onset/progression of numerous human neurodegenerative diseases, including AD, which are causally associated to an early synaptic failure (Upadhya and Hegde, 2007; Tanaka and Matsuda, 2014; Gadhave et al., 2016). *In vitro* and *in vivo* experimental studies have also clearly demonstrated a causal link between human AD neurodegeneration and impaired neurotrophin NGF/TrkA signaling pathway (Counts and Mufson, 2005; Schliebs and Arendt, 2006; Mufson et al., 2008; Cattaneo and Calissano, 2012), highlighting the cholinergic synapses as selectively affected in incipient AD progression (Davies and Maloney, 1976; Perry et al., 1978; Whitehouse et al., 1981; Coyle et al., 1983; Wong et al., 1999; Bell et al., 2006; Hampel et al., 2018). In this context, our data carried out on two well-established cellular and animal AD models of cholinergic deficits, such as NGF-deprived septal primary neurons (Hartikka and Hefti, 1988a,b; Svendsen et al., 1994; Triaca et al., 2016; Canu et al., 2017; Latina et al., 2017) and aging Tg2576 mice (Apelt et al., 2002; Lüth et al., 2003; Laursen et al., 2014; Zhu et al., 2017), strongly support the pathological relevance of reduced availability of target-derived NGF in early disruption of the septo-hippocampal pathway which is known to be associated to cognitive deficits in MCI and with MCI progression toward AD (Mufson et al., 2012). Furthermore and more importantly, given that the local changes in the substrate degradation rate alter the number of synaptic boutons which regulate the strength of synaptic transmission at synaptic terminals (Ding and Shen, 2008; Haas and Broadie, 2008), our biochemical and electrophysiological results may have important translational implications by identifying a novel mechanicistic link between the UPS-mediated degradation of key presynaptic proteins and the neurosecretory function(s) sustained by NGF/TrkA signaling pathway at vulnerable cholinergic nerve endings. By demonstrating a strong, *in vitro* inhibitory effect of UPS and UCHL-1 inhibitor(s) on the NGF-dependent changes in spontaneous excitatory neurotransmission frequency and the expression levels of polyubiquitinated synapsin I, SNAP25 and α-synuclein, our results indicate that this neurotrophin exerts in primary cholinergic cultures a tight control over neurotransmission on the presynaptic site via ubiquitin-dependent, proteasomal-mediated degradation of these three secretory-relevant target substrates.

Concerning the target-selectivity and the timing of NGF-dependent, UPS-mediated presynaptic degradation at cholinergic nerve endings, it’s worth noticing that -although UPS activity is localized in synaptic terminals (Chain et al., 1995; Patrick, 2006) and synaptic activity promotes UPS sequestration within dendritic spines (Bingol and Schuman, 2006)- only few synaptic proteins actually undergo local, activity-regulated and UPS-mediated degradation (Colledge et al., 2003; Ehlers, 2003; Bingol and Schuman, 2005; Jiang et al., 2010; Shin et al., 2012; Alvarez-Castelao and Schuman, 2015). Interestingly, under basal conditions, both pharmacological and genetic suppression of proteasomal activity does not significantly change the constitutive degradation rates in the bulk of resident synaptic proteins up to 10–24 h (Kalla et al., 2006; Shin et al., 2012; Hakim et al., 2016). Consistently, the local, activity-inducible, UPS-mediated degradation at terminal ends appears to be devoted mainly to reshape the synaptic structure/functions within a well-defined and narrow spatio-temporal window (Hanus and Schuman, 2013), while the constitutive clearance of the majority of synaptically residing proteins occurs along different intracellular degradative pathways (Cohen and Ziv, 2017). To this regard it has been demonstrated that alterations in activity/distribution of UPS over minutes to a few hours affect the strength of synaptic transmission (Speese et al., 2003; Zhao et al., 2003), the size of the precycling pool of vesicles (Willeumier et al., 2006) and the LTP (Dong et al., 2008), mainly owing to local changes in the abundance of selected synaptic proteins (Yi and Ehlers, 2005). In this context, by demonstrating that the rapid (up to 6 h) changes in UPS-dependent proteolysis of three crucial presynaptic vesicle-trafficking proteins, such as synapsin I, SNAP25 and α-synuclein, actually mediate the NGF-dependent modulatory function of neurotransmission strength efficacy on fast timescale, our electrophysiological, morphological and biochemical results are more consistent, both in terms of spatial restriction and target selectivity, with the specific physiopathological role of UPS in synapse(s) elimination (Ding et al., 2003; Hegde, 2004; Bingol and Schuman, 2005; Haas and Broadie, 2008) and in regulation of presynaptic neurotransmitter release (Speese et al., 2003; Zhao et al., 2003; Willeumier et al., 2006; Rinetti and Schweizer, 2010), by means of local control in the degradation rate of selected proteins which are crucial for neuronal plasticity (Cline, 2003). Collectively, our studies shed light into the molecular route underlying the early synaptic deterioration induced by alterations of the NGF/TrkA system in the basal forebrain by showing that the initial UPS stimulation followed by degradation of selected presynaptic markers can contribute to AD-associated cholinergic denervation.

### Role of NGF-Dependent Ubiquitin-Mediated Pathways (Mono- Versus Polyubiquitination) in Modulation of Neurosecretory Function(s) of Cholinergic Synapses: Implications in AD Pathogenesis

As ubiquitin synaptic pools are particularly vulnerable to subtle fluctuations in their local stability (Chen et al., 2009), deficiency of ubiquitin homeostasis *per se* critically contributes to many neurodegenerative diseases, including AD, characterized by early synaptic impairment (Chen et al., 2011). Relevantly, changes in UCHL-1 -the most abundant, neuron-specific ubiquitin C-terminal hydrolase which regulates the structure and function(s) of synaptic terminals by controlling the local ubiquitin dynamics (Cartier et al., 2009)- are known to be involved in the pathogenesis of AD (Pasinetti, 2001; Choi et al., 2004; Gong et al., 2006). Furthermore, it has been reported that long-term (24 h) pharmacological inhibition of UCHL-1 with high doses of LDN (10 μM) in primary hippocampal neurons -a neuronal population whose *in vitro* survival does not strictly rely on NGF supply- is able to significantly reduce the protein abundance of monomeric ubiquitin pool and to alter the synaptic morphology, by provoking accumulation and redistribution of two post-synaptic scaffold proteins known to be regulated by ubiquitin-dependent UPS-mediated degradation (Cartier et al., 2009). In addition, no apparent change in UPS activity is found in 10 μM LDN-treated primary neurons, suggesting that UCHL-1 inhibition can indirectly affect the global ubiquitin-dependent UPS degradation, mainly by decreasing the stability of free, unconjugated ubiquitin (Cartier et al., 2009). In line, overexpression of WT UCHL-1 significantly up-regulated the free monomeric ubiquitin expression levels without altering proteasomal activity (Kyratzi et al., 2008).

However, despite intense research efforts, the biological function of UCHL-1 remains enigmatic because it’s still unclear whether the effects observed on monoubiquitin levels are simply due to ubiquitin binding by UCHL-1 or whether hydrolytic and/or ligase activities are also required *in vivo* (Bishop et al., 2016). *In vitro*, LDN affects both the catalytic (ubiquitinating and/or deubiquitinating) and the monoubiquitin binding/stabilizing activities of UCHL-1 at 10 μM (Cartier et al., 2009). However, UCHL-1 activities appear to be independent as showed by the two defective mutants- the C90S and D30A–which both lack hydrolytic activity but the former still maintains its ubiquitin binding ability which is thought to be important for stabilizing ubiquitin levels (Osaka et al., 2003; Sakurai et al., 2008). Furthermore, some of biochemical experiments aimed at evaluating the effect of LDN on UCHL-1 activities were carried out in cell-free system using purified recombinant components (Cartier et al., 2009) that might not be in physiological concentration, and, then, might not faithfully represent genuine cellular events. Finally, monoubiquitin has also a physiological role in UPS-independent, non-degradative pathways which are crucial in controlling the endo/exocytosis recycling and membrane trafficking of presynaptic SNARE synaptic-vesicle proteins, including SNAP25, α-synuclein and synapsin I (Hicke and Dunn, 2003; Yi and Ehlers, 2005).

In this framework, although we did not directly perform *in vitro* measurements of catalytic activities of UPS in NGF-depleted cultures in the absence or presence of increasing concentrations of LDN, we reasoned that short-time (-6 h) incubation with the lowest dose of LDN (2.5 μM) turns out to be the most effective in stabilizing the synapsin I, SNAP25 and α-synuclein because this treatment increases the pool of polyubiquitin conjugates (inhibition of UPS-degradative pathway likely by inhibition of UCHL-1 hydrolytic activity) at greater extent than it does not perturbate the extent of monoubiquitin pool (recycling, not-degradative secretory/trafficking pathway). Conversely, at higher doses of LDN (5, 10 μM) that reduce the extent of monoubiquitin pool more than increasing the pool of polyubiquitin conjugates, it’s reasonable to hypothesize that the synapsin I, SNAP25 and α-synuclein undergoing a finely balanced turnover into presynaptic terminals may be mis-sorted and/or mistargeted away from synapses to cytosolic lysosomes or vacuoles where they are, eventually, degraded (Hicke and Dunn, 2003; Rizzoli, 2014). Under cellular conditions in which the turnover of proteasomal substrates is compromised and the free monoubiquitin is heavily depleted as well, such as following treatment of -6 h NGF-deprived cultures with the highest dose of LDN (10 μM), it’s possible that a general cellular stress response can be activated and autophagy works in clearing the mistargeted/ubiquitinated proteins (Kroemer et al., 2010). In line with these observations we found out that at -6 h NGF deprivation the synergistic and positive effect on the expression levels of these three presynaptic proteins is detected only following co-incubation of cultures with the lowest doses of MG132 (2.5 μM) and LDN (2.5 μM). In contrast, exposure of cultures to CQ alone does not significantly change the stability of these three presynaptic proteins (supporting their degradation mainly by proteasome and not by autophagy. Finally our findings are consistent with other studies referring that: (i) UCHL-1 permits ubiquitinated proteins to access the proteasome and, thus its pharmacological inhibition leads to accumulation of poly-ubiquitinated conjugates, likely by impairing the poly-ubiquitin removal from protein targeted for degradation before entry into the proteasome 20S with consequent indirect effect on the proteasome activity (Saigoh et al., 1999; Costes et al., 2011); (ii) the intracellular storage of ubiquitinated aggregates in patients with lysosomal storage disorders is associated with deficiency of UCHL-1 (Bifsha et al., 2007). Moreover, electrophysiological recordings of the mEPSCs frequency in 6 h NGF-deprived cholinergic primary neurons upon acute treatment with MG132 and LDN (2.5 μM) inhibitors strongly corroborate the finding that the homeostatic regulation of presynaptic terminals neurosecretory functions by NGF exposure relies on both UPS- and UCHL-1-mediated, (mono)ubiquitin-dependent processes. Finally, these results are consistent with the key role of UPS and UCHL-1 as primary effectors of BDNF, another neurotrophin brain-derived neurotrophic factor known to be involved in the regulation of synaptic proteome during the maintenance of LTP (Santos et al., 2015).

In conclusion, our findings show that graded (according to its dosage) and short-time (-6 h) *in vitro* inhibition of UCHL-1 is able in NGF-dependent primary neurons to differently modulate the stability of certain but not all presynaptic proteins, including the three key vesicles secretory/trafficking synapsin I, SNAP25 and α-synuclein. However, the interplay between UCHL-1-dependent ubiquitination/deubiquitination cycles for a substrate and its monoubiquitin-stabilizing action can be quite complex and more work is needed to deeply clarify the different role of ubiquitin (mono- versus polyubiquitination) in modulating the steady-state pools of SNAP-25, α-synuclein and synapsin I in NGF-dependent primary septo-hippocampal neurons and, then, cholinergic neurotransmission.

### Changes in Synaptic UPS Early Contribute to Degeneration of NGF-Dependent Cholinergic Terminals in AD Mouse Model

Cholinergic neuropathology has been largely documented in Tg2576 mice with the loss of cholinergic terminals starting from 5 months of age (Apelt et al., 2002; Klingner et al., 2003; Lüth et al., 2003; Zhu et al., 2017) whereas the decrease in chymotrypsin-like UPS activity (Oh et al., 2005) -leading to the accumulation of ubiquitin-positive conjugates (McMillan et al., 2011)- has been reported in this AD animal model only starting from 10 months. By Western blotting analyses on isolated hippocampal synaptosomes (Figure 8) with specific antibodies against three different well-confirmed cholinergic-specific markers -such as TrkA, ChAT and M1 (Dawbarn et al., 1988; Holtzman et al., 1992; Sobreviela et al., 1994)- and pan-ubiquitin, we demonstrate that cholinergic deafferentation/degeneration is just evident in early-stage Tg2576 mice at 1 month of age when cognitive deficits appear (Pompl et al., 1999; King and Arendash, 2002; Kobayashi and Chen, 2005) and that it occurs in correlation with an increased turnover of polyubiquitin chains-conjugates in comparison with age-matched littermate WT controls, clearly indicating a strong stimulation of proteasomal function(s). Furthermore, although the cholinergic deficits develop along a progressive negative trend which does not reach statistical significance in the 9-months-old transgenic group in comparison with age-matched littermate WT controls, it’s noteworthy that the glutamatergic and GABAergic neurotransmission were contextually unaffected in this AD animal model, despite the increasing animals aging and the time-dependent sequential accumulation of toxic Aβ monomeric/oligomeric species. On the other hand, the sizeable reduction in the cholinergic terminals we detected in middle-stage AD animals (9-months-old) is in contrast with previous study reporting presynaptic cholinergic integrity in whole hippocampus from old (14, 18, and 23 months) Tg2576 mice (Gau et al., 2002). To this regard, differences between experimental procedures (morphometric investigation and colorimetric enzymatic activity versus biochemical analysis) and tissue sampling (synaptoneurosomal fractions versus total brain region homogenates) should be taken into account because the effects observed in entire hippocampus do not be necessarily extended to isolated pinched-off presynaptic terminals representing mainly the actual neurosecretory compartment (Gylys et al., 2004). Furthermore, and in line with the cholinergic hypothesis of AD onset/development which reports that abnormalities in NGF-dependent basal forebrain cholinergic nerve endings are also evident in non-demented elderly people (Geula et al., 2008; Hampel et al., 2018), the evidence that the decline in polyubiquitinated conjugates also takes place in old WT control mice in concomitance with selective dysfunction of cholinergic synapses reveals that age-dependent UPS changes are pathologically relevant mechanisms of cholinergic vulnerability that can early contribute to the onset/progression of AD pathogenesis beyond the amyloidogenesis (Cheng et al., 2018).

### UPS Dysfunction(s) in AD Progression: Clinical Implications

The clinical relevance of altered proteasomal degradation in AD pathology is increasingly recognized but it still remains unclear whether these UPS aberrations are primary or secondary phenomena resulting from other neurodegeneration-occurring causes (de Vrij et al., 2004). Although no literature studies have yet clearly correlated in human brain autoptic tissues the changes in the proteasomal activity and/or the subunit(s) composition with the severity of AD pathology, several evidence favor a situation in which the protein quality control capacity, including the UPS function(s), could be up- or down-regulated or even unchanged during the temporal progression of age-related neurodegeneration in relation with the disease stage. A diminution in proteolytic activities of 20S proteasome by approximately 50% has been detected in crude brain lysates from middle-late stage (Braak stage = 6) AD patient brains (Keller et al., 2000; López Salon et al., 2000; Keck et al., 2003) and in transgenic AD mice models (Oddo et al., 2004; Oh et al., 2005), in line with global decline in UPS system leading to proteostasis failure and sequential accumulation of ubiquitin-tagged misfolded/damaged unremoved proteins during the disease progression (Ciechanover and Kwon, 2015; Gadhave et al., 2016). On the other hand, other authors have found that the chymotrypsin-like and peptidyl-glutamyl peptide-hydrolyzing enzyme (PGPH)-like catalytic activities -which are direct indexes of the UPS degradative function(s) (Heinemeyer et al., 1991; Orlowski and Wilk, 2000)- are significantly enhanced in: (i) brains from hAPP-YAC overexpressing transgenic mice (Seo and Isacson, 2010), in association with a downregulation of NGF levels; (ii) early-middle (Braak stage = 3) patients affected from AD or from Down’s syndrome (DS)/Trisomy 21, with the extra copy of the APP gene located on chromosome 21 (Gillardon et al., 2007; Seo and Isacson, 2010), both suffering memory deficits due to alterations of NGF metabolism, functional elimination of synaptic contacts and extensive loss of central ACh-mediated neuronal functions (Isacson et al., 2002; Chen et al., 2018). Therefore, in keeping with our *in vitro* and *in vivo* data, it’s reasonable to hypothesize that an initial and transient up-regulation of the UPS can occur at early-middle stages of AD neuropathology in vulnerable hippocampal/cortical regions in causal connection with the altered function of NGF-dependent cholinergic projections and the synaptic damage/elimination. During the disease progression and, likely due to the age-dependent increase in oxidative stress and/or to the energetic drop in mitochondrial-derived ATP (Okada et al., 1999; Shringarpure et al., 2000; Huang et al., 2013), a functional decline in UPS sequentially takes place accounting for the progressive accumulation of polyubiquitinylated and/or aggregated toxic proteins, including Aβ and tau inclusions which further inhibit the proteostasis (Keck et al., 2003; Almeida et al., 2006; Tseng et al., 2008), and the selective neuronal death (Dasuri et al., 2010; Hegde, 2010; Tramutola et al., 2016). It’s also worth stressing that the cerebral UPS activity -i.e., the overall capacity of the 26S proteasome to degrade polyubiquitinated proteins- has been found to be higher in neurons versus glia (Tydlacka et al., 2008) and at synapses than in the nucleus (Upadhya et al., 2006), indicating an its complex spatio-temporal regulation in brain in response to distinct cellular and subcellular compartments and/or different external stimuli (Haas and Broadie, 2008). Finally, to fully appreciate the causal role of changes in ubiquitin-dependent proteostasis at prodromal stage of AD neuropathology, it’s important to notice that the concentration of ubiquitin and UCHL-1 has been reported to increase in cerebrospinal fluid (CSF) from subjects with AD (Kandimalla et al., 2011; Oeckl et al., 2014; Heywood et al., 2015; Sjödin et al., 2017). Importantly, the quantitative determination of ubiquitin level in proteomic profiling of CSF from diseased cases shows strong predictive value by distinguishing between stable MCI and patients with MCI who progressed to AD (non-converters versus converters) (Simonsen et al., 2007).

## Conclusion

In conclusion, by biochemical, morphological, and electrophysiological experiments, we show a crucial role of UPS in the dysregulation of NGF/TrkA signaling on properties of cholinergic synapses by demonstrating that: (i) UPS activity participates in the NGF-dependent tuning of synaptic efficacy and presynaptic dynamics in cholinergic septo-hippocampal primary neurons via regulation of the steady-state levels of synapsin I, SNAP25 and α-synuclein; (ii) changes in ubiquitin homeostasis contributes to dysfunction of cholinergic synapses in aging Tg2576 transgenic AD animal model, even from presymptomatic stages of neuropathology (1-month-old). By giving novel insights into the molecular mechanisms underlying the loss/denervation of cholinergic nerve terminals occurring in incipient early/middle-stage of AD neuropathology, these findings from two well-established cellular and animal AD models provide potential therapeutic targets to contrast early cognitive and synaptic dysfunction associated to impairment in NGF/TrkA signaling pathway and selective degeneration of vulnerable BFCNs population.

## Author Contributions

GA made substantial contribution to the conception and design of the work, to the analysis and interpretation of data and wrote the manuscript. VL performed all the biochemical and morphological experiments, analyzed the data, and contributed to the writing of manuscript by revising it critically for important intellectual content. SC performed electrophysiological experiments and analyzed the data. CZ analyzed electrophysiological data and contributed to the critical reading of the manuscript. MC performed primary cultures of neurons. AB provided transgenic mice tissues and contributed to the critical reading of the manuscript. PC contributed to the conception of the work and to the writing of the manuscript. All authors contributed to manuscript revision, read and approved the submitted version.

## Conflict of Interest Statement

The authors declare that the research was conducted in the absence of any commercial or financial relationships that could be construed as a potential conflict of interest.

## Footnotes

1 http://imagej.nih.gov/ij/

## Supplementary Material

FIGURE S1 **(A)** Western blotting analysis was carried out on equal amounts of total protein extract (20 μg) from septal primary neurons cultured for 10–12 D.I.V. in defined medium supplemented with 0.2% B27, in the presence (*t*_0_) or absence (-1.5, -3, -6 h) of exogenously added NGF (100 ng/ml). Notice that cross-linking treatment of 0.1 μM filter with PFA 0.4% after transfer and before probing with anti-pan ubiquitin antibody (Dako antibody Z0458) increases the retention of low-molecular-weight monoubiquitin and improves the immunoreactivity signal. β-actin was used as loading control for each sample/lane. **(B)** Septal cholinergic-enriched cultures were grown continuously (10–12 D.I.V.) in 0.2% B27 media in the presence of exogenous NGF (100 ng/ml) from plating (*t*_0_) and then deprived of their trophic support for different periods of time (-1.5, -3, -6 h). Immunofluorescence staining with anti-pan ubiquitin antibody (Dako antibody Z0458) (green channel) followed confocal microscopy analysis was carried out to visualize the progressive formation of spheroid bodies (beading, varicosities) in nerve terminals (arrows) during the “dying-back” phenomenon triggered by NGF withdrawal. Images were representative of at least three independent experiments. Scale bar: 10 μM.

FIGURE S2 **(A,B)** Septal cholinergic-enriched cultures were grown continuously (10–12 D.I.V.) in 0.2% B27 media in the presence of exogenous NGF (100 ng/ml) from plating (*t*_0_) and then deprived of their trophic support for 6 h (-6 h) in the absence or presence of Z-VAD fmk (50 μM), a cell permeant irreversible pan-caspase inhibitor: Levels of SNAP25 and β-actin were assessed by Western blotting analysis **(A)** and densitometric quantification **(B)** was calculated as ratio of the value from control neurons (*t*_0_) and reported as mean ± SEM. Statistically significant differences were calculated at the respective experimental points by unpaired-two tailed *t*-Student’s test [^∗∗^*p* < 0.01 vs. *t*_0_ control neurons (°) and vs. -6 h NGF deprivation (§n.s., not significant)]. **(C)** Septal cholinergic-enriched cultures were grown continuously (10–12 D.I.V.) in 0.2% B27 media in the presence of exogenous NGF (100 ng/ml) from plating (*t*_0_) and then deprived of their trophic support for 6 h (-6 h) in the absence or presence of MG132 (10 μM) and BTZ (400 nM). Cell lysates were extracted and analyzed by Western blotting by probing for syntaxin I, NMDAR1 and synaptophysin. No change was detected on the protein expression level following -6 h NGF deprivation in the absence and in the presence of drugs. **(D,E)** Septal cholinergic-enriched cultures were grown continuously (10–12 D.I.V.) in 0.2% B27 media in the presence of exogenous NGF (100 ng/ml) from plating (*t*_0_) and treated with MG132 (10 μM) and BTZ (400 nM). DNA condensation-based assays with DAPI staining **(D)** detects no significant sign of apoptotic nuclei featured by of chromatin compaction. Scale bar: 10 μM. Cell viability was calculated by MTT assay after 6 h of incubation. The histogram **(E)** reports the neuronal survival calculated as the ratio of treated samples over untreated controls. Values are means of three experiments and no statistically significant differences were calculated by unpaired two-tailed *t*-Student’s test.

FIGURE S3 **(A,B)** Septal cholinergic-enriched cultures were grown continuously (10–12 D.I.V.) in 0.2% B27 media in the presence of exogenous NGF (100 ng/ml) from plating (*t*_0_) and then deprived of their trophic support for 6 h (-6 h) in the absence or presence of MG132 (10 μM) and BTZ (400 nM). Western blotting analysis on whole-cell lysates (40 μg) was performed to check the expression levels of free, monoubiquitin by probing with anti-pan ubiquitin antibody (Z0458 Dako). β-actin was used as internal control for samples loading **(A)** and densitometric quantification **(B)** was calculated as ratio of the value from control neurons (*t*_0_) and reported as mean ± SEM. Statistically significant differences were calculated by unpaired-two tailed *t*-Student’s test [^∗∗^*p* < 0.01 vs. *t*_0_ control neurons (°) and vs. -6 h NGF deprivation (§n.s., not significant)].

FIGURE S4 **(A,B)** Septal cholinergic-enriched cultures were grown continuously (10–12 D.I.V.) in 0.2% B27 media in the presence of exogenous NGF (100 ng/ml) from plating (*t*_0_) and then deprived of their trophic support for 6 h (-6 h) in the absence or presence of CQ (10 μM) and MG132 (10 μM). Western blotting analysis carried out on whole-cell extracts by probing for synapsin I, α-synuclein and β-actin **(A)** and relative densitometric quantification **(B)** were shown. Data were reported as mean ± SEM. and expressed ratio of the value from control neurons (*t*_0_). Statistically significant differences were calculated at the respective experimental points by unpaired-two tailed *t*-Student’s test [^∗∗^*p* < 0.01 vs. *t*_0_ control neurons (°) and vs. -6 h NGF deprivation (§)].

FIGURE S5 **(A,B)** Western blotting analysis for UCHL-1 **(A)** was carried out on equal amounts of total protein extract (20 μg) from septal primary neurons cultured for 10–12 D.I.V. in defined medium supplemented with 0.2% B27, in the presence (*t*_0_) or absence (-1.5, -3, -6 h) of exogenously added NGF (100 ng/ml). Relative densitometric quantification **(B)** by normalization on β-actin, which was used as internal control for samples loading, was reported. Data (*n* = 3) were reported as mean ± SEM. and expressed ratio of the value from control neurons (*t*_0_). Notice that there is in neuronal cultures an increase of the UCHL-1 expression level induced by the time-dependent NGF removal but values does not reach statistical significance by unpaired-two tailed *t*-Student’s test (-1.5 h *p* = 0.1750; -3 h *p* = 0.1424; -6 h, *p* < 0.0676 vs. *t*_0_ control neurons).

FIGURE S6 **(A,B)** Septal cholinergic-enriched cultures were grown continuously (10–12 D.I.V.) in 0.2% B27 media in the presence of exogenous NGF (100 ng/ml) from plating (*t*_0_) and then deprived of their trophic support for 6 h (-6 h) in the absence or presence of MG132 (2.5 μM), LDN (2.5 μM), and MG132 (2.5 μM)+LDN (2.5 μM) combination. Levels of synapsin I and synuclein were assessed by Western blotting analysis and β-actin was used as loading control **(A)**. Relative densitometric quantification **(B)** was calculated as ratio of the value from control neurons (*t*_0_) and reported as mean ± SEM. Statistically significant differences were calculated at the respective experimental points by unpaired-two tailed *t*-Student’s test [^∗^*p* < 0.05, ^∗∗^*p* < 0.01 vs. *t*_0_ control neurons (°) and vs. -6 h NGF deprivation (§)].

## Abbreviations

Aβ: amyloid-β peptides
AD: Alzheimer’s disease
APP: amyloid precursor protein
BFCNs: basal forebrain cholinergic neurons
ChAT: choline acetyltransferase
CNS: central nervous system
DUBs: deubiquitylating enzymes
HMW: high molecular-weight
LTP: long-term potentiation
M1: muscarinic receptor 1
MCI: mild cognitive impairment
mEPSCs: miniature post synaptic currents
NFT: neurofibrillary tangles
NGF: nerve growth factor
NR1: *N*-methyl-D-aspartate (NMDA) receptor subunit 1
Tg: transgenic
TrkA: tropomyosin receptor kinase A
UCHL-1: ubiquitin-C-terminal hydrolase 1
UPS: ubiquitin-proteasome system
vGAT: vesicular GABA transporter
vGLUT1: vesicular glutamate transporter 1
WT: wild-type

## References

[B1] Almeida C. G. Takahashi R. H. Gouras G. K. (2006). Beta-amyloid accumulation impairs multivesicular body sorting by inhibiting the ubiquitin-proteasome system. *J. Neurosci.* 26 4277–4288. 10.1523/JNEUROSCI.5078-05.2006 16624948 PMC6673997

[B2] Alvarez-Castelao B. Schuman E. M. (2015). The regulation of synaptic protein turnover. *J. Biol. Chem.* 290 28623–28630. 10.1074/jbc.R115.657130 26453306 PMC4661377

[B3] Amadoro G. Corsetti V. Stringaro A. Colone M. D’Aguanno S. Meli G. (2010). A NH2 tau fragment targets neuronal mitochondria at AD synapses: possible implications for neurodegeneration. *J. Alzheimers. Dis.* 21 445–470. 10.3233/JAD-2010-100120 20571215

[B4] Apelt J. Kumar A. Schliebs R. (2002). Impairment of cholinergic neurotransmission in adult and aged transgenic Tg2576 mouse brain expressing the Swedish mutation of human beta-amyloid precursor protein. *Brain Res.* 953 17–30. 10.1016/S0006-8993(02)03262-6 12384234

[B5] Arendt T. (2009). Synaptic degeneration in Alzheimer’s disease. *Acta Neuropathol.* 118 167–179. 10.1007/s00401-009-0536-x 19390859

[B6] Bell K. F. Ducatenzeiler A. Ribeiro-da-Silva A. Duff K. Bennett D. A. Cuello A. C. (2006). The amyloid pathology progresses in a neurotransmitter-specific manner. *Neurobiol. Aging* 27 1644–1657. 10.1016/j.neurobiolaging.2005.09.034 16271419

[B7] Bennett M. C. Bishop J. F. Leng Y. Chock P. B. Chase T. N. Mouradian M. M. (1999). Degradation of alpha-synuclein by proteasome. *J. Biol. Chem.* 274 33855–33858. 10.1074/jbc.274.48.33855 10567343

[B8] Bifsha P. Landry K. Ashmarina L. Durand S. Seyrantepe V. Trudel S. (2007). Altered gene expression in cells from patients with lysosomal storage disorders suggests impairment of the ubiquitin pathway. *Cell Death Differ.* 14 511–523. 10.1038/sj.cdd.4402013 16888648

[B9] Bingol B. Schuman E. M. (2005). Synaptic protein degradation by the ubiquitin proteasome system. *Curr. Opin. Neurobiol.* 15 536–541. 10.1016/j.conb.2005.08.016 16150592

[B10] Bingol B. Schuman E. M. (2006). Activity-dependent dynamics and sequestration of proteasomes in dendritic spines. *Nature* 441 1144–1148. 10.1038/nature04769 16810255

[B11] Bingol B. Sheng M. (2011). Deconstruction for reconstruction: the role of proteolysis in neural plasticity and disease. *Neuron* 69 22–32. 10.1016/j.neuron.2010.11.006 21220096

[B12] Bishop P. Rocca D. Henley J. M. (2016). Ubiquitin C-terminal hydrolase L1 (UCH-L1): structure, distribution and roles in brain function and dysfunction. *Biochem. J.* 473 2453–2462. 10.1042/BCJ20160082 27515257 PMC4980807

[B13] Bocchini V. Angeletti P. U. (1969). The nerve growth factor: purification as a 30,000-molecular-weight protein. *Proc. Natl. Acad. Sci. U.S.A.* 64 787–794. 10.1073/pnas.64.2.787 4982360 PMC223412

[B14] Brady S. Morfini G. (2010). A perspective on neuronal cell death signaling and neurodegeneration. *Mol. Neurobiol.* 42 25–31. 10.1007/s12035-010-8128-2 20480262 PMC3693570

[B15] Buac D. Shen M. Schmitt S. Kona F. R. Deshmukh R. Zhang Z. (2013). From bortezomib to other inhibitors of the proteasome and beyond. *Curr. Pharm. Des.* 19 4025–4038. 10.2174/1381612811319220012 23181572 PMC3657018

[B16] Caioli S. Curcio L. Pieri M. Antonini A. Marolda R. Severini C. (2011). Substance P receptor activation induces downregulation of the AMPA receptor functionality in cortical neurons from a genetic model of amyotrophic lateral sclerosis. *Neurobiol. Dis.* 44 92–101. 10.1016/j.nbd.2011.06.008 21726643

[B17] Campenot R. B. (1982). Development of sympathetic neurons in compartmentalized cultures. II. Local control of neurite growth by nerve growth factor. *Dev. Biol.* 93 13–21. 10.1016/0012-1606(82)90233-0 7128926

[B18] Canu N. Amadoro G. Triaca V. Latina V. Sposato V. Corsetti V. (2017). The intersection of NGF/TrkA signaling and amyloid precursor protein processing in alzheimer’s disease neuropathology. *Int. J. Mol. Sci.* 18:E1319. 10.3390/ijms18061319 28632177 PMC5486140

[B19] Cartier A. E. Djakovic S. N. Salehi A. Wilson S. M. Masliah E. Patrick G. N. (2009). Regulation of synaptic structure by ubiquitin C-terminal hydrolase L1. *J. Neurosci.* 29 7857–7868. 10.1523/JNEUROSCI.1817-09.2009 19535597 PMC2748938

[B20] Cattaneo A. Calissano P. (2012). Nerve growth factor and Alzheimer’s disease: new facts for an old hypothesis. *Mol. Neurobiol.* 46 588–604. 10.1007/s12035-012-8310-9 22940884

[B21] Chain D. G. Hegde A. N. Yamamoto N. Liu-Marsh B. Schwartz J. H. (1995). Persistent activation of cAMP-dependent protein kinase by regulated proteolysis suggests a neuron-specific function of the ubiquitin system in Aplysia. *J. Neurosci.* 15 7592–7603. 10.1523/JNEUROSCI.15-11-07592.1995 7472510 PMC6578082

[B22] Chauhan N. B. Siegel G. J. (2003). Effect of PPF and ALCAR on the induction of NGF- and p75-mRNA and on APP processing in Tg2576 brain. *Neurochem. Int.* 43 225–233. 10.1016/S0197-0186(03)00006-8 12689602

[B23] Chen P. C. Bhattacharyya B. J. Hanna J. Minkel H. Wilson J. A. Finley D. (2011). Ubiquitin homeostasis is critical for synaptic development and function. *J. Neurosci.* 31 17505–17513. 10.1523/JNEUROSCI.2922-11.2011 22131412 PMC3253363

[B24] Chen P. C. Qin L. N. Li X. M. Walters B. J. Wilson J. A. Mei L. (2009). The proteasome-associated deubiquitinating enzyme Usp14 is essential for the maintenance of synaptic ubiquitin levels and the development of neuromuscular junctions. *J. Neurosci.* 29 10909–10919. 10.1523/JNEUROSCI.2635-09.2009 19726649 PMC2766780

[B25] Chen X. Q. Sawa M. Mobley W. C. (2018). Dysregulation of neurotrophin signaling in the pathogenesis of Alzheimer disease and of Alzheimer disease in Down syndrome. *Free Radic. Biol. Med.* 114 52–61. 10.1016/j.freeradbiomed.2017.10.341 29031834 PMC5748266

[B26] Cheng J. North B. J. Zhang T. Dai X. Tao K. Guo J. (2018). The emerging roles of protein homeostasis-governing pathways in Alzheimer’s disease. *Aging Cell* 17:e12801. 10.1111/acel.12801 29992725 PMC6156496

[B27] Cho K. O. Hunt C. A. Kennedy M. B. (1992). The rat brain postresponses synaptic density fraction contains a homolog of the Drosophila discs-large tumor suppressor protein. *Neuron* 9 929–942. 10.1016/0896-6273(92)90245-9 1419001

[B28] Choi J. Levey A. I. Weintraub S. T. Rees H. D. Gearing M. Chin L. S. (2004). Oxidative modifications and down-regulation of ubiquitin carboxyl-terminal hydrolase L1 associated with idiopathic Parkinson’s and Alzheimer’s diseases. *J. Biol. Chem.* 279 13256–13264. 10.1074/jbc.M314124200 14722078

[B29] Ciechanover A. Kwon Y. T. (2015). Degradation of misfolded proteins in neurodegenerative diseases: therapeutic targets and strategies. *Exp. Mol. Med.* 47:e147. 10.1038/emm.2014.117 25766616 PMC4351408

[B30] Cline H. (2003). Synaptic plasticity: importance of proteasome-mediated protein turnover. *Curr. Biol.* 13 R514–R516. 10.1016/S0960-9822(03)00443-3 12842027

[B31] Cohen L. D. Ziv N. E. (2017). Recent insights on principles of synaptic protein degradation. *F1000Res.* 6:675. 10.12688/f1000research.10599.1 28620464 PMC5461898

[B32] Coleman P. D. Yao P. J. (2003). Synaptic slaughter in Alzheimer’s disease. *Neurobiol. Aging* 24 1023–1027. 10.1016/j.neurobiolaging.2003.09.001 14643374

[B33] Colledge M. Snyder E. M. Crozier R. A. Soderling J. A. Jin Y. Langeberg L. K. (2003). Ubiquitination regulates PSD-95 degradation and AMPA receptor surface expression. *Neuron* 40 595–607. 10.1016/S0896-6273(03)00687-1 14642282 PMC3963808

[B34] Conner J. M. Chiba A. A. Tuszynski M. H. (2005). The basal forebrain cholinergic system is essential for cortical plasticity and functional recovery following brain injury. *Neuron* 46 173–179. 10.1016/j.neuron.2005.03.003 15848797

[B35] Conner J. M. Culberson A. Packowski C. Chiba A. A. Tuszynski M. H. (2003). Lesions of the Basal forebrain cholinergic system impair task acquisition and abolish cortical plasticity associated with motor skill learning. *Neuron* 38 819–829. 10.1016/S0896-6273(03)00288-5 12797965

[B36] Corsetti V. Florenzano F. Atlante A. Bobba A. Ciotti M. T. Natale F. (2015). NH2-truncated human tau induces deregulated mitophagy in neurons by aberrant recruitment of Parkin and UCHL-1: implications in Alzheimer’s disease. *Hum. Mol. Genet.* 24 3058–3081. 10.1093/hmg/ddv059 25687137

[B37] Costes S. Huang C. J. Gurlo T. Daval M. Matveyenko A. V. Rizza R. A. (2011). β-cell dysfunctional ERAD/ubiquitin/proteasome system in type 2 diabetes mediated by islet amyloid polypeptide-induced UCH-L1 deficiency. *Diabetes Metab. Res. Rev.* 60 227–238. 10.2337/db10-0522 20980462 PMC3012175

[B38] Counts S. E. Mufson E. J. (2005). The role of nerve growth factor receptors in cholinergic basal forebrain degeneration in prodromal Alzheimer disease. *J. Neuropathol. Exp. Neurol.* 64 263–272. 10.1093/jnen/64.4.263 15835262

[B39] Coyle J. T. Price D. L. DeLong M. R. (1983). Alzheimer’s disease: a disorder of cortical cholinergic innervation. *Science* 219 1184–1190. 10.1126/science.6338589 6338589

[B40] Cuello A. C. Bruno M. A. Bell K. F. (2007). NGF-cholinergic dependency in brain aging, MCI and Alzheimer’s disease. *Curr. Alzheimer Res.* 4 351–358. 10.2174/156720507781788774 17908036

[B41] Dasuri K. Ebenezer P. J. Zhang L. Fernandez-Kim S. O. Uranga R. M. Gavilán E. (2010). Selective vulnerability of neurons to acute toxicity after proteasome inhibitor treatment: implications for oxidative stress and insolubility of newly synthesized proteins. *Free Radic. Biol. Med.* 49 1290–1297. 10.1016/j.freeradbiomed.2010.07.014 20678570 PMC3175605

[B42] Davies P. Maloney A. J. (1976). Selective loss of central cholinergic neurons in Alzheimer’s disease. *Lancet* 2:1403 10.1016/S0140-6736(76)91936-X 63862

[B43] Dawbarn D. Allen S. J. Semenenko F. M. (1988). Coexistence of choline acetyltransferase and nerve growth factor receptors in the rat basal forebrain. *Neurosci. Lett.* 94 138–144. 10.1016/0304-3940(88)90284-4 2853851

[B44] de Vrij F. M. Fischer D. F. van Leeuwen F. W. Hol E. M. (2004). Protein quality control in Alzheimer’s disease by the ubiquitin proteasome system. *Prog. Neurobiol.* 74 249–270. 10.1016/j.pneurobio.2004.10.001 15582222

[B45] Debeir T. Saragovi H. U. Cuello A. C. (1999). A nerve growth factor mimetic TrkA antagonist causes withdrawal of cortical cholinergic boutons in the adult rat. *Proc. Natl. Acad. Sci. U.S.A.* 96 4067–4072. 10.1073/pnas.96.7.4067 10097164 PMC22421

[B46] Ding M. Shen K. (2008). The role of the ubiquitin proteasome system in synapse remodeling and neurodegenerative diseases. *Bioessays* 30 1075–1083. 10.1002/bies.20843 18937340 PMC3095215

[B47] Ding Q. Dimayuga E. Martin S. Bruce-Keller A. J. Nukala V. Cuervo A. M. (2003). Characterization of chronic low-level proteasome inhibition on neural homeostasis. *J. Neurochem.* 86 489–497. 10.1046/j.1471-4159.2003.01885.x 12871590

[B48] Dong C. Upadhya S. C. Ding L. Smith T. K. Hegde A. N. (2008). Proteasome inhibition enhances the induction and impairs the maintenance of late-phase long-term potentiation. *Learn. Mem.* 15 335–347. 10.1101/lm.984508 18441292 PMC2364605

[B49] Ehlers M. D. (2003). Activity level controls postsynaptic composition and signaling via the ubiquitin-proteasomesystem. *Nat. Neurosci.* 6 231–242. 10.1038/nn1013 12577062

[B50] Emmerich C. H. Cohen P. (2015). Optimising methods for the preservation, capture and identification of ubiquitin chains and ubiquitylated proteins by immunoblotting. *Biochem. Biophys. Res. Commun.* 466 1–14. 10.1016/j.bbrc.2015.08.109 26325464 PMC4709362

[B51] Finley D. Bartel B. Varshavsky A. (1989). The tails of ubiquitin precursors are ribosomal proteins whose fusion to ubiquitin facilitates ribosome biogenesis. *Nature* 338 394–401. 10.1038/338394a0 2538753

[B52] Franco M. Seyfried N. T. Brand A. H. Peng J. Mayor U. (2011). A novel strategy to isolate ubiquitin conjugates reveals wide role for ubiquitination during neural development. *Mol. Cell. Proteomics* 10:M110.002188. 10.1074/mcp.M110.002188 20861518 PMC3098581

[B53] Gadhave K. Bolshette N. Ahire A. Pardeshi R. Thakur K. Trandafir C. (2016). The ubiquitin proteasomal system: a potential target for the management of Alzheimer’s disease. *J. Cell. Mol. Med.* 20 1392–1407. 10.1111/jcmm.12817 27028664 PMC4929298

[B54] Gau J. T. Steinhilb M. L. Kao T. C. D’Amato C. J. Gaut J. R. Frey K. A. (2002). Stable beta-secretase activity and presynaptic cholinergic markers during progressive central nervous system amyloidogenesis in Tg2576 mice. *Am. J. Pathol.* 160 731–738. 10.1016/S0002-9440(10)64893-6 11839594 PMC1850661

[B55] Geula C. Nagykery N. Nicholas A. Wu C. K. (2008). Cholinergic neuronal and axonal abnormalities are present early in aging and in Alzheimer disease. *J. Neuropathol. Exp. Neurol.* 67 309–318. 10.1097/NEN.0b013e31816a1df3 18379437 PMC3243760

[B56] Gillardon F. Kloss A. Berg M. Neumann M. Mechtler K. Hengerer B. (2007). The 20S proteasome isolated from Alzheimer’s disease brain shows post-translational modifications but unchanged proteolytic activity. *J. Neurochem.* 101 1483–1490. 10.1111/j.1471-4159.2006.04438.x 17286585

[B57] Gong B. Cao Z. Zheng P. Vitolo O. V. Liu S. Staniszewski A. (2006). Ubiquitin hydrolase Uch-L1 rescues beta-amyloid-induced decreases in synaptic function and contextual memory. *Cell* 126 775–788. 10.1016/j.cell.2006.06.046 16923396

[B58] Grice G. L. Nathan J. A. (2016). The recognition of ubiquitinated proteins by the proteasome. *Cell. Mol. Life Sci.* 73 3497–3506. 10.1007/s00018-016-2255-5 27137187 PMC4980412

[B59] Griffin J. W. Watson D. F. (1988). Axonal transport in neurological disease. *Ann. Neurol.* 23 3–13. 10.1002/ana.410230103 3278671

[B60] Gupta R. Lan M. Mojsilovic-Petrovic J. Choi W. H. Safren N. Barmada S. (2017). The proline/arginine dipeptide from hexanucleotide repeat expanded C90RF72 inhibits the proteasome. *eNeuro* 4:ENEURO.249–ENEURO.216. 10.1523/ENEURO.0249-16.2017 28197542 PMC5282547

[B61] Gylys K. H. Fein J. A. Yang F. Cole G. M. (2004). Enrichment of presynaptic and postsynaptic markers by size-based gating analysis of synaptosome preparations from rat and human cortex. *Cytometry* 60 90–96. 10.1002/cyto.a.20031 15229861

[B62] Haas K. F. Broadie K. (2008). Roles of ubiquitination at the synapse. *Biochim. Biophys. Acta* 1779 495–506. 10.1016/j.bbagrm.2007.12.010 18222124 PMC2668815

[B63] Hakim V. Cohen L. D. Zuchman R. Ziv T. Ziv N. E. (2016). The effects of proteasomal inhibition on synaptic proteostasis. *EMBO J.* 35 2238–2262. 10.15252/embj.201593594 27613546 PMC5069550

[B64] Hallengren J. Chen P. C. Wilson S. M. (2013). Neuronal ubiquitin homeostasis. *Cell Biochem. Biophys.* 67 67–73. 10.1007/s12013-013-9634-4 23686613 PMC3758786

[B65] Hampel H. Mesulam M. M. Cuello A. C. Farlow M. R. Giacobini E. Grossberg G. T. (2018). Cholinergic system working group. the cholinergic system in the pathophysiology and treatment of Alzheimer’s disease. *Brain* 141 1917–1933. 10.1093/brain/awy132 29850777 PMC6022632

[B66] Hanus C. Schuman E. M. (2013). Proteostasis in complex dendrites. *Nat. Rev. Neurosci.* 14 638–648. 10.1038/nrn3546 23900412

[B67] Hartikka J. Hefti F. (1988a). Comparison of nerve growth factor’s effects on development of septum, striatum, and nucleus basalis cholinergic neurons *in vitro*. *J. Neurosci. Res.* 21 352–364. 10.1002/jnr.490210227 3216428

[B68] Hartikka J. Hefti F. (1988b). Development of septal cholinergic neurons in culture: plating density and glial cells modulate effects of NGF on survival, fiber growth, and expression of transmitter-specific enzymes. *J. Neurosci.* 8 2967–2985. 10.1523/JNEUROSCI.08-08-02967.1988 2842468 PMC6569420

[B69] Hasselmo M. E. Giocomo L. G. (2006). Cholinergic modulation of cortical function. *J. Mol. Neurosci.* 30 133–135. 10.1385/JMN:30:1:133 17192659

[B70] Hefti F. Weiner W. J. (1986). Nerve growth factor and Alzheimer’s disease. *Ann. Neurol.* 20 275–281. 10.1002/ana.410200302 3532929

[B71] Hegde A. N. (2004). Ubiquitin-proteasome-mediated local protein degradation and synapti plasticity. *Prog. Neurobiol.* 73 311–357. 10.1016/j.pneurobio.2004.05.005 15312912

[B72] Hegde A. N. (2010). The ubiquitin-proteasome pathway and synaptic plasticity. *Learn. Mem.* 17 314–327. 10.1101/lm.1504010 20566674 PMC2904103

[B73] Hegde A. N. Upadhya S. C. (2007). The ubiquitin-proteasome pathway in health and disease of the nervous system. *Trends Neurosci.* 30 587–595. 10.1016/j.tins.2007.08.005 17950927

[B74] Heinemeyer W. Simeon A. Hirsch H. H. Schiffer H. H. Teichert U. Wolf D. H. (1991). Lysosomal and non-lysosomal proteolysis in the eukaryotic cell: studies on yeast. *Biochem. Soc. Trans.* 19 724–725. 10.1042/bst0190724 1783206

[B75] Heywood W. E. Galimberti D. Bliss E. Sirka E. Paterson R. W. Magdalinou N. K. (2015). Identification of novel CSF biomarkers for neurodegeneration and their validation by a high-throughput multiplexed targeted proteomic assay. *Mol. Neurodegener.* 11:20. 10.1186/s13024-015-0059-y 26907468 PMC4763435

[B76] Hicke L. (2001). Protein regulation by monoubiquitin. *Nat. Rev. Mol. Cell. Biol.* 2 195–201. 10.1038/35056583 11265249

[B77] Hicke L. Dunn R. (2003). Regulation of membrane protein transport by ubiquitin and ubiquitin-binding proteins. *Annu. Rev. Cell. Dev. Biol.* 19 141–172. 10.1146/annurev.cellbio.19.110701.154617 14570567

[B78] Holtzman D. M. Li Y. Parada L. F. Kinsman S. Chen C. K. Valletta J. S. (1992). p140trk mRNA marks NGF-responsive forebrain neurons: evidence that trk gene expression is induced by NGF. *Neuron* 9 465–478. 10.1016/0896-6273(92)90184-F 1524827

[B79] Honer W. G. (2003). Pathology of presynaptic proteins in Alzheimer’s disease: more than simple loss of terminals. *Neurobiol. Aging* 24 1047–1062. 10.1016/j.neurobiolaging.2003.04.005 14643376

[B80] Hoopfer E. D. McLaughlin T. Watts R. J. Schuldiner O. O’Leary D. D. Luo L. (2006). Wlds protection distinguishes axon degeneration following injury from naturally occurring developmental pruning. *Neuron* 50 883–895. 10.1016/j.neuron.2006.05.013 16772170

[B81] Hsiao K. Chapman P. Nilsen S. Eckman C. Harigaya Y. Younkin S. (1996). Correlative memory deficits, Abeta elevation, and amyloid plaques in transgenic mice. *Science* 274 99–102. 10.1126/science.274.5284.99 8810256

[B82] Huang Q. Wang H. Perry S. W. Figueiredo-Pereira M. E. (2013). Negative regulation of 26S proteasome stability via calpain-mediated cleavage of Rpn10 subunit upon mitochondrial dysfunction in neurons. *J. Biol. Chem.* 288 12161–12174. 10.1074/jbc.M113.464552 23508964 PMC3636900

[B83] Huh C. Y. Danik M. Manseau F. Trudeau L. E. Williams S. (2008). Chronic exposure to nerve growth factor increases acetylcholine and glutamate release from cholinergic neurons of the rat medial septum and diagonal band of Broca via mechanisms mediated by p75NTR. *J. Neurosci.* 28 1404–1409. 10.1523/JNEUROSCI.4851-07.2008 18256260 PMC6671585

[B84] Ichihara N. Wu J. Chui D. H. Yamazaki K. Wakabayashi T. Kikuchi T. (1995). Axonal degeneration promotes abnormal accumulation of amyloid beta-protein in ascending gracile tract of gracile axonal dystrophy (GAD) mouse. *Brain Res.* 695 173–178. 10.1016/0006-8993(95)00729-A 8556328

[B85] Irizarry M. C. McNamara M. Fedorchak K. Hsiao K. Hyman B. T. (1997). APPSw transgenic mice develop age-related A beta deposits and neuropil abnormalities, but no neuronal loss in CA1. *J. Neuropathol. Exp. Neurol.* 56 965–973. 10.1097/00005072-199709000-00002 9291938

[B86] Isacson O. Seo H. Lin L. Albeck D. Granholm A. C. (2002). Alzheimer’s disease and Down’s syndrome: roles of APP, trophic factors and ACh. *Trends Neurosci.* 25 79–84. 10.1016/S0166-2236(02)02037-4 11814559

[B87] Jiang X. Litkowski P. E. Taylor A. A. Lin Y. Snider B. J. Moulder K. L. (2010). A role for the ubiquitin-proteasome system in activity-dependent presynaptic silencing. *J. Neurosci.* 30 798–1809. 10.1523/JNEUROSCI.4965-09.2010 20130189 PMC2824895

[B88] Kabeya Y. Mizushima N. Ueno T. Yamamoto A. Kirisako T. Noda T. (2000). LC3, a mammalian homologue of yeast Apg8p, is localized in autophagosome membranes after processing. *EMBO J.* 19 5720–5728. 10.1093/emboj/19.21.5720 11060023 PMC305793

[B89] Kalla S. Stern M. Basu J. Varoqueaux F. Reim K. Rosenmund C. (2006). Molecular dynamics of a presynaptic active zone protein studied in Munc13-1-enhanced yellow fluorescent protein knock-in mutant mice. *J. Neurosci.* 26 13054–13066. 10.1523/JNEUROSCI.4330-06.2006 17167095 PMC6674949

[B90] Kandimalla R. J. S P. Bk B. Wani W. Y. Sharma D. R. Grover V. K. (2011). Cerebrospinal fluid profile of amyloid β42 (Aβ42), hTau and ubiquitin in North Indian Alzheimer’s disease patients. *Neurosci. Lett.* 487 134–138. 10.1016/j.neulet.2010.06.075 20599474

[B91] Kawarabayashi T. Younkin L. H. Saido T. C. Shoji M. Ashe K. H. Younkin S. G. (2001). Age-dependent changes in brain, CSF, and plasma amyloid (beta) protein in the Tg2576 transgenic mouse model of Alzheimer’s disease. *J. Neurosci.* 21 372–381. 10.1523/JNEUROSCI.21-02-00372.2001 11160418 PMC6763819

[B92] Keck S. Nitsch R. Grune T. Ullrich O. (2003). Proteasome inhibition by paired helical filament-tau in brains of patients with Alzheimer’s disease. *J. Neurochem.* 85 115–122. 10.1046/j.1471-4159.2003.01642.x 12641733

[B93] Keller J. N. Hanni K. B. Markesbery W. R. (2000). Impaired proteasome function in Alzheimer’s disease. *J. Neurochem.* 75 436–439. 10.1046/j.1471-4159.2000.0750436.x 10854289

[B94] King D. L. Arendash G. W. (2002). Behavioral characterization of the Tg2576 transgenic model of Alzheimer’s disease through 19 months. *Physiol. Behav.* 75 627–642. 10.1016/S0031-9384(02)00639-X 12020728

[B95] Klingner M. Apelt J. Kumar A. Sorger D. Sabri O. Steinbach J. (2003). Alterations in cholinergic and non-cholinergic neurotransmitter receptor densities in transgenic Tg2576 mouse brain with beta-amyloid plaque pathology. *Int. J. Dev. Neurosci.* 21 357–369. 10.1016/j.ijdevneu.2003.08.001 14599482

[B96] Klionsky D. J. Abdalla F. C. Abeliovich H. Abraham R. T. Acevedo-Arozena A. Adeli K. (2012). Guidelines for the use and interpretation of assays for monitoring autophagy. *Autophagy* 8 445–544. 10.4161/auto.19496 22966490 PMC3404883

[B97] Klionsky D. J. Elazar Z. Seglen P. O. Rubinsztein D. C. (2008). Does bafilomycin A1 block the fusion of autophagosomes with lysosomes? *Autophagy* 4 849–850. 18758232 10.4161/auto.6845

[B98] Kobayashi D. T. Chen K. S. (2005). Behavioral phenotypes of amyloid-based genetically modified mouse models of Alzheimer’s disease. *Genes Brain Behav.* 4 173–196. 10.1111/j.1601-183X.2005.00124.x 15810905

[B99] Korhonen L. Lindholm D. (2004). The ubiquitin proteasome system in synaptic and axonal degeneration: a new twist to an old cycle. *J. Cell Biol.* 165 27–30. 10.1083/jcb.200311091 15067020 PMC2172081

[B100] Korolchuk V. I. Menzies F. M. Rubinsztein D. C. (2010). Mechanisms of cross-talk between the ubiquitin-proteasome and autophagy-lysosome systems. *FEBS Lett.* 584 1393–1398. 10.1016/j.febslet.2009.12.047 20040365

[B101] Kroemer G. Mariño G. Levine B. (2010). Autophagy and the integrated stress response. *Mol. Cell* 40 280–293. 10.1016/j.molcel.2010.09.023 20965422 PMC3127250

[B102] Kyratzi E. Pavlaki M. Stefanis L. (2008). The S18Y polymorphic variant of UCH-L1 confers an antioxidant function to neuronal cells. *Hum. Mol. Genet.* 17 2160–2171. 10.1093/hmg/ddn115 18411255

[B103] Larsen C. N. Krantz B. A. Wilkinson K. D. (1998). Substrate specificity of deubiquitinating enzymes: ubiquitin C-terminal hydrolases. *Biochemistry* 37 3358–3368. 10.1021/bi972274d 9521656

[B104] Latina V. Caioli S. Zona C. Ciotti M. T. Amadoro G. Calissano P. (2017). Impaired NGF/TrkA signaling causes early AD-linked presynaptic dysfunction in cholinergic primary neurons. *Front. Cell Neurosci.* 11:68. 10.3389/fncell.2017.00068 28360840 PMC5350152

[B105] Laursen B. Mørk A. Plath N. Kristiansen U. Bastlund J. F. (2014). Impaired hippocampal acetylcholine release parallels spatial memory deficits in Tg2576 mice subjected to basal forebrain cholinergic degeneration. *Brain Res.* 1543 253–262. 10.1016/j.brainres.2013.10.055 24231553

[B106] Lazarevic V. Schone C. Heine M. Gundelfinger E. D. Fejtova A. (2011). Extensive remodeling of the presynaptic cytomatrix upon homeostatic adaptation to network activity silencing. *J. Neurosci.* 31 10189–10200. 10.1523/JNEUROSCI.2088-11.2011 21752995 PMC6623065

[B107] Leitch V. Agre P. King L. S. (2001). Altered ubiquitination and stability of aquaporin-1 in hypertonic stress. *Proc. Natl. Acad. Sci. U.S.A.* 98 2894–2898. 10.1073/pnas.041616498 11226337 PMC30236

[B108] Liu C. W. Corboy M. J. DeMartino G. N. Thomas P. J. (2003). Endoproteolytic activity of the proteasome. *Science* 299 408–411. 10.1126/science.1079293 12481023 PMC3516294

[B109] Liu Y. Fallon L. Lashuel H. A. Liu Z. Lansbury TTJr (2002). The UCH-L1 gene encodes two opposing enzymatic activities that affect alpha-synuclein degradation and Parkinson’s disease susceptibility. *Cell* 111 209–218. 10.1016/S0092-8674(02)01012-7 12408865

[B110] López T. Silva-Ayala D. López S. Arias C. F. (2011). Replication of the rotavirus genome requires an active ubiquitin-proteasome system. *J. Virol.* 85 11964–11971. 10.1128/JVI.05286-11 21900156 PMC3209302

[B111] López Salon M. Morelli L. Castaño E. M. Soto E. F. Pasquini J. M. (2000). Defective ubiquitination of cerebral proteins in Alzheimer’s disease. *J. Neurosci. Res.* 62 302–310. 10.1002/1097-4547(20001015)62:2<302::AID-JNR15>3.0.CO;2-L 11020223

[B112] Lüth H. J. Apelt J. Ihunwo A. O. Arendt T. Schliebs R. (2003). Degeneration of beta-amyloid-associated cholinergic structures in transgenic APP SW mice. *Brain Res.* 977 16–22. 10.1016/S0006-8993(03)02658-1 12788508

[B113] MacInnis B. L. Campenot R. B. (2005). Regulation of Wallerian degeneration and nerve growth factor withdrawal-induced pruning of axons of sympathetic neurons by the proteasome and the MEK/Erk pathway. *Mol. Cell. Neurosci.* 28 430–439. 10.1016/j.mcn.2004.10.003 15737734

[B114] Mandelkow E. M. Stamer K. Vogel R. Thies E. Mandelkow E. (2003). Clogging of axons by tau, inhibition of axonal traffic and starvation of synapses. *Neurobiol. Aging* 24 1079–1085. 10.1016/j.neurobiolaging.2003.04.007 14643379

[B115] McKinney M. Coyle J. T. Hedreen J. C. (1983). Topographic analysis of the innervation of the rat neocortex and hippocampus by the basal forebrain cholinergic system. *J. Comp. Neurol.* 217 103–121. 10.1002/cne.902170109 6875049

[B116] McMillan L. E. Brown J. T. Henley J. M. Helena Cimarostia H. (2011). Profiles of SUMO and ubiquitin conjugation in an Alzheimer’s disease model. *Neurosci. Lett.* 502 201–208. 10.1016/j.neulet.2011.07.045 21843595 PMC3176896

[B117] Mufson E. J. Counts S. E. Ginsberg S. D. (2002). Gene expression profiles of cholinergic nucleus basalis neurons in Alzheimer’s disease. *Neurochem. Res.* 27 1035–1048. 10.1023/A:1020952704398 12462403

[B118] Mufson E. J. Counts S. E. Perez S. E. Ginsberg S. D. (2008). Cholinergic system during the progression of Alzheimer’s disease: therapeutic implications. *Expert Rev. Neurother.* 8 1703–1718. 10.1586/14737175.8.11.1703 18986241 PMC2631573

[B119] Mufson E. J. He B. Nadeem M. Perez S. E. Counts S. E. Leurgans S. (2012). Hippocampal proNGF signaling pathways and beta-amyloid levels in mild cognitive impairment and Alzheimer disease. *J. Neuropathol. Exp. Neurol.* 71 1018–1029. 10.1097/NEN.0b013e318272caab 23095849 PMC3481187

[B120] Mufson E. J. Ma S. Y. Cochran E. J. Bennett D. A. Beckett L. A. Jaffar S. (2000). Loss of nucleus basalis neurons containing trkA immunoreactivity in individuals with mild cognitive impairment and early Alzheimer’s disease. *J. Comp. Neurol.* 427 19–30. 10.1002/1096-9861(20001106)427:1<19::AID-CNE2>3.0.CO;2-A 11042589

[B121] Myeku N. Figueiredo-Pereira M. E. (2011). Dynamics of the degradation of ubiquitinated proteins by proteasomes and autophagy: association with sequestosome 1/p62. *J. Biol. Chem.* 286 22426–22440. 10.1074/jbc.M110.149252 21536669 PMC3121389

[B122] Myeku N. Metcalfe M. J. Huang Q. Figueiredo-Pereira M. (2011). Assessment of proteasome impairment and accumulation/aggregation of ubiquitinated proteins in neuronal cultures. *Methods Mol. Biol.* 793 273–296. 10.1007/978-1-61779-328-8_18 21913107 PMC3408317

[B123] Myung J. Kim K. B. Crews C. M. (2001). The ubiquitin-proteasome pathway and proteasome inhibitors. *Med. Res. Rev.* 21 245–273. 10.1002/med.1009 11410931 PMC2556558

[B124] Na C. H. Jones D. R. Yang Y. Wang X. Xu Y. Peng J. (2012). Synaptic protein ubiquitination in rat brain revealed by antibody-based ubiquitome analysis. *J. Proteome Res.* 11 4722–4732. 10.1021/pr300536K 22871113 PMC3443409

[B125] Navon A. Ciechanover A. (2009). The 26 S proteasome: from basic mechanisms to drug targeting. *J. Biol. Chem.* 284 33713–33718. 10.1074/jbc.R109.018481 19812037 PMC2797140

[B126] Niewiadomska G. Mietelska-Porowska A. Mazurkiewicz M. (2011). The cholinergic system, nerve growth factor and the cytoskeleton. *Behav. Brain Res.* 221 515–526. 10.1016/j.bbr.2010.02.024 20170684

[B127] Noor N. M. Møllgård K. Wheaton B. J. Steer D. L. Truettner J. S. Dziegielewska K. M. (2013). Expression and cellular distribution of ubiquitin in response to injury in the developing spinal cord of Monodelphis domestica. *PLoS One* 8:e62120. 10.1371/journal.pone.0062120 23626776 PMC3633899

[B128] Obin M. Mesco E. Gong X. Haas A. L. Joseph J. Taylor A. (1999). Neurite outgrowth in PC12 cells. Distinguishing the roles of ubiquitylation and ubiquitin-dependent proteolysis. *J. Biol. Chem.* 274 11789–11795. 10.1074/jbc.274.17.11789 10206996

[B129] Oddo S. Billings L. Kesslak J. P. Cribbs D. H. LaFerla F. M. (2004). Abeta immunotherapy leads to clearance of early, but not late, hyperphosphorylated tau aggregates via the proteasome. *Neuron* 43 321–332. 10.1016/j.neuron.2004.07.003 15294141

[B130] Oeckl P. Steinacker P. von Arnim C. A. Straub S. Nagl M. Feneberg E. (2014). Intact protein analysis of ubiquitin in cerebrospinal fluid by multiple reaction monitoring reveals differences in Alzheimer’s disease and frontotemporal lobar degeneration. *J. Proteome Res.* 13 4518–4525. 10.1021/pr5006058 25091646

[B131] Oh S. Hong H. S. Hwang E. Sim H. J. Lee W. Shin S. J. (2005). Amyloid peptide attenuates the proteasome activity in neuronal cells. *Mech. Ageing Dev.* 126 1292–1299. 10.1016/j.mad.2005.07.006 16153690

[B132] Ohtani-Kaneko R. Asahara M. Takada K. Kanda T. Iigo M. Hara M. (1996). Nerve growth factor (NGF) induces increase in multi-ubiquitin chains and concomitant decrease in free ubiquitin in nuclei of PC12h. *Neurosci. Res.* 26 349–355. 10.1016/S0168-0102(96)01117-0 9004273

[B133] Okada K. Wangpoengtrakul C. Osawa T. Toyokuni S. Tanaka K. Uchida K. (1999). 4-Hydroxy-2-nonenal-mediated impairment of intracellular proteolysis during oxidative stress. Identification of proteasomes as target molecules. *J. Biol. Chem.* 274 23787–23793. 10.1074/jbc.274.34.23787 10446139

[B134] Orlowski M. Wilk S. (2000). Catalytic activities of the 20 S proteasome, a multicatalytic proteinase complex. *Arch. Biochem. Biophys.* 383 1–16. 10.1006/abbi.2000.2036 11097171

[B135] Osaka H. Wang Y. L. Takada K. Takizawa S. Setsuie R. Li H. (2003). Ubiquitin carboxy-terminal hydrolase L1 binds to and stabilizes monoubiquitin in neuron. *Hum. Mol. Genet.* 12 1945–1958. 10.1093/hmg/ddg211 12913066

[B136] Overk C. R. Masliah E. (2014). Pathogenesis of synaptic degeneration in Alzheimer’s disease and Lewy body disease. *Biochem. Pharmacol.* 88 508–516. 10.1016/j.bcp.2014.01.015 24462903 PMC3973539

[B137] Pasinetti G. M. (2001). Use of cDNA microarray in the search for molecular markers involved in the onset of Alzheimer’s disease dementia. *J. Neurosci. Res.* 65 471–476. 10.1002/jnr.1176 11550214

[B138] Patnaik A. Chau V. Wills J. W. (2000). Ubiquitin is part of the retrovirus budding machinery. *Proc. Natl. Acad. Sci. U.S.A.* 97 13069–13074. 10.1073/pnas.97.24.13069 11087861 PMC27179

[B139] Patrick G. N. (2006). Synapse formation and plasticity: recent insights from the perspective of the ubiquitin proteasome system. *Curr. Opin. Neurobiol.* 16 90–94. 10.1016/j.conb.2006.01.007 16427269

[B140] Perry E. K. Tomlinson B. E. Blessed G. Bergmann K. Gibson P. H. Perry R. H. (1978). Correlation of cholinergic abnormalities with senile plaques and mental test scores in senile dementia. *Br. Med. J.* 2 1457–1459. 10.1136/bmj.2.6150.1457 719462 PMC1608703

[B141] Pompl P. N. Mullan M. J. Bjugstad K. Arendash G. W. (1999). Adaptation of the circular platform spatial memory task for mice: use in detecting cognitive impairment in the APP(SW) transgenic mouse model for Alzheimer’s disease. *J. Neurosci. Methods* 87 87–95. 10.1016/S0165-0270(98)00169-1 10065997

[B142] Ramirez C. N. Antczak C. Djaballah H. (2010). Cell viability assessment: toward content-rich platforms. *Expert Opin. Drug Discov.* 5 223–233. 10.1517/17460441003596685 22823019 PMC3640448

[B143] Reddy P. H. Mani G. Park B. S. Jacques J. Murdoch G. Whetsell WJr (2005). Differential loss of synaptic proteins in Alzheimer’s disease: implications for synaptic dysfunction. *J. Alzheimers Dis.* 7 103–117. 10.3233/JAD-2005-7203 15851848

[B144] Rinetti G. V. Schweizer F. E. (2010). Ubiquitination acutely regulates presynaptic neurotransmitter release in mammalian neurons. *J. Neurosci.* 30 3157–3166. 10.1523/JNEUROSCI.3712-09.2010 20203175 PMC2905680

[B145] Rizzoli S. O. (2014). Synaptic vesicle recycling: steps and principles. *EMBO J.* 33 788–822. 10.1002/embj.201386357 24596248 PMC4194108

[B146] Roy S. Zhang B. Lee V. M. Trojanowski J. Q. (2005). Axonal transport defects: a common theme in neurodegenerative diseases. *Acta Neuropathol.* 109 5–13. 10.1007/s00401-004-0952-x 15645263

[B147] Saba L. Viscomi M. T. Caioli S. Pignataro A. Bisicchia E. Pieri M. (2016). Altered functionality, morphology, and vesicular glutamate transporter expression of cortical motor neurons from a presymptomatic mouse model of amyotrophic lateral sclerosis. *Cereb. Cortex* 26 1512–1528. 10.1093/cercor/bhu317 25596588

[B148] Sadoul R. Fernandez P. A. Quiquerez A. L. Martinou I. Maki M. Schröter M. (1996). Involvement of the proteasome in the programmed cell death of NGF-deprived sympathetic neurons. *EMBO J.* 15 3845–3852. 10.1002/j.1460-2075.1996.tb00758.x 8670889 PMC452074

[B149] Saigoh K. Wang Y. L. Suh J. G. Yamanishi T. Sakai Y. Kiyosawa H. (1999). Intragenic deletion in the gene encoding ubiquitin carboxy-terminal hydrolase in gad mice. *Nat. Genet.* 23 47–51. 10.1038/12647 10471497

[B150] Sakurai M. Sekiguchi M. Zushida K. Yamada K. Nagamine S. Kabuta T. (2008). Reduction in memory in passive avoidance learning, exploratory behaviour and synaptic plasticity in mice with a spontaneous deletion in the ubiquitin C-terminal hydrolase L1 gene. *Eur. J. Neurosci.* 27 691–701. 10.1111/j.1460-9568.2008.06047.x 18279321

[B151] Salehi A. Delcroix J. D. Mobley W. C. (2003). Traffic at the intersection of neurotrophic factor signaling and neurodegeneration. *Trends Neurosci.* 26 73–80. 10.1016/S0166-2236(02)00038-3 12536130

[B152] Santos A. R. Mele M. Vaz S. H. Kellermayer B. Grimaldi M. Colino-Oliveira M. (2015). Differential role of the proteasome in the early and late phases of BDNF-induced facilitation of LTP. *J. Neurosci.* 35 3319–3329. 10.1523/JNEUROSCI.4521-14.2015 25716833 PMC6605551

[B153] Schliebs R. Arendt T. (2006). The significance of the cholinergic system in the brain during aging and in Alzheimer’s disease. *J. Neural Transm.* 113 1625–1644. 10.1007/s00702-006-0579-2 17039298

[B154] Schliebs R. Arendt T. (2011). The cholinergic system in aging and neuronal degeneration. *Behav. Brain Res.* 221 555–563. 10.1016/j.bbr.2010.11.058 21145918

[B155] Segref A. Hoppe T. (2009). Think locally: control of ubiquitin-dependent protein degradation in neurons. *EMBO Rep.* 10 44–50. 10.1038/embor.2008.229 19079132 PMC2613211

[B156] Selkoe D. J. (2002). Alzheimer’s disease is a synaptic failure. *Science* 298 789–791. 10.1126/science.1074069 12399581

[B157] Seo H. Isacson O. (2010). The hAPP-YAC transgenic model has elevated UPS activity in the frontal cortex similar to Alzheimer’s disease and Down’s syndrome. *J. Neurochem.* 114 1819–1826. 10.1111/j.1471-4159.2010.06902.x 20698932

[B158] Sharma N. Deppmann C. D. Harrington A. W. Hillaire C. S. T. Chen Z.-Y. Lee F. L. (2010). Long-distance control of synapse assembly by target-derived NGF. *Neuron* 67 422–434. 10.1016/j.neuron.2010.07.018 20696380 PMC2949359

[B159] Shin S. M. Zhang N. Hansen J. Gerges N. Z. Pak D. T. Sheng M. (2012). GKAP orchestrates activity-dependent postsynaptic protein remodeling and homeostatic scaling. *Nat. Neurosci.* 15 1655–1666. 10.1038/nn.3259 23143515 PMC3804128

[B160] Shringarpure R. Grune T. Sitte N. Davies K. J. (2000). 4-Hydroxynonenal-modified amyloid-beta peptide inhibits the proteasome: possible importance in Alzheimer’s disease. *Cell Mol. Life Sci.* 57 1802–1809. 10.1007/PL00000660 11130184 PMC11149552

[B161] Simmons D. A. Knowles J. K. Belichenko N. P. Banerjee G. Finkle C. Massa S. M. (2014). A small molecule p75NTR ligand, LM11A-31, reverses cholinergic neurite dystrophy in Alzheimer’s disease mouse models with mid- to late-stage disease progression. *PLoS One* 9:e102136. 10.1371/journal.pone.0102136 25153701 PMC4143160

[B162] Simonsen A. H. McGuire J. Hansson O. Zetterberg H. Podust V. N. Davies H. A. (2007). Novel panel of cerebrospinal fluid biomarkers for the prediction of progression to Alzheimer dementia in patients with mild cognitive impairment. *Arch. Neurol.* 64 366–370. 10.1001/archneur.64.3.366 17353378

[B163] Sjödin S. Hansson O. Öhrfelt A. Brinkmalm G. Zetterberg H. Brinkmalm A. (2017). Mass spectrometric analysis of cerebrospinal fluid ubiquitin in alzheimer’s disease and parkinsonian disorders. *Proteomics Clin. Appl.* 11:1700100. 10.1002/prca.201700100 28972305 PMC5765402

[B164] Snider B. J. Tee L. Y. Canzoniero L. M. Babcock D. J. Choi D. W. (2002). NMDA antagonists exacerbate neuronal death caused by proteasome inhibition in cultured cortical and striatal neurons. *Eur. J. Neurosci.* 15 419–428. 10.1046/j.0953-816x.2001.01867.x 11876769

[B165] Sobreviela T. Clary D. O. Reichardt L. F. Brandabur M. M. Kordower J. H. Mufson E. J. (1994). TrkA-immunoreactive profiles in the central nervous system: colocalization with neurons containing p75 nerve growth factor receptor, choline acetyltransferase, and serotonin. *J. Comp. Neurol.* 350 587–611. 10.1002/cne.903500407 7890832 PMC2710128

[B166] Sofroniew M. V. Galletly N. P. Isacson O. Svendsen C. N. (1990). Survival of adult basal forebrain cholinergic neurons after loss of target neurons. *Science* 247 338–342. 10.1126/science.1688664 1688664

[B167] Speese S. D. Trotta N. Rodesch C. K. Aravamudan B. Broadie K. (2003). The ubiquitin proteasome system acutely regulates presynaptic protein turnover and synaptic efficacy. *Curr. Biol.* 13 899–910. 10.1016/S0960-9822(03)00338-5 12781128

[B168] Svendsen C. N. Kew J. N. Staley K. Sofroniew M. V. (1994). Death of developing septal cholinergic neurons following NGF withdrawal in vitro: protection by protein synthesis inhibition. *J. Neurosci.* 14 75–87. 10.1523/JNEUROSCI.14-01-00075.1994 8283253 PMC6576871

[B169] Sze C. I. Bi H. Kleinschmidt-DeMasters B. K. Filley C. M. Martin L. J. (2000). Selective regional loss of exocytotic presynaptic vesicle proteins in Alzheimer’s disease brains. *J. Neurol. Sci.* 175 81–90. 10.1016/S0022-510X(00)00285-9 10831767

[B170] Tai H. C. Schuman E. M. (2008). Ubiquitin, the proteasome and protein degradation in neuronal function and dysfunction. *Nat. Rev. Neurosci.* 9 826–838. 10.1038/nrn2499 18931696

[B171] Takada K. Kanda T. Ohkawa K. Matsuda M. (1994). Ubiquitin and ubiquitin-protein conjugates in PC12h cells: changes during neuronal differentiation. *Neurochem. Res.* 19 391–398. 10.1007/BF00967315 8065495

[B172] Tanaka K. Matsuda N. (2014). Proteostasis and neurodegeneration: the roles of proteasomal degradation and autophagy. *Biochim. Biophys. Acta* 1843 197–204. 10.1016/j.bbamcr.2013.03.012 23523933

[B173] Terry R. D. Masliah E. Salmon D. P. Butters N. DeTeresa R. Hill R. (1991). Physical basis of cognitive alterations in Alzheimer’s disease: synapse loss is the major correlate of cognitive impairment. *Ann. Neurol.* 30 572–580. 10.1002/ana.410300410 1789684

[B174] Thrower J. S. Hoffman L. Rechsteiner M. Pickart C. M. (2000). Recognition of the polyubiquitin proteolytic signal. *EMBO J.* 19 94–102. 10.1093/emboj/19.1.94 10619848 PMC1171781

[B175] Tramutola A. Di Domenico F. Barone E. Perluigi M. Butterfield D. A. (2016). It is all about (U)biquitin: role of altered ubiquitin-proteasome system and UCHL1 in Alzheimer disease. *Oxid. Med. Cell. Longev.* 2016:2756068. 10.1155/2016/2756068 26881020 PMC4736377

[B176] Triaca V. Calissano P. (2016). Impairment of the nerve growth factor pathway driving amyloid accumulation in cholinergic neurons: the incipit of the Alzheimer’s disease story? *Neural Regen. Res.* 11 1553–1556. 10.4103/1673-5374.193224 27904476 PMC5116824

[B177] Triaca V. Sposato V. Bolasco G. Ciotti M. T. Pelicci P. Bruni A. C. (2016). NGF controls APP cleavage by downregulating APP phosphorylationat Thr668: relevance for Alzheimer’s disease. *Aging Cell* 15 661–672. 10.1111/acel.12473 27076121 PMC4933663

[B178] Tseng B. P. Green K. N. Chan J. L. Blurton-Jones M. LaFerla F. M. (2008). Abeta inhibits the proteasome and enhances amyloid and tau accumulation. *Neurobiol. Aging* 29 1607–1618. 10.1016/j.neurobiolaging.2007.04.014 17544172 PMC2664168

[B179] Tuszynski M. H. Tha L. Pay M. Salmon D. P. U. Hs Bakay R. (2005). A phase 1 clinical trial of nerve growth factor gene therapy for Alzheimer disease. *Nat. Med.* 11 551–555. 10.1038/nm1239 15852017

[B180] Tydlacka S. Wang C. E. Wang X. Li S. Li X. J. (2008). Differential activities of the ubiquitin-proteasome system in neurons versus glia may account for the preferential accumulation of misfolded proteins in neurons. *J. Neurosci.* 28 13285–13295. 10.1523/JNEUROSCI.4393-08.2008 19052220 PMC2662777

[B181] Upadhya S. C. Ding L. Smith T. K. Hegde A. N. (2006). Differential regulation of proteasome activity in the nucleus and the synaptic terminals. *Neurochem. Int.* 48 296–305. 10.1016/j.neuint.2005.11.003 16352375

[B182] Upadhya S. C. Hegde A. N. (2007). Role of the ubiquitin proteasome system in Alzheimer’s disease. *BMC Biochem.* 8(Suppl. 1):S12. 10.1186/1471-2091-8-S1-S12 18047736 PMC2106363

[B183] van Roessel P. Elliott D. A. Robinson I. M. Prokop A. Brand A. H. (2004). Independent regulation of synaptic size and activity by the anaphase-promoting complex. *Cell* 119 707–718. 10.1016/j.cell.2004.11.028 15550251

[B184] Viscomi M. T. D’Amelio M. Cavallucci V. Latini L. Bisicchia E. Nazio F. (2012). Stimulation of autophagy by rapamycin protects neurons from remote degeneration after acute focal brain damage. *Autophagy* 8 222–235. 10.4161/auto.8.2.18599 22248716

[B185] Watts R. J. Hoopfer E. D. Luo L. (2003). Axon pruning during Drosophila metamorphosis: evidence for local degeneration and requirement of the ubiquitin-proteasome system. *Neuron* 38 871–885. 10.1016/S0896-6273(03)00295-2 12818174

[B186] Webb J. L. Ravikumar B. Atkins J. Skepper J. N. Rubinsztein D. C. (2003). Alpha-synuclein is degraded by both autophagy and the proteasome. *J. Biol. Chem.* 278 25009–25013. 10.1074/jbc.M300227200 12719433

[B187] Wemmie J. A. Chen J. Askwith C. C. Hruska-Hageman A. M. Price M. P. Nolan B. C. (2002). The acid-activated ion channel ASIC contributes to synaptic plasticity, learning, and memory. *Neuron* 34 463–477. 10.1016/S0896-6273(02)00661-X 11988176

[B188] Whitehouse P. J. Price D. L. Clark A. W. Coyle J. T. DeLong M. R. (1981). Alzheimer disease: evidence for selective loss of cholinergic neurons in the nucleus basalis. *Ann. Neurol.* 10 122–126. 10.1002/ana.410100203 7283399

[B189] Willeumier K. Pulst S. M. Schweizer F. E. (2006). Proteasome inhibition triggers activity-dependent increase in the size of the recycling vesicle pool in cultured hippocampal neurons. *J. Neurosci.* 26 11333–11341. 10.1523/JNEUROSCI.1684-06.2006 17079661 PMC2665188

[B190] Wilson S. M. Bhattacharyya B. Rachel R. A. Coppola V. Tessarollo L. Householder D. B. (2002). Synaptic defects in ataxia mice result from a mutation in Usp14, encoding a ubiquitin-specific protease. *Nat. Genet.* 32 420–425. 10.1038/ng1006 12368914

[B191] Wong T. P. Debeir T. Duff K. Cuello A. C. (1999). Reorganization of cholinergic terminals in the cerebral cortex and hippocampus in transgenic mice carrying mutated presenilin-1 and amyloid precursor protein transgenes. *J. Neurosci.* 19 2706–2716. 10.1523/JNEUROSCI.19-07-02706.1999 10087083 PMC6786057

[B192] Wu C. W. Yeh H. H. (2005). Nerve growth factor rapidly increases muscarinic tone in mouse medial septum/diagonal band of Broca. *J. Neurosci.* 25 4232–4242. 10.1523/JNEUROSCI.4957-04.2005 15858049 PMC6725107

[B193] Yao I. Takagi H. Ageta H. Kahyo T. Sato S. Hatanaka K. (2007). SCRAPPER-dependent ubiquitination of active zone protein RIM1 regulates synaptic vesicle release. *Cell* 130 943–957. 10.1016/j.cell.2007.06.052 17803915 PMC3049808

[B194] Yaron A. Schuldiner O. (2016). Common and divergent mechanisms in developmental neuronal remodeling and dying back neurodegeneration. *Curr. Biol.* 26 R628–R639. 10.1016/j.cub.2016.05.025 27404258 PMC5086086

[B195] Yi J. J. Ehlers M. D. (2005). Ubiquitin and protein turnover in synapse function. *Neuron* 47 629–632. 10.1016/j.neuron.2005.07.008 16129392

[B196] Yi J. J. Ehlers M. D. (2007). Emerging roles for ubiquitin and protein degradation in neuronal function. *Pharmacol. Rev.* 59 14–39. 10.1124/pr.59.1.4 17329546

[B197] Zhai Q. Wang J. Kim A. Liu Q. Watts R. Hoopfer E. (2003). Involvement of the ubiquitin-proteasome system in the early stages of wallerian degeneration. *Neuron* 39 217–225. 10.1016/S0896-6273(03)00429-X 12873380

[B198] Zhao Y. Hegde A. N. Martin K. C. (2003). The ubiquitin proteasome system functions as an inhibitory constraint on synaptic strengthening. *Curr. Biol.* 13 887–898. 10.1016/S0960-9822(03)00332-4 12781127

[B199] Zhu H. Yan H. Tang N. Li X. Pang P. Li H. (2017). Impairments of spatial memory in an Alzheimer’s disease model via degeneration of hippocampal cholinergic synapses. *Nat. Commun.* 8:1676. 10.1038/s41467-017-01943-0 29162816 PMC5698429

