# Supplementary figures and images for: NGF-Dependent Changes in Ubiquitin Homeostasis Trigger Early Cholinergic Degeneration in Cellular and Animal AD-Model
Source: Front Cell Neurosci. 2018 Dec 13;12:487. doi: 10.3389/fncel.2018.00487 (PMC6300588; doi:10.3389/fncel.2018.00487)

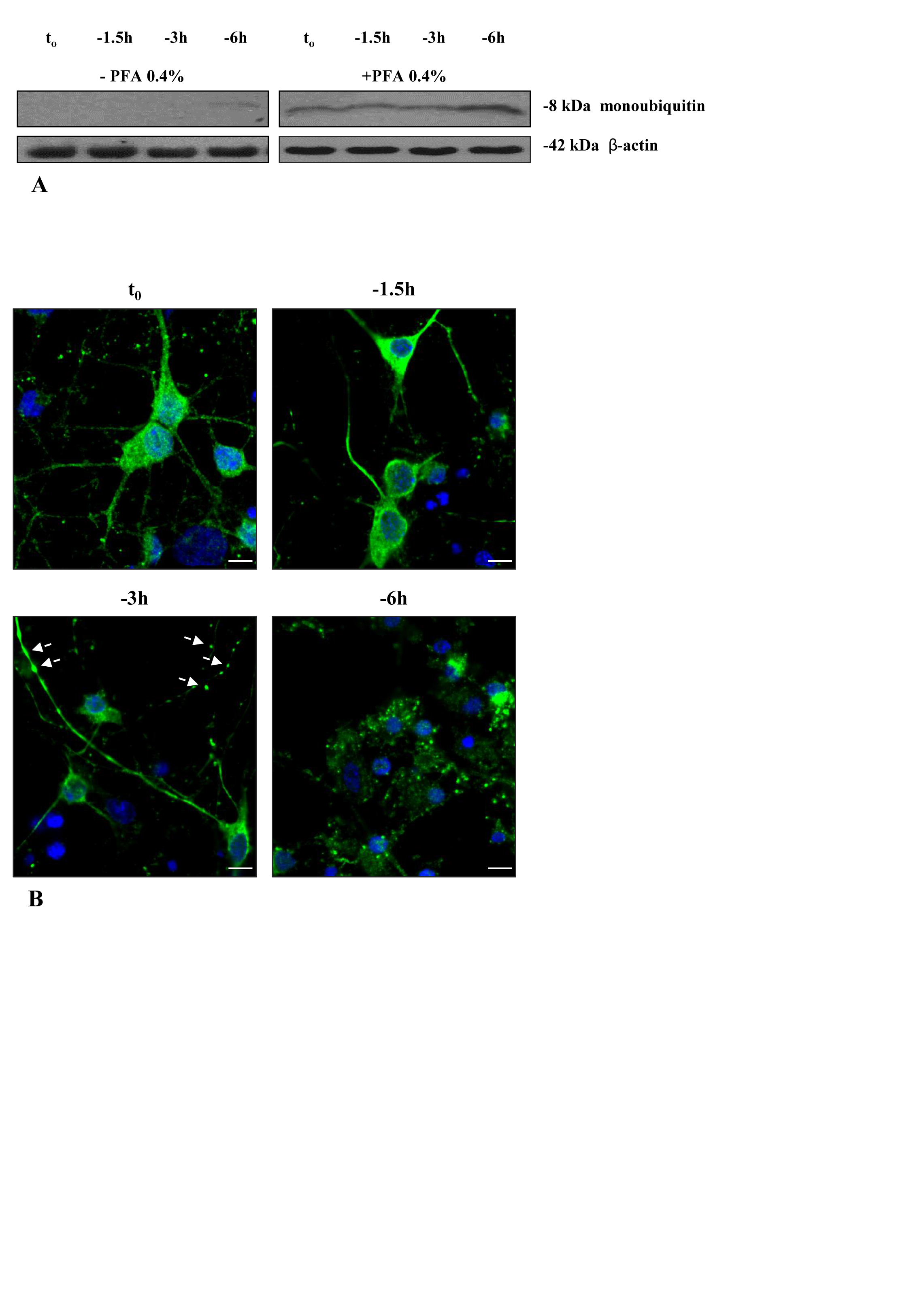

Supplement: FIGURE S1 — (A) Western blotting analysis was carried out on equal amounts of total protein extract (20 μg) from septal primary neurons cultured for 10–12 D.I.V. in defined medium supplemented with 0.2% B27, in the presence (t0) or absence (-1.5, -3, -6 h) of exogenously added NGF (100 ng/ml). Notice that cross-linking treatment of 0.1 μM filter with PFA 0.4% after transfer and before probing with anti-pan ubiquitin antibody (Dako antibody Z0458) increases the retention of low-molecular-weight monoubiquitin and improves the immunoreactivity signal. β-actin was used as loading control for each sample/lane. (B) Septal cholinergic-enriched cultures were grown continuously (10–12 D.I.V.) in 0.2% B27 media in the presence of exogenous NGF (100 ng/ml) from plating (t0) and then deprived of their trophic support for different periods of time (-1.5, -3, -6 h). Immunofluorescence staining with anti-pan ubiquitin antibody (Dako antibody Z0458) (green channel) followed confocal microscopy analysis was carried out to visualize the progressive formation of spheroid bodies (beading, varicosities) in nerve terminals (arrows) during the “dying-back” phenomenon triggered by NGF withdrawal. Images were representative of at least three independent experiments. Scale bar: 10 μM. [file Image_1.TIF]

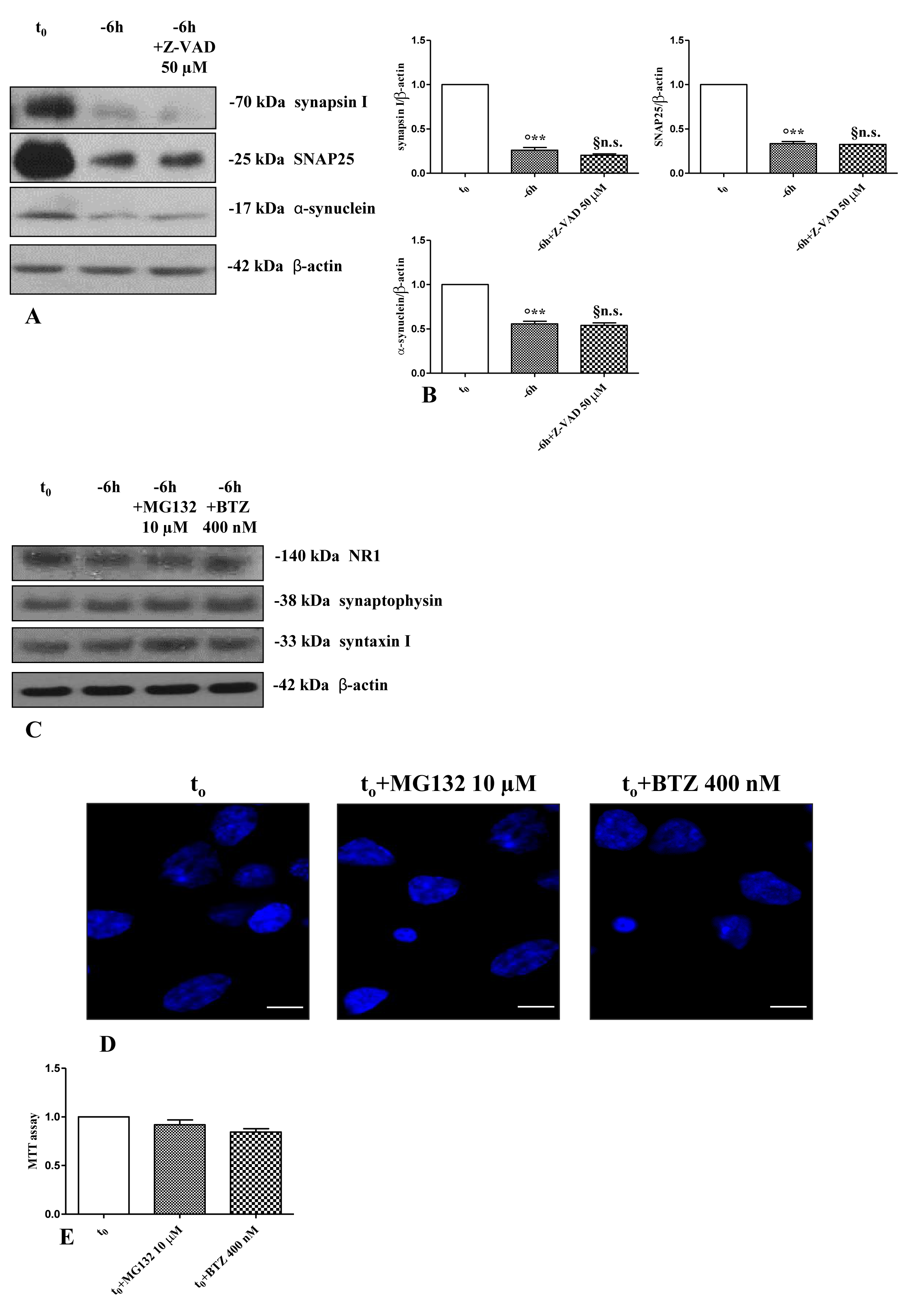

Supplement: FIGURE S2 — (A,B) Septal cholinergic-enriched cultures were grown continuously (10–12 D.I.V.) in 0.2% B27 media in the presence of exogenous NGF (100 ng/ml) from plating (t0) and then deprived of their trophic support for 6 h (-6 h) in the absence or presence of Z-VAD fmk (50 μM), a cell permeant irreversible pan-caspase inhibitor: Levels of SNAP25 and β-actin were assessed by Western blotting analysis (A) and densitometric quantification (B) was calculated as ratio of the value from control neurons (t0) and reported as mean ± SEM. Statistically significant differences were calculated at the respective experimental points by unpaired-two tailed t-Student’s test [∗∗p < 0.01 vs. t0 control neurons (°) and vs. -6 h NGF deprivation (§n.s., not significant)]. (C) Septal cholinergic-enriched cultures were grown continuously (10–12 D.I.V.) in 0.2% B27 media in the presence of exogenous NGF (100 ng/ml) from plating (t0) and then deprived of their trophic support for 6 h (-6 h) in the absence or presence of MG132 (10 μM) and BTZ (400 nM). Cell lysates were extracted and analyzed by Western blotting by probing for syntaxin I, NMDAR1 and synaptophysin. No change was detected on the protein expression level following -6 h NGF deprivation in the absence and in the presence of drugs. (D,E) Septal cholinergic-enriched cultures were grown continuously (10–12 D.I.V.) in 0.2% B27 media in the presence of exogenous NGF (100 ng/ml) from plating (t0) and treated with MG132 (10 μM) and BTZ (400 nM). DNA condensation-based assays with DAPI staining (D) detects no significant sign of apoptotic nuclei featured by of chromatin compaction. Scale bar: 10 μM. Cell viability was calculated by MTT assay after 6 h of incubation. The histogram (E) reports the neuronal survival calculated as the ratio of treated samples over untreated controls. Values are means of three experiments and no statistically significant differences were calculated by unpaired two-tailed t-Student’s test. [file Image_2.TIF]

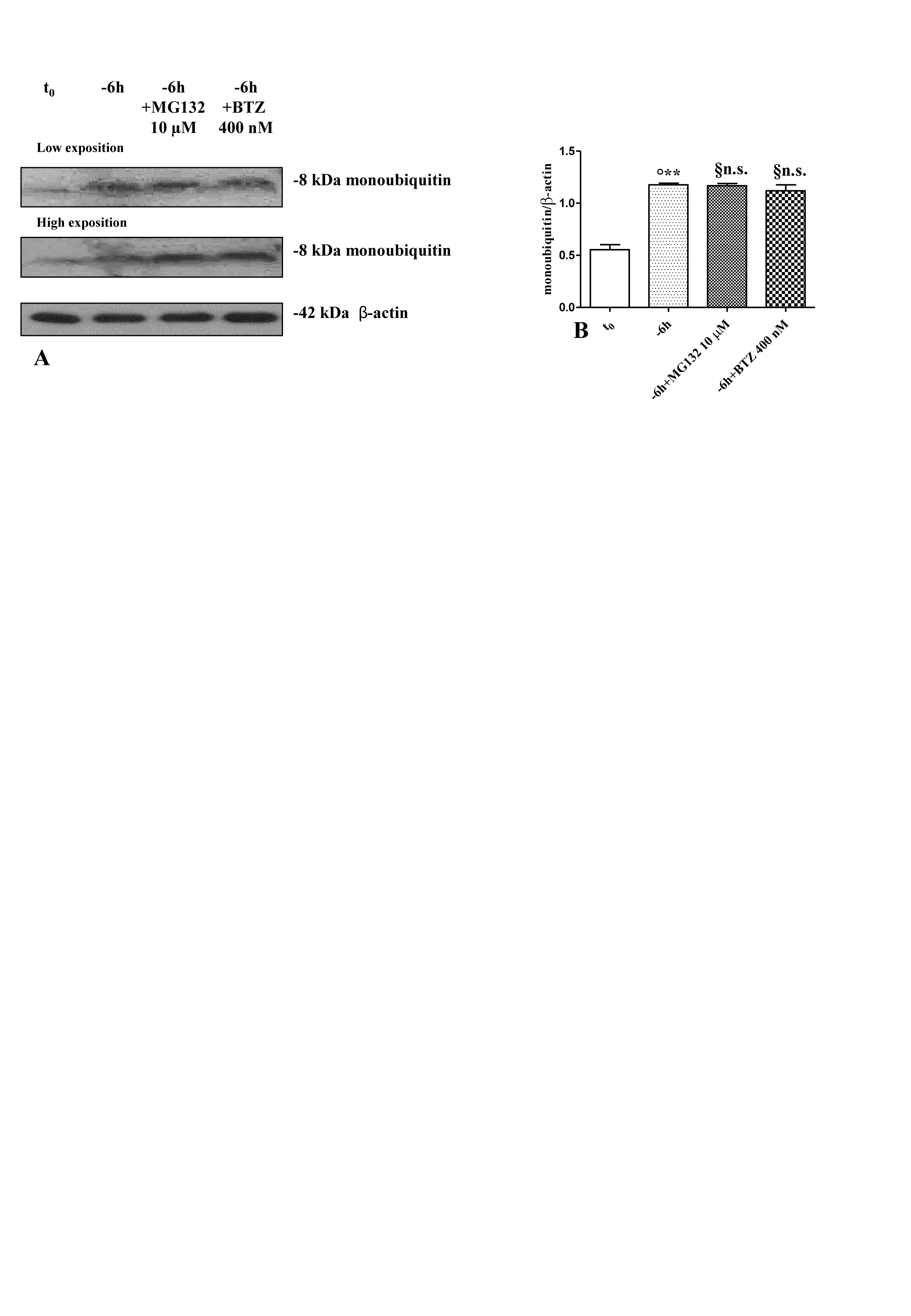

Supplement: FIGURE S3 — (A,B) Septal cholinergic-enriched cultures were grown continuously (10–12 D.I.V.) in 0.2% B27 media in the presence of exogenous NGF (100 ng/ml) from plating (t0) and then deprived of their trophic support for 6 h (-6 h) in the absence or presence of MG132 (10 μM) and BTZ (400 nM). Western blotting analysis on whole-cell lysates (40 μg) was performed to check the expression levels of free, monoubiquitin by probing with anti-pan ubiquitin antibody (Z0458 Dako). β-actin was used as internal control for samples loading (A) and densitometric quantification (B) was calculated as ratio of the value from control neurons (t0) and reported as mean ± SEM. Statistically significant differences were calculated by unpaired-two tailed t-Student’s test [∗∗p < 0.01 vs. t0 control neurons (°) and vs. -6 h NGF deprivation (§n.s., not significant)]. [file Image_3.TIF]

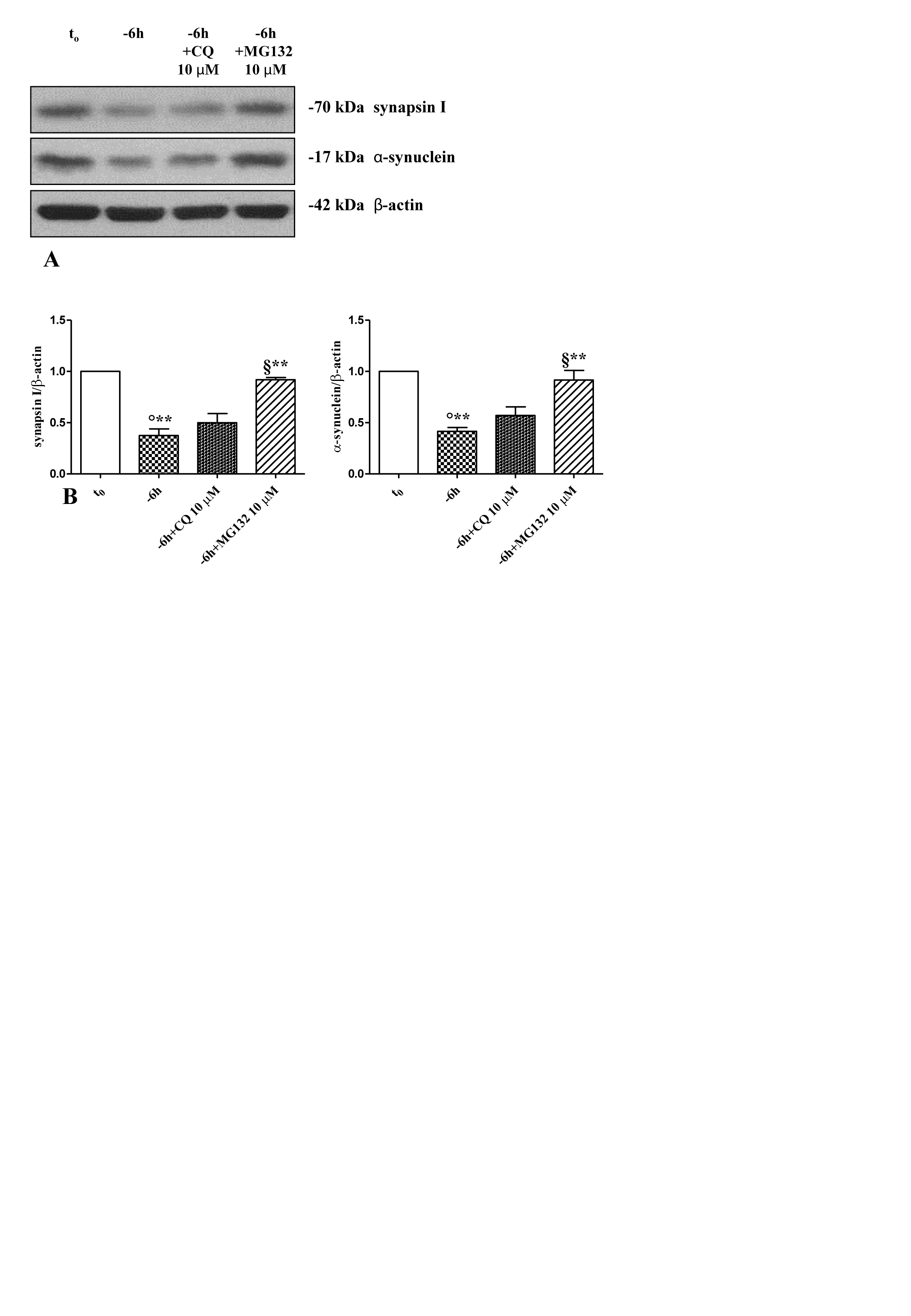

Supplement: FIGURE S4 — (A,B) Septal cholinergic-enriched cultures were grown continuously (10–12 D.I.V.) in 0.2% B27 media in the presence of exogenous NGF (100 ng/ml) from plating (t0) and then deprived of their trophic support for 6 h (-6 h) in the absence or presence of CQ (10 μM) and MG132 (10 μM). Western blotting analysis carried out on whole-cell extracts by probing for synapsin I, α-synuclein and β-actin (A) and relative densitometric quantification (B) were shown. Data were reported as mean ± SEM. and expressed ratio of the value from control neurons (t0). Statistically significant differences were calculated at the respective experimental points by unpaired-two tailed t-Student’s test [∗∗p < 0.01 vs. t0 control neurons (°) and vs. -6 h NGF deprivation (§)]. [file Image_4.TIF]

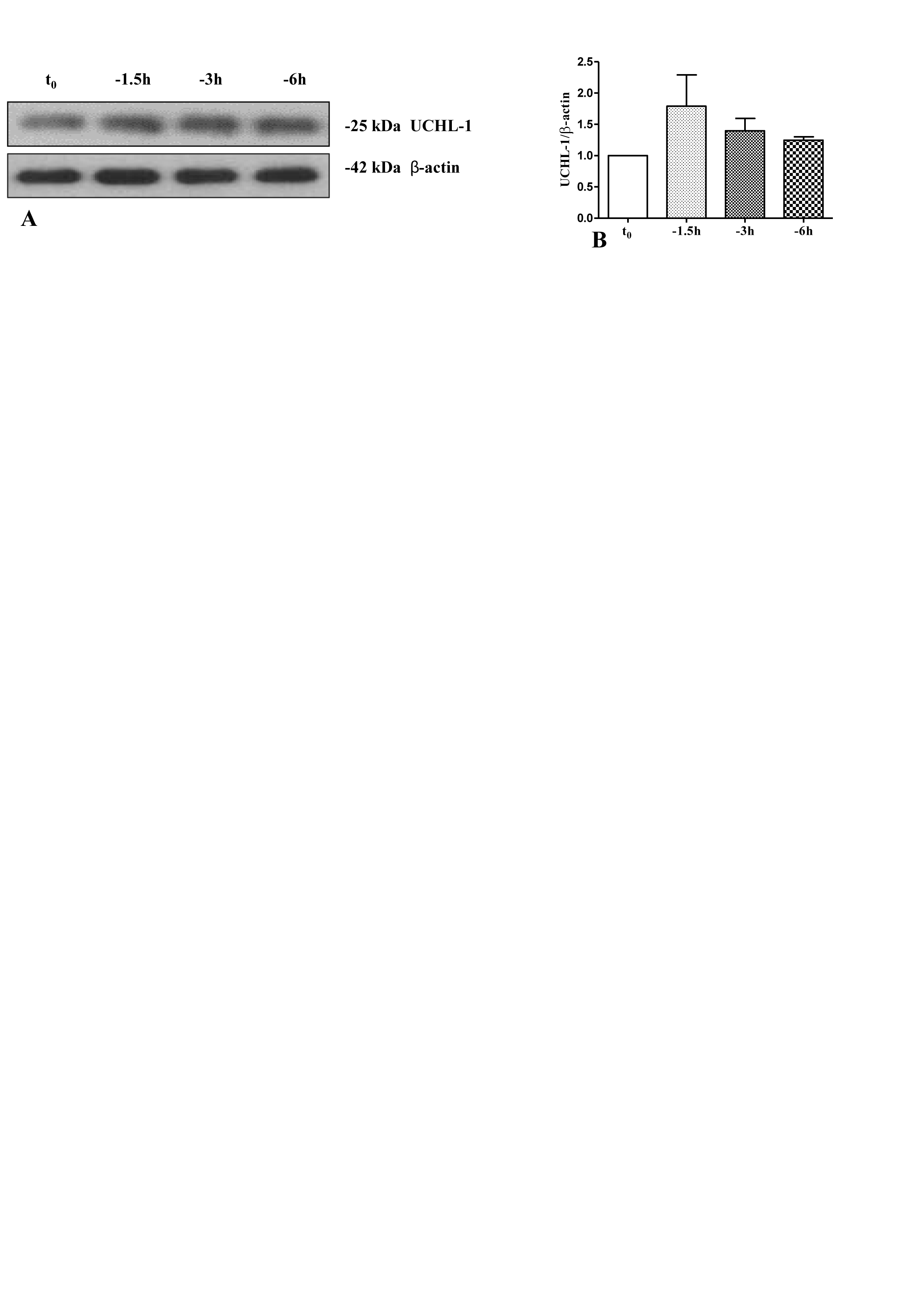

Supplement: FIGURE S5 — (A,B) Western blotting analysis for UCHL-1 (A) was carried out on equal amounts of total protein extract (20 μg) from septal primary neurons cultured for 10–12 D.I.V. in defined medium supplemented with 0.2% B27, in the presence (t0) or absence (-1.5, -3, -6 h) of exogenously added NGF (100 ng/ml). Relative densitometric quantification (B) by normalization on β-actin, which was used as internal control for samples loading, was reported. Data (n = 3) were reported as mean ± SEM. and expressed ratio of the value from control neurons (t0). Notice that there is in neuronal cultures an increase of the UCHL-1 expression level induced by the time-dependent NGF removal but values does not reach statistical significance by unpaired-two tailed t-Student’s test (-1.5 h p = 0.1750; -3 h p = 0.1424; -6 h, p < 0.0676 vs. t0 control neurons). [file Image_5.TIF]

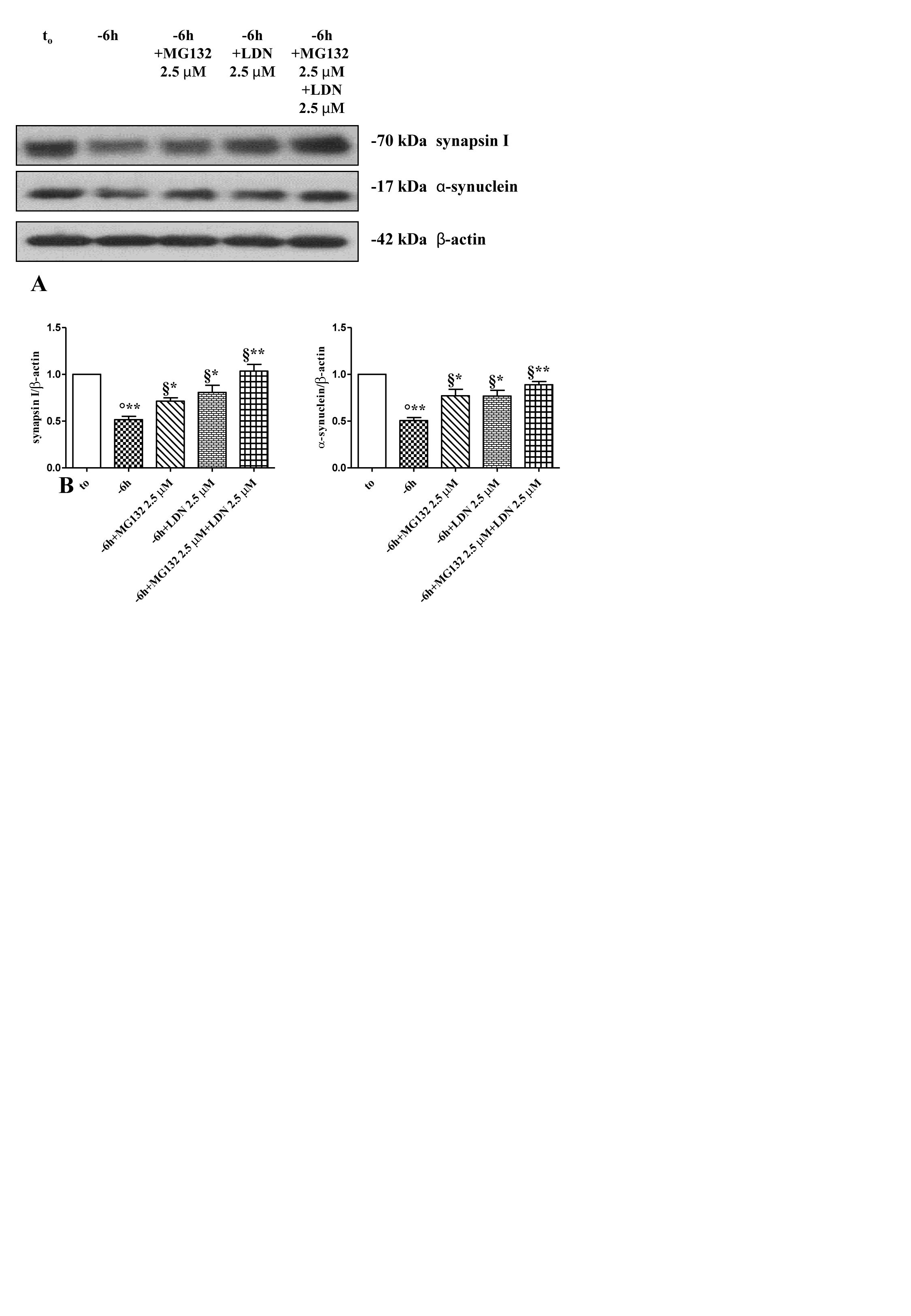

Supplement: FIGURE S6 — (A,B) Septal cholinergic-enriched cultures were grown continuously (10–12 D.I.V.) in 0.2% B27 media in the presence of exogenous NGF (100 ng/ml) from plating (t0) and then deprived of their trophic support for 6 h (-6 h) in the absence or presence of MG132 (2.5 μM), LDN (2.5 μM), and MG132 (2.5 μM)+LDN (2.5 μM) combination. Levels of synapsin I and synuclein were assessed by Western blotting analysis and β-actin was used as loading control (A). Relative densitometric quantification (B) was calculated as ratio of the value from control neurons (t0) and reported as mean ± SEM. Statistically significant differences were calculated at the respective experimental points by unpaired-two tailed t-Student’s test [∗p < 0.05, ∗∗p < 0.01 vs. t0 control neurons (°) and vs. -6 h NGF deprivation (§)]. [file Image_6.TIF]
